# Selenium Supplementation in Patients with Hashimoto Thyroiditis: A Systematic Review and Meta-Analysis of Randomized Clinical Trials

**DOI:** 10.1089/thy.2023.0556

**Published:** 2024-03-13

**Authors:** Valentina V. Huwiler, Stephanie Maissen-Abgottspon, Zeno Stanga, Stefan Mühlebach, Roman Trepp, Lia Bally, Arjola Bano

**Affiliations:** ^1^ Department of Diabetes, Endocrinology, Nutritional Medicine and Metabolism, Inselspital, Bern University Hospital, University of Bern, Bern, Switzerland.; ^2^ Division of Clinical Pharmacy and Epidemiology, Department of Pharmaceutical Sciences, University of Basel, Basel, Switzerland.; ^3^ Institute of Social and Preventive Medicine, University of Bern, Bern, Switzerland.

**Keywords:** Hashimoto thyroiditis, autoimmune thyroiditis, selenium, systematic review and meta-analysis, TSH, TPOAb

## Abstract

**Background:**

Hashimoto thyroiditis (HT) is the most common cause of hypothyroidism in iodine-sufficient areas. Selenium is an essential trace element required for thyroid hormone synthesis and exerts antioxidant effects. Therefore, it may be of relevance in the management of HT.

**Methods:**

We conducted a systematic review and meta-analysis of randomized controlled trials (RCTs) to evaluate the effect of selenium supplementation on thyroid function (thyrotropin [TSH], free and total thyroxine [fT4, T4], free and total triiodothyronine [fT3, T3]), thyroid antibodies (thyroid peroxidase antibodies [TPOAb], thyroglobulin antibodies [TGAb], thyrotropin receptor antibody [TRAb]), ultrasound findings (echogenicity, thyroid volume), immune markers, patient-reported outcomes, and adverse events in HT. The study protocol was registered on PROSPERO (CRD42022308377). We systematically searched MEDLINE, Embase, CINHAL, Web of Science, Google Scholar, and the Cochrane CENTRAL Register of Trials from inception to January 2023 and searched citations of eligible studies. Two independent authors reviewed and coded the identified literature. The primary outcome was TSH in patients without thyroid hormone replacement therapy (THRT); the others were considered secondary outcomes. We synthesized the results as standardized mean differences (SMD) or odds ratio (OR), assessed risk of bias using the Cochrane RoB 2 tool, and rated the evidence using the Grading of Recommendations Assessment, Development, and Evaluation (GRADE) approach.

**Results:**

We screened 687 records and included 35 unique studies. Our meta-analysis found that selenium supplementation decreased TSH in patients without THRT (SMD −0.21 [confidence interval, CI −0.43 to −0.02]; 7 cohorts, 869 participants; *I*^2^ = 0%). In addition, TPOAb (SMD −0.96 [CI −1.36 to −0.56]; 29 cohorts; 2358 participants; *I*^2^ = 90%) and malondialdehyde (MDA; SMD −1.16 [CI −2.29 to −0.02]; 3 cohorts; 248 participants; *I*^2^ = 85%) decreased in patients with and without THRT. Adverse effects were comparable between the intervention and control groups (OR 0.89 [CI 0.46 to 1.75]; 16 cohorts; 1339 participants; *I*^2^ = 0%). No significant changes were observed in fT4, T4, fT3, T3, TGAb, thyroid volume, interleukin (IL)-2, and IL-10. Overall, certainty of evidence was moderate.

**Conclusions:**

In people with HT without THRT, selenium was effective and safe in lowering TSH, TPOAb, and MDA levels. Indications for lowering TPOAb were found independent of THRT.

## Introduction

Hashimoto thyroiditis (HT), also referred to as chronic autoimmune or lymphocytic thyroiditis, is the most prevalent cause of hypothyroidism in iodine-sufficient areas.^1^ It affects ∼160 million people globally, with women being 4–10 times more susceptible than men.^1,2^ HT is characterized by chronic inflammation of the thyroid gland, elevated serum antibodies against thyroid antigens, and typical appearance on thyroid ultrasound.^1^ Once hypothyroidism develops, the current standard of care is lifelong thyroid hormone replacement therapy (THRT) with levothyroxine (LT4).^3^

Since several trace elements are essential for normal thyroid function, there is increasing interest in their supplementation for the management of HT, in particular the prevention of hypothyroidism.^4,5^ Besides iodine, one of the most discussed candidates is selenium.^6,7^ Selenium intake levels vary by region and are influenced by soil selenium content and selenium availability in the food chain, among other factors.^7^ Organ meats and seafoods are common sources of selenium, followed by muscle meats, cereals, and grains.^7^ The recommended daily allowance of selenium ranges between 55 and 70 μg for nonpregnant adults, which is often not reached, especially in Europe and some parts of China.^8,9^

Many thyroid enzymes are selenoproteins, such as the deiodinases that metabolize thyroid hormones and the glutathione peroxidases (GPX) that help to manage oxidative stress in the thyrocyte.^7,10^ Reduced selenium levels have been observed in patients with autoimmune thyroid disease, including HT.^11^ As a result, supplementing selenium in patients with HT has attracted much attention in recent decades.

So far, it has been suggested that selenium deficiency can exacerbate HT and the development of hypothyroidism.^2,12^ Therefore, preventing selenium deficiencies could be a promising approach to prevent or modify HT-associated hypothyroidism. However, findings from previous systematic reviews and meta-analyses on the effect of selenium supplementation in HT remain inconclusive^13–21^ due to factors such as a relatively small number of included studies,^13,16–19^ the inclusion of small and clinical heterogeneous populations (i.e., with and without THRT),^17^ and outdated data due to the publication of new trials.^13,14,16,18,21^ In addition, the differences across studies in patient characteristics, outcome definitions, inclusion criteria, and supplementation regimens may result in considerable heterogeneity (*I*^2^ ≥ 75%) and obscure the effect when results are statistically pooled.^22^ As a result, selenium supplementation is not currently considered in the guidelines for hypothyroidism or thyroid diseases of the American, European, and South American thyroid associations.^3,23–26^

In light of these inconclusive findings, we aimed to perform an updated systematic review and meta-analysis of randomized controlled trials (RCTs) investigating the effect of selenium supplementation on HT with particular attention to thyroid function, thyroid antibodies, ultrasound findings, immune markers, patient-reported outcome, and safety. We hypothesize that subgroup analyses will help us define indications for potential benefits.

## Materials and Methods

We conducted this systematic review and meta-analysis in accordance with the current Preferred Reporting Items for Systematic Reviews and Meta-Analyses (PRISMA) 2020 guidelines.^27^ The processes and techniques complied with the Cochrane Handbook for Systematic Reviews of Intervention.^22^ We prospectively registered the study protocol on PROSPERO (CRD42022308377). A detailed description of the Supplementary Materials and Methods section is included in Supplementary Data S1.

### Search strategy

We systematically searched the five electronic databases, MEDLINE, Embase, CINHAL, Web of Science, and Google Scholar, for publications and the CENTRAL Cochrane Library trial registry for ongoing and terminated trials from inception to April 5, 2022, and updated the search on November 14, 2023. We searched the Google Scholar database using the Publish or Perish software program^28^ and limited reports to the first most relevant 200 on April 5, 2022. A medical information specialist designed the search strategy and developed the search strings (Appendix A1 and Supplementary Data S1).

We identified all citations (forward citation search) and references (backward citation search) of the included reports using the citation chaser app by Haddaway et al. and checked them for eligibility.^29^

### Study eligibility and data extraction

The following criteria qualified the studies for eligibility: (1) RCT, (2) selenium supplementation, (3) overt or subclinical hypothyroid or euthyroid, (4) with or without THRT, and (5) HT patients. There were no restrictions on publication date, publication language, participants' age, intervention duration, selenium regimen (type and dose), outcomes, and lack or presence of selenium deficiency. Outcomes included thyroid function (thyrotropin [TSH], free and total thyroxine [fT4, T4], free and total triiodothyronine [fT3, T3]), thyroid antibodies (thyroid peroxidase antibodies [TPOAb], thyroglobulin antibodies [TGAb], thyrotropin receptor antibody [TRAb]), ultrasound findings (echogenicity, thyroid volume), immune markers, patient-reported outcomes, and adverse events. TSH was *a priori* defined as a primary outcome due to its importance in evaluating disease progression; the others were considered secondary outcomes. When outcomes were thyroid function parameters, we performed separate main analyses in patients without THRT as markers of residual endogenous thyroid hormone production.

The two authors, V.V.H. and S.M.-A., independently screened titles and abstracts for eligibility and subsequently full texts if eligibility was not clear. Interrater disagreements were resolved through consensus or discussion with a third independent reviewer. We extracted the relevant data with a standardized, predesigned coding sheet (details in Supplementary Data S1).

We coded multiple intervention groups from a single study separately, hereafter referred to as cohort, and combined cohorts that had a common control group to create single pairwise comparisons.^22^ Missing data were obtained directly from the authors. When the authors could not provide the missing results, we extracted the results from the diagrams using Rohatgi's WebPlotDigitizer, available at https://automeris.io/WebPlotDigitizer (2022), where possible.

### Data synthesis and statistical analyses

We performed a meta-analysis when at least two cohorts provided poolable results for an outcome, and a subgroup analysis and meta-regression when more than 10 cohorts were available.^22^ We conducted all the statistical analyses in R version 4.1.2 (R Core Team) using the metafor package.^30^

We statistically analyzed results as standardized mean differences (SMD) using the random-effects model, accounting for systematic variations in effect sizes between studies.^31,32^ For dichotomous outcomes, we analyzed odds ratios (ORs). Publication bias was assessed using funnel plot asymmetry,^33^ the Egger's regression,^34^ and rank correlation tests^35^ (details in Supplementary Data S1).

To identify sources of between-study heterogeneity, we performed stratified analyses, evaluating the potential impact of the following:

- Selenium dose (elemental selenium)
- Intervention duration
- Thyroid status (overt hypothyroidism, subclinical hypothyroidism, euthyroidism)
- Sex distribution (percentage of females)
- Participant age group (≥18 years, <18 years, mixed population)
- THRT
- Selenium status (severely selenium deficient, mildly selenium deficient, and selenium sufficient)
- Blinding
- Risk of publication bias.

In addition, we performed random-effects meta-regression analyses with selenium dose, intervention duration, sex distribution, and serum selenium levels as independent variables and effect estimates as dependent variables. *p*-Values below 0.05 were considered significant.^30^ To evaluate the impact of individual studies on the overall results, we visually inspected the Baujat plot^36^ and assessed the pooled risk estimates and heterogeneity after removing studies from the analyses one by one from (leave-one-out analysis) using the influence function.^37^

### Quality assessment

Two reviewers (V.V.H. and S.M.-A.) individually assessed the risk of bias in the included studies using the Cochrane Revised Risk of Bias Tool for randomized clinical trials (RoB 2).^38^

### GRADEing of evidence

The two authors, V.V.H. and S.M.-A., assessed the certainty of the evidence for each outcome in our meta-analysis for the longest time point using the Grading of Recommendations Assessment, Development, and Evaluation (GRADE) method.^39^

## Results

### Study selection

**FIG. 1.**
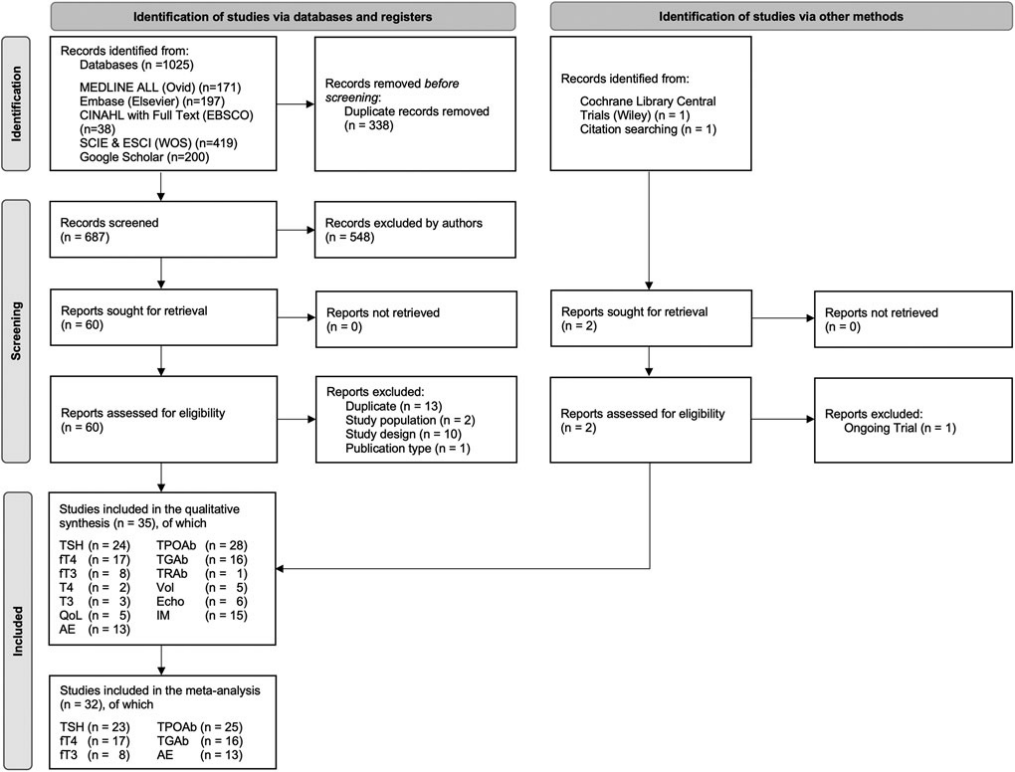
Flowchart for inclusion of the 35 studies adapted from the PRISMA 2020 statement.^27^ Six databases were systematically searched during the identification phase. Titles and/or abstracts were screened, and if records were eligible, the full text was assessed during the screening phase, resulting in the total number of studies included. Records refer to the title and/or abstract of a report. A report is a document that provides information about a study. AE, adverse event; Echo, ultrasound echogenicity; fT3, free triiodothyronine; fT4, free thyroxine; IM, immune markers including glutathione peroxidase, malondialdehyde, superoxide dismutase, total antioxidant capacity; PRISMA, Preferred Reporting Items for Systematic Reviews and Meta-Analyses; QoL, quality of life; T3, total triiodothyronine; T4, total thyroxine; TGAb, thyroglobulin antibodies; TPOAb, thyroid peroxidase antibodies; TRAb, thyrotropin receptor antibody; TSH, thyrotropin; Vol, thyroid volume.

We identified 1025 records, of which 687 remained after deduplication (312 duplicates). We checked the titles and abstracts, and if necessary the full texts, and identified 60 reports. In the full-text screening, 26 reports were excluded (Supplementary Table S1 in Supplementary Data S2). We identified one additional record by Shabalina and Fadeyev during the citation searching, which was not present in the databases searched.^40^ No additional study was included after cross-checking studies from previous systematic reviews and meta-analyses. A trial by Bonnema and colleagues was identified from the Cochrane Library Central,^41^ which confirmed that the recruitment was complete and publication was expected at the end of 2023. However, preliminary data were not available for this meta-analysis. Finally, we included 35 unique studies in the systematic review and 32 in the meta-analysis (Fig. 1).

### Study and participant characteristics

Table 1 provides an overview of the main characteristics of the 35 included studies. Seven studies included 2 cohorts, resulting in 42 cohorts.^40,42–47^ All studies were parallel RCTs published between 2002 and 2021. The study populations ranged from 31 to 364 participants and included children, adolescents, and adults. Two studies were conducted in children and adolescents aged ≤18 years^42,48^ and three in pregnant women.^49–51^ In 32 studies (91%), there was a clear female preponderance; 2 studies (6%) reported <50% female participants (36% and 40%),^52,53^ and 1 study (3%) did not report sex distribution^44^ (Table 1). Study durations varied from 2 to 12 months, with a mean maximum duration of 5.8 months (Table 1). The studies were conducted in Europe (*n* = 19),^42,43,45,48–51,54–65^ the Middle East (*n* = 4),^66–69^ Asia (*n* = 11),^40,46,47,52,53,70–75^ and South America (*n* = 1)^76^ (Table 1).

**Table 1.** Study and Participant Characteristics of the 35 Included Randomized Controlled Trials

First author (year)	Study population (age [years]; % female)	Duration (months)	Dose (μg/day), selenium compound	*N*	Blinding	Comparison	THRT (total study population)	Thyroid status and selenium status at study start	Outcomes included in this review
Anastasilakis (2012)^55^	18–80; 61.6%	3, 6	200, Selenium-methionine	40^a^	No	NA	Both	Euthyroid, mildly deficient	TSH,^b^ fT4,^b^ fT3,^b^ TPOAb,^b^ TGAb^b^
Balázs (2008)^64^	Mean 41.4 (I), 42.7 (C); 97.7%	12	200, Selenium-methionine	132	Double	Placebo	Yes	NA, NA	↓ TSH, fT4,^b^ fT3,^b^ ↓ TPOAb, = TGAb, = Vol
Bhuyan (2012)^75^	Mean 34 (I), 31 (C); 88.3%	3	200, Sodium selenite	60	Blinded	Placebo	Yes	Euthyroid or overt hypothyroid, NA	↓ TPOAb^c^
Bonfig (2010)^42^	7.6–16.4; 67.3%	12	(1) 100, (2) 200, Sodium selenite	49	No	No treatment	Yes	Overt hypothyroid, NA	= TSH, fT4,^b^ fT3,^b^ = TPOAb, = TGAb, = AE
Chakrabarti (2016)^52^	Mean 39.6 (I), 34.5 (C); 40%	6	200, Selenious acid	60	Participant blinded	Placebo	Yes	Overt hypothyroid, NA	↓ TSH, = fT4, ↓ MDA
De Farias (2015)^76^	20–58; 90.9%	3, 6	200, Selenium-methionine	43	Double	Placebo	Both	Euthyroid or subclinical hypothyroid, severely deficient	= TSH, fT4,^b^ = T3, = T4, = TPOAb, = TGAb, ↑ GPX
Duntas (2003)^56^	22–61; 86.2%	3, 6	200, Selenium-methionine	65	NA	Placebo	Yes	Subclinical hypothyroid, NA	= TSH, = fT4, = T3, = TPOAb, = TGAb
Eskes (2014)^65^	20–74; 100%	3, 6	200, Sodium selenite	61	Double	Placebo	No	Euthyroid, severely deficient	= TSH, = fT4, = TPOAb, = AE, ↑ SELENOP, = QoL
Esposito (2017)^58^	17–64; 100%	3, 6	166, Selenium-methionine	76	Blinded	Placebo	No	Euthyroid, NA	TSH,^b^ = fT4,^d^ ↑ fT3,^d^ = TPOAb, = Echo, = CXCL-10
Gärtner (2002)^57^	Mean 47.5; 100%	3	200, Sodium selenite	70	Blinded	Placebo	Yes	Euthyroid,^e^ severely deficient	= TSH, = fT4, = fT3, ↓ TPOAb, ↑ TGAb, = AE, ↑ QoL, ↓ Echo
Hu (2021)^70^	Mean 38.6; 88.9%	3, 6	200, Selenium yeast	90	No	No treatment	No	Euthyroid or subclinical hypothyroid, severely deficient	↓ TSH, = fT4, = fT3, = TPOAb, = TGAb, = AE
Kachouei (2018)^66^	18–60; 64.3%	3	200, Sodium selenite	70	Double	Placebo	Yes	Overt hypothyroid, mildly deficient	↑ GPX, ↑ SELENOP
Karanikas (2008)^54^	19–85; 100%	3	200, Sodium selenite	36	Blinded	Placebo	Yes	Euthyroid,^e^ severely deficient	= TSH, = fT4, = TPOAb, = TNF-α, = IFN-γ, = CD4, = CD8, = IL-2, = IL-4, = IL-10, = IL-13, ↑ QoL
Karimi (2019)^69^	15–78; 75.8%	3	200, Sodium selenite	66	Patient blinded	Placebo	Both	NA, mildly deficient	= TSH, = TPOAb, = TGAb, = AE
Krysiak (2012)^43^	18–60; 100%	3, 6	200, Selenium-methionine	164	Double	Placebo	(1) Yes (2) No	Euthyroid, NA	= TSH, = fT4, = fT3, ↓ TPOAb, ↓ TGAb (1), = TGAb (2), = AE
Kyrgios (2019)^48^	4.5–17.8; 80.3%	6	200, Selenium-methionine	71	Double	Placebo	Both	Euthyroid or overt hypothyroid, NA	= TSH, = fT4, ↓ TPOAb, ↓ TGAb, = Vol
Mahmoodianfard (2015)^67^	25–65; 100%	3	200, NA^f^	58	Double	Placebo	Yes	Overt hypothyroid. severely deficient	= TSH, = fT4, = T4, = fT3, = T3
Mahmoudi (2021)^68^	18–60; 88.1%	2	200, Selenium yeast	42	Double	Placebo	No	Subclinical hypothyroid, NA	TSH,^b^ T4,^b^ T3,^b^ TPOAb^b^
Mantovani (2019)^50^	18–45; 100%, Pregnant	(1) 6, (2) 12 (during/after pregnancy)	83, Selenium-methionine	45	Double	Placebo	Both	Euthyroid, severely deficient	TSH,^b^ fT4,^b^ fT3,^b^ = TPOAb (1), ↓ TPOAb (2), TGAb,^b^ = AE, = Echo, = Vol, = QoL
Mao (2016)^49^	NA; 100% Pregnant	2, 5	60, Selenium yeast	31	Double	Placebo	No	Euthyroid or subclinical hypothyroid, sufficient	TSH,^b^ fT4,^b^ = TPOAb
Nacamulli (2010)^59^	15–75; 85.5%	6, 12	80, Sodium selenite	76	Investigator blinded	No treatment	NA	Euthyroid and subclinical hypothyroid, NA	= TSH, = fT4, = TPOAb, = TGAb, ↓ Echo
Negro (2007)^51^	18–36; 100%; Pregnant	∼11 (0, 5, [12]^g^ Months after delivery)	200, Selenium-methionine	151	Double	Placebo	Both	Euthyroid, severely deficient	↓ TPOAb,^c,d^ = AE
Negro (2016)^44^	Mean 44 (I), 45 (C); NA	3	(1) 83 (2) 166 (3) 249, Selenium-methionine	80	Double	Placebo	No	Overt hypothyroid, NA	= TSH, = fT4, = CDs
Pilli (2015)^45^	21–65; 100%	6, 12	(1) 80 (2) 160, Selenium-methionine	60	Blinded	Placebo	No	Euthyroid, mildly deficient	= TSH,^d^ = TPOAb,^d^ = TGAb, = AE, = Echo, = GPX, = SELENOP, ↓ CXCL-9, ↓ CXCL-10, = CXCL-11 (1), ↓ CXCL-11 (2), ↓ TNF-α (1), = TNF-α (2), ↓ IFN-γ (1), = IFN-γ (2), = QoL
Pirola (2016)^61^	18–65; 84.9%	4	83, Selenium-methionine	192	NA	No treatment	No	Subclinical hypothyroid, NA	= TSH, = fT4, ↓ TPOAb
Preda (2017)^62^	Mean 46.2 (I), 50.5 (C); 100%	3	100, Selenium-methionine	100	NA	NA	NA	Euthyroid, sufficent	↓ TSH, = TPOAb, = GPX
Shabalina (2019)^40^	20–40; 100%	3, 6, 9, 12	200, Selenium-methionine	51	No	No treatment	NA	(1) Euthyroid (2) Subclinical hypothyroid, NA	↓ TSH, ↑ fT4 (1), = fT4 (2), = fT3, = TPOAb,^d^ = AE, = Echo, = Vol
Sun (2021)^71^	20–64; 68.1%	3	100, Selenium yeast	129	No	No treatment	Yes	Overt hypothyroid, NA	= TSH,^d^ = T4, = T3, = TPOAb, = TGAb, = AE, ↓ IL-2, ↑ IL-10, ↓ TNF-α
Tian (2020)^72^	>18 (Mean 40.2); 62.5%	3	200, Selenium yeast	32	NA	Placebo	NA	Euthyroid, mildly deficient	= TSH, = TPOAb, = TGAb, = AE, ↓ MDA, ↑ SOD, ↑ TAC
Turker (2006)^63^	15–77; 100%	3	200, Selenium-methionine	88^a^	Blinded	Placebo	Yes	Euthyroid, NA	TSH,^b^ fT4,^b^ fT3,^b^ = TPOAb, = TGAb
Wang (2018)^46^	15–70; 100%	3, 6	200, Selenium yeast	364	Double	Placebo	(1) No (2) Yes	(1) Euthyroid or subclinical hypothyroid (2) Overt hypothyroid, mildly deficient	= TSH, = fT4 (1), ↓ fT4 (2), = TPOAb, = AE, ↓ MDA, ↑ GPX
Wu (2018)^73^	20–71; 51.3%	2	200, Selenium yeast	80	No	No treatment	Yes	NA, NA	TSH,^b^ TPOAb,^b^ TGAb,^b^ ↑ CD4/CD8, ↑ CD4/CD3, ↑ CD8/CD3
Yu (2017)^74^	10–64; 93.3%	3	400, Selenium yeast	60	No	No treatment	Yes	(1) Euthyroid (2) Overt hypothyroid, severely deficient	TPOAb,^b^ TGAb,^b^ = AE, ↓ IL-2, = IL-10
Zhang (2020)^53^	32–65; 36.2%	4	200, Sodium selenite	94	No	No treatment	Yes	Overt hypothyroid, NA	= fT4, ↑ fT3, ↓ TPOAb, ↓ TGAb, = AE, ↓ TRAb, ↑ CD3, ↑ CD4, ↓ CD8, ↑ CD4/CD8
Zhu (2012)^47^	15–70; 100%	3, 6	200, Selenium yeast	134	Blinded	Placebo	NA	(1) Euthyroid or subclinical hypothyroid (2) Overt hypothyroid, mildly deficient	TSH,^b^ fT4,^b^ = TPOAb (1), ↓ TPOAb (2)

^a^ Only participants receiving same intervention throughout the entire study considered.

^b^ Results not available.

^c^ Data estimated from other studies.

^d^ Results extracted from graphs.

^e^ Participants already on THRT at study start and therefore reported euthyroid at study start.

^f^ One intervention group and its corresponding comparison group additionally received 30 mg zinc per day.

^g^ Time point 12 months after delivery was not included in our analysis to increase comparability with other studies.

↑, Significant increase; ↓, significant decrease; = , no significant effect; (1) Cohort 1; (2) Cohort 2; (C) Control group; (I) Intervention group receiving selenium supplementation; AE, adverse event; Echo, ultrasound echogenicity; fT3, free triiodothyronine; fT4, free thyroxine; GPX, glutathione peroxidases; IFN-γ, interferon-gamma; IL, interleukin; MDA, malondialdehyde; NA, not reported in publication; QoL, quality of life; SELENOP, selenoprotein P; SOD, superoxide dismutase; T3, total triiodothyronine; T4, total thyroxine; TAC, total antioxidant capacity; TGAb, thyroglobulin antibodies; THRT, thyroid hormone replacement therapy; TNF-α, tumor necrosis factor-alpha; TPOAb, thyroid peroxidase antibodies; TRAb, thyrotropin receptor antibody; TSH, thyrotropin; Vol, thyroid volume.

In total, 18 studies (51%) assessed the serum selenium levels at baseline. Nine studies (50%) were severely selenium deficient,^50,51,54,57,65,67,70,74,76^ seven (39%) mildly selenium deficient,^45–47,55,66,69,72^ and two selenium sufficient (11%)^49,62^ (Supplementary Table S4 in Supplementary Data S2).

Of the 35 studies, 30 (86%) diagnosed HT using TPOAb presence. The diagnostic thresholds for TPOAb levels varied (Supplementary Table S2 in Supplementary Data S2). Euthyroidism, subclinical hypothyroidism, and overt hypothyroidism were consistently defined based on TSH and fT4 levels, with euthyroidism defined as normal TSH levels and normal fT4 levels, subclinical hypothyroidism defined as elevated TSH levels with normal fT4 levels, and hypothyroidism defined as elevated TSH levels with decreased fT4 levels. However, the reference ranges for fT4 and fT3 levels varied across studies. The TSH reference ranges were predominantely consistent, mostly below or equal to 4 mIU/L, with a few studies extending the range up to 5 mIU/L, which could be due to assay differences. Eight studies (23%) did not report the TSH reference ranges (Supplementary Table S3 in Supplementary Data S2).

### Effect of selenium supplementation on thyroid function

A total of 32 cohorts investigated the effect of selenium supplementation on at least one thyroid parameter (TSH, fT4, fT3, T4, T3) (Table 1).

#### Thyrotropin

**FIG. 2.**
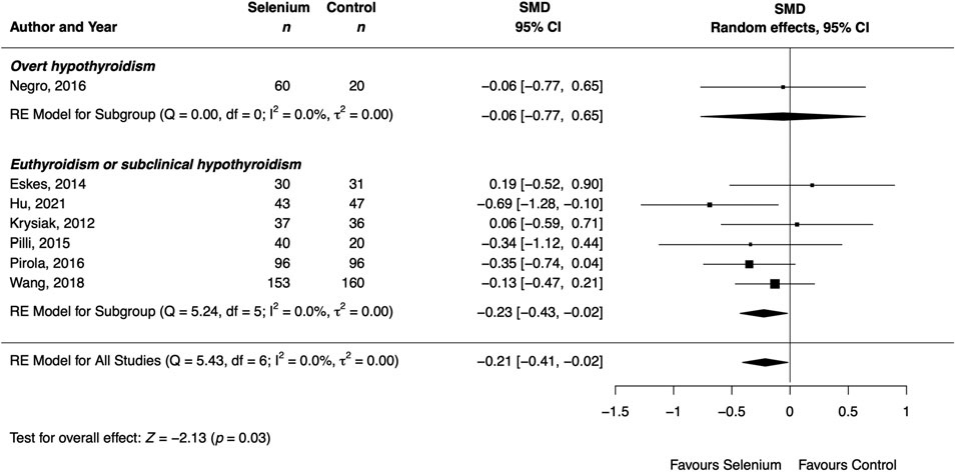
Effect of selenium supplementation on TSH (mIU/L), stratified by thyroid status, in patients with Hashimoto thyroiditis without thyroid hormone replacement therapy (*n* = 869). Black rectangles represent SMD for each study; the size of the rectangle is proportional to the weight of the study for the pooled effect. Horizontal lines indicate CI. The black diamond summarizes the pooled SMD data. CI, confidence interval; Control, Control group receiving placebo or nothing; SMD, standardized mean differences.

From the 11 cohorts reporting on TSH levels in participants without THRT, one observed a decrease^70^ and ten observed no change^43–46,58,61,65^ in TSH levels after selenium supplementation compared with the control group. Among the 20 cohorts reporting on TSH levels in participants with THRT or unspecified THRT, 4 observed a decrease,^40,52,62,64^ 1 an increase,^66^ and 15 no change in TSH levels after selenium supplementation compared with the control group. The meta-analysis demonstrated a significant decrease in TSH levels after selenium supplementation compared with the control group in patients without THRT (SMD −0.21 [confidence interval, CI −0.41 to −0.02]; 7 cohorts; 869 participants; *I*^2^ = 0%) (Table 2 and Fig. 2).

**Table 2.** Summary of Meta-Analysis Results of Thyroid Function (Thyrotropin, Free Thyroxine, Free Triiodothyronine), Thyroid Antibodies (Thyroid Peroxidase Antibodies, Thyroglobulin Antibodies), Adverse Events, Ultrasound Findings (Thyroid Volume), and Immune Markers (Interleukin-2, Interleukin-10, Malondialdehyde)

Outcome	Included cohorts	No. of participants	Pooled effect estimate [CI]	Heterogeneity* I*^2^ (%)	Publication bias		Quality of evidence
					Egger's (*p*)	Rank's (*p*)	
Thyroid function (effect estimate reported as SMD)							
TSH all	26	2063	−0.21 [−0.43 to 0.01]	59	0.71	0.76	Moderate
Without THRT	7	869	**−0.21 [−0.41 to −0.02]**	0	0.76	0.76	—
fT4 all	21	1664	0.05 [−0.15 to 0.25]	33	0.11	0.46	Moderate
Without THRT	7	623	0.16 [−0.06 to 0.39]	0	0.82	0.88	—
fT3 all	11	658	0.51 [−0.11 to 1.13]	84	0.83	0.94	Very low
Without THRT	3	239	1.01 [−0.60 to 2.63]	95	0.24	1.00	—
T4	3	187	−0.02 [−0.42 to 0.39]	0	—	—	—
T3	4	252	−0.11 [−0.48 to 0.26]	0	—	—	—
Thyroid antibodies (effect estimate reported as SMD)							
TPOAb	29	2358	**−0.96 [−1.36 to −0.56]**	90	0.24	**0.04**	Low
TGAb	17	1283	−0.27 [−0.59 to 0.06]	74	0.52	0.71	Low
AEs (effect estimate reported as OR)							
	16	1339	0.89 [0.46 to 1.75]	0	0.22	0.08	Moderate
Ultrasound findings (effect estimate reported as SMD)							
Thyroid volume	4	182	−0.14 [−0.57 to 0.28]	0	—	—	—
Immune markers (effect estimate reported as SMD)							
IL-2	3	189	−0.68 [−1.44 to 0.09]	59	—	—	—
IL-10	3	189	0.20 [−0.21 to 0.61]	0	—	—	—
MDA	3	248	−1.16 [−2.29 to −0.03]	85	—	—	—

Bold values indicate significant results.

CI, confidence interval; OR, odds ratio; SMD, standardized mean difference.

The TSH levels in the overall population remained unchanged (SMD −0.21 [CI −0.43 to 0.01]; 26 cohorts; 2063 participants; *I*^2^ = 59%) (Table 2 and Supplementary Fig. S1 in the Supplementary Data S2), with consistent results after restricting the analysis to patients with THRT (SMD −0.24 [CI −0.65 to 0.16]; 12 cohorts; 794 participants; *I*^2^ = 64%) (Supplementary Fig. S2 in Supplementary Data S2). Results remained consistent or strengthened after analyzing only (1) patients with euthyroidism or subclinical hypothyroidism, (2) adults, and (3) those receiving selenium for <6 months (Supplementary Table S5 and Supplementary Fig. S18 in Supplementary Data S2). The selenium compound significantly affected the results (*p* = 0.03). No outlier cohort was identified (Supplementary Fig. S23 in Supplementary Data S2). Nevertheless, due to missing data in the original publication and conflicting result estimates, we excluded the study by Esposito et al.^58^ We rated quality of evidence as moderate (Supplementary Table S6 in Supplementary Data S2).

#### Free thyroxine

From the 23 cohorts that reported fT4 levels, one observed an increase,^46^ one a decrease,^40^ and 21 no change in fT4 levels after selenium supplementation. The meta-analysis showed no significant increase in fT4 levels after selenium supplementation compared with the control group (SMD 0.05 [CI −0.15 to 0.25]; 21 cohorts; 1664 participants; *I*^2^ = 33%) (Table 2 and Supplementary Fig. S3 in Supplementary Data S2), with similar results after pooling cohorts without THRT and performing other subgroup analyses (Table 2 and Supplementary Figs. S4 and S5 in Supplementary Data S2). Notably, restricting the analyses to adult populations (i.e., excluding the four cohorts including patients younger than 18 years) revealed an increase in fT4 levels postselenium supplementation (SMD 0.19 [CI 0.01 to 0.38]; 17 cohorts; 1152 participants; *I*^2^ = 0%) (Supplementary Table S6 and Supplementary Fig. S19 in Supplementary Data S2).

The participants' age group and thyroid status significantly influenced the results (*p*-value <0.01 and = 0.03, respectively) (Supplementary Table S6 in Supplementary Data S2). Removing the two outlier cohorts^46,61^ led to similar results (Supplementary Fig. S23 in Supplementary Data S2). We rated quality of evidence as moderate (Supplementary Table S7 in Supplementary Data S2).

#### Free triiodothyronine

fT3 levels increased significantly in 2 out of 11 cohorts (18%) after intervention,^53,58^ while no significant effect was observed in the remaining nine cohorts (82%). The meta-analysis showed no significant increase in fT3 levels after selenium supplementation compared with the control group (SMD 0.51 [CI −0.11 to 1.13]; 11 cohorts; 658 participants; *I*^2^ = 84%) (Table 2 and Supplementary Fig. S6 in Supplementary Data S2), with similar results when restricting the analyses to cohorts without THRT (Table 2 and Supplementary Figs. S7 and S8 in Supplementary Data S2). Subgroup analysis did not reveal any significant influencing factors on the outcome (Supplementary Table S5 and Supplementary Fig. S22 in Supplementary Data S2). Removing the outlier cohort^58^ returned comparable results (Supplementary Fig. S23). We rated quality of evidence as very low (Supplementary Table S6 in Supplementary Data S2).

#### Total thyroxine and total triiodothyronine

T4 was reported in three^67,71^ and T3 in four cohorts,^56,67,71^ showing no significant difference between the selenium-treated and control groups (Supplementary Figs. S9 and S10 in Supplementary Data S2).

### Effect of selenium supplementation on thyroid antibodies

Our systematic review included 34 cohorts that investigated the impact of selenium supplementation on thyroid antibodies such as TPOAb, TGAb, and TRAb.

#### Thyroid peroxidase antibodies

**FIG. 3.**
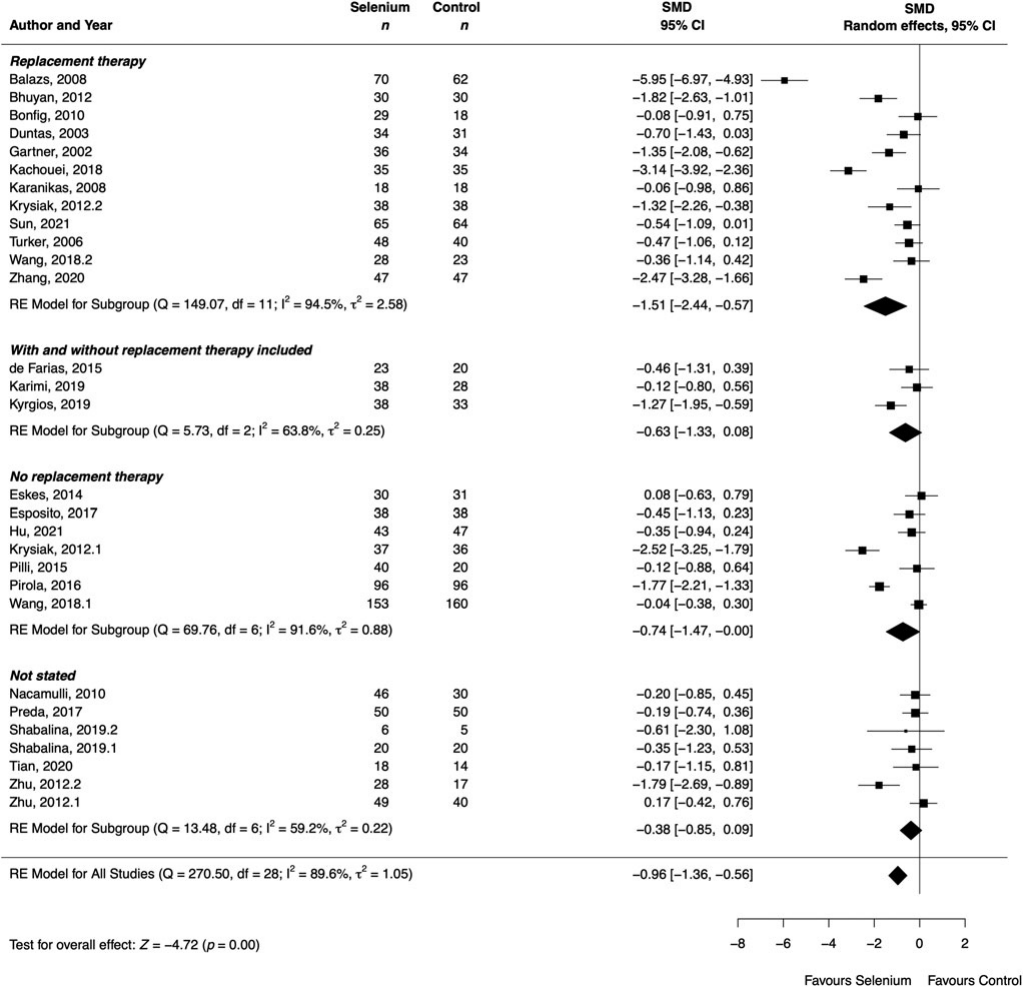
Effect of selenium supplementation on TPOAb (IU/mL), stratified by thyroid hormone replacement therapy, in Hashimoto thyroiditis (*n* = 2358). Black rectangles represent SMD for each study; the size of the rectangle is proportional to the weight of the study for the pooled effect. Horizontal lines indicate CI. The black diamond summarizes the pooled SMD data. (1)/(2) Indicate cohorts 1 and 2 of the study.

Of the 31 cohorts that reported on TPOAb, 10 (32%) observed a significant decrease in TPOAb titers,^43,47,48,53,57,61,64,66,75^ while 21 (68%) found no significant effect after selenium supplementation compared with the control group. The meta-analysis showed a significant decrease in TPOAb (SMD −0.96 [CI −1.36 to −0.56]; 29 cohorts; 2358 participants; *I*^2^ = 90%) (Fig. 3) after selenium supplementation. The results remained similar or even stronger after restricting the analyses to (1) patients with overt hypothyroidism, (2) adults, (3) those receiving THRT, (4) those using selenium doses above 100 μg/day, (5) those using selenomethionine, and (6) those blinded to treatment (Supplementary Table S5 and Supplementary Fig. S21 in Supplementary Data S2). The thyroid status of the participants significantly influenced the results (*p* = 0.03) (Supplementary Table S5 in Supplementary Data S2).

Removing the outlier cohort^64^ returned comparable results (Supplementary Fig. S23 in Supplementary Data S2). We rated quality of evidence as low (Supplementary Table S6 in Supplementary Data S2).

In pregnant women, TPOAb titers decreased significantly at delivery^51^ and 5 or 6 months postpartum,^50,51^ but no significant effect was observed during gestation^49,50^ (Supplementary Fig. S11 in Supplementary Data S2).

#### Thyroglobulin antibodies

For TGAb, 4 cohorts (21%) reported a decrease,^43,48,53,66^ 14 (74%) reported no change, and 1 (5%) reported an increase^57^ after selenium supplementation compared with control. Meta-analysis revealed no significant effect of selenium supplementation on TGAb (SMD −0.27 [CI −0.59 to 0.06]; 17 cohorts; 1283 participants; *I*^2^ = 74%) (Table 2 and Supplementary Fig. S12 in Supplementary Data S2). Notably, a decrease in TGAb levels was observed in HT patients without THRT (Supplementary Table S5 and Supplementary Fig. S22 in Supplementary Data S2). No outlier cohort was detected (Supplementary Fig. S23 in Supplementary Data S2). We rated quality of evidence as low (Supplementary Fig. S4 in Supplementary Data S2).

#### Thyrotropin receptor antibody

One study assessed TRAb and found a significant decrease in the selenium-treated compared with the control group.^53^

### Effect of selenium supplementation on ultrasound findings

#### Thyroid echogenicity

Eight cohorts investigated the effect of selenium on ultrasound echogenicity. Thyroid echogenicity decreased significantly after selenium supplementation (25% and 50% of participants) compared with the control group (5% and 12% of participants) in two cohorts (33%)^57,59^ with no effect in the other six (67%).^40,45,50,58^

#### Thyroid volume

Seven cohorts evaluating the effect of selenium on thyroid volume did not find significant results.^40,45,48,50,64^ The meta-analysis of the poolable cohorts indicated the same effect (SMD −0.14 [CI −0.57 to 0.28]; 4 cohorts; 182 participants; *I*^2^ = 0%) (Supplementary Fig. S13 in Supplementary Data S2).

### Effect of selenium supplementation on immune markers

In total, 18 cohorts assessed the inflammatory and antioxidant markers such as interleukins (ILs), chemokines, cytokines, and selenoproteins, with mixed results across studies.

#### Interleukins

Two cohorts reported decreases in IL-2,^71,74^ while one cohort reported no effect.^54^ The meta-analysis revealed no significant effect of selenium supplementation on IL-2 (SMD −0.68 [CI −1.44 to 0.09]; 3 cohorts; 189 participants; *I*^2^ = 59%) (Supplementary Fig. S14 in Supplementary Data S2). IL-10 increased in one cohort^71^ and remained unchanged in two others.^54,74^ The meta-analysis indicated no significant effect of selenium supplementation on IL-10 (SMD 0.20 [CI −0.21 to 0.61]; 3 cohorts; 189 participants; *I*^2^ = 0%) (Supplementary Fig. S15 in Supplementary Data S2).

#### Cytokines

The effect on interferon-gamma (IFN-γ) and tumor necrosis factor-alpha (TNF-α) was not consistent across studies.^45,54,71^

#### Chemokines

CXCL-9, -10, and -11 decreased in two cohorts^45^ and showed no effect in another.^58^

#### Antioxidant and oxidant markers

Malondialdehyde (MDA) decreased consistently in all four cohorts.^46,52,72^ The meta-analysis demonstrated a significant decrease in MDA after selenium supplementation (SMD −1.16 [CI −2.29 to −0.03]; 3 cohorts; 148 participants; *I*^2^ = 85%) (Supplementary Fig. S16 in Supplementary Data S2). Four cohorts reported an increase in GPX activity,^46,70,76^ while three showed no change.^45,62^ One cohort reported an increase in both superoxide dismutase (SOD) activity and total antioxidant capacity (TAC).^72^ Selenoprotein P (SELENOP) increased significantly in two cohorts^65,70^ but remained unchanged in two cohorts.^45^ Clusters of differentiation were found to be unaffected in three cohorts.^54,60^ However, two cohorts reported consistent increases in the CD4/CD8 ratio,^53,73^ CD4/CD3 ratio,^73^ and CD3^53^ (Table 1).

### Effect of selenium supplementation on patient-reported outcomes

A total of six cohorts evaluated the effect of selenium supplementation on well-being in HT patients.^45,50,54,57,65^ Of these, two cohorts measuring well-being with either an unspecified questionnaire or the short form 12 health survey (SF-12) questionnaire reported a higher percentage of improvement in well-being in selenium-treated patients compared with controls.^54,57^ The other four cohorts, measuring well-being with the short form 36 health survey questionnaire (*n* = 3)^45,65^ or the SF-12 questionnaire (*n* = 1),^50^ found no significant difference in quality of life between the two cohorts. All six cohorts were blinded.

### Safety of selenium supplementation

A total of 18 cohorts evaluated the incidence of adverse events in the study populations.^40,42,43,45,46,50,51,53,54,57,65,70–72,74^ Of these, 13 cohorts reported no adverse events. Five cohorts listed between 2 and 19 adverse events, including nausea, vomiting, fever, dizziness, chest tightness, bloating, gastrointestinal problems, hair loss, and miscarriages.^43,51,65,71^ None of the studies assessed events for severity and causality as recommended by the ICH E2A guidelines.^77^ Our meta-analysis found no significant difference in adverse events between the selenium and control groups in the studies with adverse events (OR 0.89 [CI 0.46 to 1.75]; 16 cohorts; 1339 participants; *I*^2^ = 0%) (Supplementary Fig. S17 in Supplementary Data S2). We rated quality of evidence as moderate (Supplementary Table S6 in Supplementary Data S2).

### Risk-of-bias assessment

Of the 31 studies reporting on TSH (i.e., our primary outcome), 12 (39%) raised some concerns, while 19 (61%) had a high risk of bias (Supplementary Table S7 in Supplementary Data S2). Most of these concerns originated from the randomization process and the selection of the reported results. Only six (19%) studies were prospectively registered and reported their planned analyses.^46,48,50,68–70^ Eight (26%) of the studies reported the details of the randomization process.^40,48,58,61,65,70,71,76^ Other common issues included a lack of power analysis and a clear description of the statistical analysis. Sixteen (52%) measured the serum selenium concentration and found a significant increase in selenium-treated participants to around 100 μg/L, while the control group showed no changes. Only one study found no significant increase in the selenium-treated group^67^ (Table 1). The overall certainty of evidence was moderate (Supplementary Table S6 in Supplementary Data S2).

### Assessment of publication bias

Supplementary Figure S24 in Supplementary Data S2 depicts the funnel plots for detection of publication bias. Egger's regression tests and the rank correlation test did not show statistically significant funnel plot asymmetry for any outcomes, except for TPOAb, where the rank correlation indicated significant funnel plot asymmetry (*p* = 0.04) (Table 2 and Supplementary Fig. S24 in Supplementary Data S2). Results remained similar after restricting the analyses to cohorts within the funnel (*p* > 0.05) (Supplementary Fig. S24 in Supplementary Data S2).

## Discussion

This systematic review and meta-analysis showed a significant reduction in TSH levels following selenium supplementation in HT patients without THRT, and these effects disappeared when including patients receiving THRT. Furthermore, selenium supplementation exhibited favorable results by reducing TPOAb and MDA levels, with no statistically significant effect on fT4, fT3, T4, T3, TGAb, thyroid volume, and IL-2 and IL-10. Selenium supplementation was well tolerated, as evidenced by the absence of significant differences in adverse events between the selenium and placebo groups. Overall, the certainty of evidence was moderate.

Previously, three meta-analyses have investigated the effect of selenium on TSH levels in autoimmune thyroiditis patients.^13,15,21^ One of these meta-analyses included exclusively LT4-untreated patients,^13^ one included LT4-treated and LT4-untreated patients,^21^ and the other additionally included patients with Graves' disease and hyperthyroidism.^15^ Neither meta-analyses found a significant effect of selenium on TSH levels. However, two of the prior meta-analyses were constrained by statistical power, including only five and eight trials, respectively.^13,15^ Furthermore, they exhibited considerable and unexplained heterogeneity (*I*^2^ = 94%, 76%, and 55%, respectively) and lacked subgroup analyses. Against this background, our meta-analysis adds to the existing knowledge in this field by demonstrating an effect of selenium supplementation on lowering TSH levels exclusively in HT patients without THRT. Moreover, the quality of evidence assessed in our meta-analysis was moderate, which is an improvement from the quality of evidence previously assessed (i.e., low to very low).^13^

Our study reaffirmed the results of six prior meta-analyses reporting an effect of selenium in reducing TPOAb levels.^14,15,17–19,21^ Of these, two meta-analyses consistently reported significant effects at 3, 6, and 12 months,^14,21^ two meta-analyses detected a significant effect solely at 6 months, not at 3 months,^17,19^ and the remaining two meta-analyses did not differentiate between time points.^15,18^ Against this background, our meta-analysis showed a consistent reduction in TPOAb levels, with significant reductions observed at 3–4 months and after at least 6 months of selenium supplementation. The inclusion of 31 cohorts enhanced statistical power compared with the previous meta-analyses, which included a maximum of nine cohorts.^14,15,17–19^ This expanded data set enabled us to address the substantial heterogeneity observed in both our analyses (*I*^2^ = 90%) and earlier meta-analyses (*I*^2^ = 99%,^15^ 94%,^19^ 67–95%,^17^ 23–97%,^14^ 63%,^18^ 95%^21^) through extensive subgroup and meta-regression analyses.

Our study provides a notable advance over previous systematic reviews and meta-analyses that were limited in scope and focused primarily on a restricted set of outcomes, such as thyroid function parameters (TSH, fT4, fT3),^13,15^ thyroid antibodies (TPOAb, TGAb),^14,15,17,19^ and, in two cases, mood, with limited cohort inclusion.^17,18^ We expanded the scope to encompass a broader range of thyroid function parameters, including T4 and T3, and ultrasound findings such as echogenicity and thyroid volume. We also included immune markers, revealing a significant reduction in MDA levels, a marker of oxidative stress.^78^ Importantly, our evidence indicated the safety of selenium supplementation at doses ranging from 80 to 400 μg per day for up to 12 months by showing comparable adverse events between the selenium-treated and control groups, filling a notable gap where such safety analyses were lacking. Adverse effects associated with adequate selenium supplementation are rare in the literature.

However, excess intake of selenium supplementation can be detrimental, with symptoms of acute toxicity (e.g., gastrointestinal and neurological manifestations) typically occurring at doses of 300–400 μg/day.^8,9^ The European Food Safety Authority (EFSA) has set the tolerable upper intake level for selenium at 255 μg/day.^79^ One clinical trial raised concerns that prolonged selenium supplementation of 200 μg/day could increase the incidence of type 2 diabetes.^80^ However, it should be noted that the overall study population was not initially selenium deficient, the supplementation duration was extended (7.7 years), and diabetes was a secondary outcome, making interpretation difficult. Furthermore, it has been recommended that people with serum or plasma selenium concentration of at least 122 μg/L should not supplement selenium as this may increase the risk of adverse events, including cancer and type 2 diabetes.^7,81^

Our detailed subgroup analyses for TSH, fT4, TPOAb, and TGAb identified patient groups and intervention types associated with the most robust results and explored factors contributing to heterogeneity. When we restricted our analysis to adult participants (≥18 years), selenium supplementation led to a reduction in TSH and TPOAb levels and an increase in fT4 levels. In contrast, results remained insignificant in minors (<18 years). Notably, only four of the included cohorts investigated minors, suggesting that the lack of a significant effect may be due to insufficient statistical power. We observed consistently stronger or similar results after limiting the analysis to cohorts with the selenium compound selenomethionine. Selenomethionine, an organic form of selenium, is the most commonly used form for dietary supplementation. Although inorganic selenium may have a greater ability inserted into GPX, organic forms dominate in their capability to be stored and integrated into body proteins.^82^

Subgroup analysis based on initial selenium status did not reveal clear trends. A possible explanation may be that data on selenium-sufficient cohorts were scarce. Most studies were conducted in populations most at risk for selenium deficiency, that is, the European and Chinese women.^12,83^ Only half of the studies reported selenium levels at baseline, of which 89% of the cohorts were selenium deficient. However, based on epidemiological data from China, Wu et al. postulated that selenium deficiency is a modifiable risk factor for HT.^12^ As deficiencies are expected to increase further with the rising popularity of plant-based diets, particularly among women,^84,85^ the recommended daily intake of ∼55–70 μg/day should be ensured, with higher intakes required during pregnancy and lactation.^9^ The female preponderance in our systematic review may be partly explained by the increased incidence and prevalence of HT in women.

However, the underrepresentation of male patients may lead to gender disparities in the management of HT. None of the studies analyzed the data by sex, highlighting the need for further investigation into potential sex differences. As exposure to sex hormones may influence autoimmunity, future investigations must also account for menopausal status (including the use of THRT).^86^ The high heterogeneity of results on TPOAb and TSH levels described in previous and in our current meta-analysis^13–15,17,19^ was reduced when we restricted our results to cohorts with severe selenium deficiency and to female cohorts only. This highlights the importance of adjusting for sex and selenium status of participants in future research.

Notably, no significant effects on TSH were observed when including participants receiving THRT. TSH levels may be difficult to interpret during LT4 substitution, as they primarily depend on replacement doses, and none of the included studies described a correction or washout procedure before laboratory quantification. In contrast, the effect on TPOAb was more pronounced in our study when we focused on HT patients initially treated with LT4 who had hypothyroidism. However, LT4 treatment may not necessarily reflect a decrease in the autoimmune response, but rather a normalization of thyroid function.^87^ Addressing the issue of LT4 dose adjustment may enhance the robustness of future trials. No significant increases in the thyroid hormones fT4 and fT3 were observed. This could be due to the fact that participants without THRT were mostly euthyroid or subclinically hypothyroid, that is, fT4 and fT3 levels were in the normal range (∼2–7 and 10–24 pmol/L, respectively).

HT is a complex autoimmune disease with pathophysiological mechanisms involving a feedback loop of immune system cells and cytokines. T cells are activated, leading to the production of inflammatory cytokines (e.g., IFN-γ and TNF-α) and the release of chemokines (e.g., CXCL-10) by thyrocytes, which further amplify the inflammatory response and attract additional T cells. This promotes the production of antibodies against thyroid-specific proteins (e.g., TPOAb, TGAb, TRAb), thyroid damage, and further development of hypothyroidism.^1,2,6^ In patients with HT, selenium supplementation can reduce TPOAb and TSH concentrations through several mechanisms, which can be related to the antioxidative and anti-inflammatory role of selenoproteins. Most selenoproteins are expressed in the thyroid gland, including the iodothyronine deiodinases, GPX, and selenoprotein S. These proteins perform essential functions such as thyroid hormone metabolism and activation, protection against oxidative damage, and regulation of the inflammatory response.^11,88^

Selenoenzymes, such as GPX, play an antioxidative role by reducing the formation of free radicals and hydrogen peroxides.^46^ Selenoenzymes also play a beneficial role in the immunoregulatory process, involving T cell activity and cytokine production.^70^ In line, several studies included in our systematic review showed that selenium supplementation increased GPX activity,^46,70,76^ decreased inflammatory and oxidative activity (IL-2, MDA, IFN-γ, and TNF-α),^45,46,52,71,72,74^ and increased antioxidant activity (IL-10, SOD, and TAC).^71,72^ Of note, there may be interindividual differences in the extent to which TPOAb concentrations change in response to selenium supplementation.^46^ Polymorphisms within selenoprotein genes impact not only their structure and function, but also individual responses to selenium intake. Consequently, some individuals may exhibit modulated inflammatory responses,^89^ endoplasmic reticulum stress in the brain,^90^ susceptibility to selenium deficiency, and specific response to selenium supplementation.^46^

This review has identified several limitations of the available evidence on the effects of selenium supplementation on HT. While measurements of TSH, fT4, and fT3 are more harmonized, the included studies used different assays to assess TPOAb and TGAb to measure distinct subgroups of antibodies with varying affinities.^91^ To address this issue, we synthesized the results of the studies using the SMD instead of the mean difference, which is recommended when varying scales and assays were used in the included studies. However, only the effect size can be interpreted and not its clinical significance.^22^ In addition, serum selenium concentrations can vary substantially depending on the analytical technique. Selenium status is typically assessed through the quantification of extracellular selenium levels in plasma or serum, reflecting the overall selenium intake within the past several days.^7,92^

In contrast to selenium in blood, intracellular selenium levels in erythrocytes, measured via inductively coupled plasma mass spectrometry, remain unaffected by the inflammatory response. This implies potential advantages, but standardized assessment is lacking.^92^ The general lack of harmonization may be a major cause of the large heterogeneity in the meta-analyses.

Another limitation of the studies included in this systematic review is the lack of information on the dietary habits of the participants. Selenium is present in various animal and plant food sources, and other dietary components, such as iodine or iron, can affect thyroid function and autoimmunity.^7,93^ Future studies should therefore report and account for baseline selenium, iodine, and iron status when analyzing the results. The same applies to the compliance, which was often not reported. Methods such as pill counts or evaluations of serum selenium levels should be used to assess compliance. In addition, the dose and regimen of THRT were not considered, which could significantly affect thyroid hormone levels, especially if THRT was taken immediately before the study assessments.

Finally, all included studies raised at least some concerns about the risk of bias according to the RoB 2 assessment. The methodology, for example, the randomization process and the selection of reported outcomes, was mostly not explained in detail, which should be improved in further studies. However, the leave-one-out analysis identified very few outlier cohorts, and exclusion produced similar results, leading to robust findings that were confirmed by the GRADE assessment.

## Conclusion and Implications for Practice, Policy, and Future Research

Our systematic review and meta-analysis provide valuable insights for clinical practice, policy, and future research.

Clinically, selenium supplementation showed promise in reducing TSH levels, especially in euthyroid and subclinically hypothyroid individuals without THRT. Moreover, selenium supplementation appears to have a beneficial effect in lowering TPOAb levels, although the clinical relevance of lowering TPOAb levels warrants further investigation.^94^ Selenium doses above 100 μg/day may be most potent.

In terms of policy implications, standardizing quantification techniques for thyroid hormones and antibodies is crucial for accurate and comparable measurements.

We recommend that future studies include selenium level assessment during the study, stratify data by sex, selenium status, and thyroid status, and provide clear reference values for all parameters. High-quality studies with larger sample sizes and detailed reporting according to Consolidated Standards of Reporting Trials guidelines^95^ are essential to confirm and fully understand selenium's role in HT. Research should also include children, adolescents, and pregnant women, and investigate the long-term effects of selenium supplementation on hypothyroidism development from euthyroidism.

In conclusion, our study suggests that selenium supplementation is safe and holds potential as a disease-modifying factor for HT-associated hypothyroidism. Further research is needed to confirm its efficacy, fully understand its mechanism of action, and elucidate its cost-effectiveness.

## Supplementary Material

Supplemental data

Supplemental data

## Appendix A1

### MEDLINE (Ovid)

Ovid MEDLINE(R) ALL <1946 to November 14, 2023>

1 thyroiditis, autoimmune/or hashimoto disease/11121

2 (hashimoto* or “struma lymphomatosa” or “lymphadenoid goiter*” or “lymphadenoid goitre*” or AITD or ((Autoimmun* or “auto immuni*”) adj4 (thyroid* or disease*)) or “chronic lymphocytic thyroid*” or “lymphomatous thyroid*”).ti,ab,kf. 116730

3 Selenium/or exp selenium compounds/or selenium-binding proteins/or exp selenoproteins/or Selenium Radioisotopes/or Methionine Sulfoxide Reductases/or exp Organoselenium Compounds/52556

4 (Selenium* or selenoprotein* or “seleno protein*” or “selenoamino acid*” or Selenomethioni* or selenolanthioni* or “Sodium Selenite*” or “Sodium selenate*” or “sodium selenide*” or “novamed selen*” or “radioactive selenium*” or “radio active selenium*” or selenocysteine* or selenocystine* or Organoselenium* or “Selenic Acid*” or “Selenious Acid*” or radioselenium* or “radio selenium*” or radioselenomethioni* or “radio selenomethioni*” or “Se supplement*” or “Se met” or “Methionine Sulfoxide Reductase*” or 70248-65-6 or 7782-49-2 or h6241uj22b or 13410-01-0 or 10236-58-5 or 1464-42-2).ti,ab,kf,nm,rn. 50309

5 1 or 2 119523

6 3 or 4 67425

7 5 and 6 394

8 randomized controlled trial.pt. 603048

9 controlled clinical trial.pt. 95450

10 randomi?ed.ab. 744952

11 placebo.ab. 243053

12 drug therapy.fs. 2638606

13 randomly.ab. 420679

14 trial.ab. 672120

15 groups.ab. 2594981

16 8 or 9 or 10 or 11 or 12 or 13 or 14 or 15 5822138

17 exp animals/not humans.sh. 5169565

18 16 not 17 5088400

19 7 and 18 171

### Embase (Elsevier)

Embase Session Results (November 14, 2023)

No. Query Results

#11 #9 AND #10 197

#10 random* OR factorial* OR crossover* OR (cross NEXT/1 over*) OR placebo* OR (doubl* AND blind*) OR (singl* AND blind*) OR assign* OR allocat* OR volunteer* OR ‘crossover procedure’/exp OR ‘double blind procedure’/exp OR ‘randomized controlled trial’/exp OR ‘single blind procedure’/exp 3265391

#9 #7 NOT #8 790

#8 [animals]/lim NOT [humans]/lim 6416725

#7 #5 AND #6 826

#6 #3 OR #4 74753

#5 #1 OR #2 208028

#4 selenium*:ti,ab,kw,ms,rn,tn OR selenoprotein*:ti,ab,kw,ms,rn,tn OR ‘seleno protein*’:ti,ab,kw,ms,rn,tn OR ‘selenoamino acid*’:ti,ab,kw,ms,rn,tn OR selenomethioni*:ti,ab,kw,ms,rn,tn OR selenolanthioni*:ti,ab,kw,ms,rn,tn OR ‘sodium selenite*’:ti,ab,kw,ms,rn,tn OR ‘sodium selenate*’:ti,ab,kw,ms,rn,tn OR ‘sodium selenide*’:ti,ab,kw,ms,rn,tn OR ‘novamed selen*’:ti,ab,kw,ms,rn,tn OR ’radioactive selenium*’:ti,ab,kw,ms,rn,tn OR ‘radio active selenium*’:ti,ab,kw,ms,rn,tn OR selenocystine*:ti,ab,kw,ms,rn,tn OR selenocysteine*:ti,ab,kw,ms,rn,tn OR organoselenium*:ti,ab,kw,ms,rn,tn OR ‘selenic acid*’:ti,ab,kw,ms,rn,tn OR ‘selenious acid*’:ti,ab,kw,ms,rn,tn OR radioselenium*:ti,ab,kw,ms,rn,tn OR ‘radio selenium*’:ti,ab,kw,ms,rn,tn OR radioselenomethioni*:ti,ab,kw,ms,rn,tn OR ‘radio selenomethioni*’:ti,ab,kw,ms,rn,tn OR ‘se supplement*’:ti,ab,kw,ms,rn,tn OR ‘se met’:ti,ab,kw,ms,rn,tn OR ‘methionine sulfoxide reductase*’:ti,ab,kw,ms,rn,tn OR ‘70248 65 6’:ti,ab,kw,ms,rn,tn OR ‘7782 49 2’:ti,ab,kw,ms,rn,tn OR h6241uj22b:ti,ab,kw,ms,rn,tn OR ‘13410 01 0’:ti,ab,kw,ms,rn,tn OR ‘10236 58 5’:ti,ab,kw,ms,rn,tn OR ‘1464 42 2’:ti,ab,kw,ms,rn,tn 67476

#3 ‘selenium’/de OR ‘selenium intake’/de OR ‘selenomethionine se 75’/de OR ‘selenoamino acid’/exp OR ‘selenium binding protein’/de OR ‘selenium derivative’/de OR ‘selenoprotein’/de OR ‘methionine sulfoxide reductase’/de OR ‘organoselenium derivative’/exp 63609

#2 hashimoto*:ti,ab,kw OR ‘struma lymphomatosa’:ti,ab,kw OR ‘lymphadenoid goiter*’:ti,ab,kw OR ‘lymphadenoid goitre*’:ti,ab,kw OR aitd:ti,ab,kw OR (((autoimmun* OR ‘auto immuni*’) NEAR/4 (thyroid* OR disease*)):ti,ab,kw) OR ‘chronic lymphocytic thyroid*’:ti,ab,kw OR ‘lymphomatous thyroid*’:ti,ab,kw 177536

#1 ‘autoimmune thyroiditis’/de OR ‘experimental autoimmune thyroiditis’/exp OR ‘hashimoto disease’/exp 52802

### Cochrane Library (Wiley) CENTRAL

Advanced Search via Search Manager (3 reviews, 79 trials) Only trials were exported.

ID Search Hits

#1 [mh ^“thyroiditis, autoimmune”] OR [mh “hashimoto disease”] 178

#2 (hashimoto* OR “struma lymphomatosa” OR (lymphadenoid NEXT goiter*) OR (lymphadenoid NEXT goitre*) OR AITD OR ((Autoimmun* OR (auto NEXT immuni*)) NEAR/4 (thyroid* OR disease*)) OR (chronic NEXT lymphocytic NEXT thyroid*) OR (lymphomatous NEXT thyroid*)):ti,ab,kw 4307

#3 [mh selenium] OR [mh “selenium radioisotopes”] OR [mh “selenium compounds”] OR [mh selenoproteins] OR [mh “selenium binding proteins”] OR [mh “organoselenium compounds”] 1362

#4 (Selenium* OR selenoprotein* OR (seleno NEXT protein*) OR (selenoamino NEXT acid*) OR Selenomethioni* OR Selenolanthioni* OR (Sodium NEXT Selenite*) OR (Sodium NEXT selenate*) OR (Sodium NEXT selenide*) OR (novamed NEXT selen*) OR (radioactive NEXT selenium*) OR (radio NEXT active NEXT selenium*) OR selenocystine* OR selenocysteine* OR Organoselenium* OR (Selenic NEXT Acid*) OR (Selenious NEXT Acid*) OR radioselenium* OR (radio NEXT selenium*) OR radioselenomethioni* OR (radio NEXT selenomethioni*) OR (Se NEXT supplement*) OR “Se met” OR (Methionine NEXT Sulfoxide NEXT Reductase*) OR “70248 65 6” OR “7782 49 2” OR “h6241uj22b” OR “13410 01 0” OR “10236 58 5” OR “1464 42 2”):ti,ab,kw 2716

#5 #1 OR #2 4307

#6 #3 OR #4 3083

#7 #5 AND #6 87

### CINAHL with Full Text (Ebsco)

Advanced Search; Search Mode: Boolean Phrase

S1 MH “Thyroiditis, Autoimmune” OR (hashimoto* OR “struma lymphomatosa” OR “lymphadenoid goiter*” OR “lymphadenoid goitre*” OR AITD OR ((Autoimmun* OR “auto immuni*”) N4 (thyroid* OR disease*)) OR “chronic lymphocytic thyroid*” OR “lymphomatous thyroid*”) 25,018

S2 (MH “Selenium” OR MH “Selenium Compounds” OR Selenium* OR selenoprotein* OR “seleno protein*” OR “selenoamino acid*” OR Selenomethioni* OR selenolanthioni* OR “Sodium Selenite*” OR “Sodium selenate*” OR “sodium selenide*” OR “novamed selen*” OR “radioactive selenium*” OR “radio active selenium*” OR selenocysteine* OR selenocystine* OR Organoselenium* OR “Selenic Acid*” OR “Selenious Acid*” OR radioselenium* OR “radio selenium*” OR radioselenomethioni* OR “radio selenomethioni*” OR “Se supplement*” OR “Se met” OR “Methionine Sulfoxide Reductase*” OR 70248-65-6 OR 7782-49-2 OR h6241uj22b OR 13410-01-0 OR 10236-58-5 OR 1464-42-2) 4,310

S3 S1 AND S2 101

S4 (MH “Animals” NOT MH “Human”) 88,551

S5 S3 NOT S4 99

S6 (MH “Clinical Trials+”) OR (PT (Clinical trial)) OR (MH “Random Assignment”) OR (MH “Quantitative Studies”) OR (TX ((clini* N1 trial*) OR (singl* N1 blind*) OR (singl* N1 mask*) OR (doubl* N1 blind*) OR (doubl* N1 mask*) OR (tripl* N1 blind*) OR (tripl* N1 mask*) OR (random* N1 allocat*) OR placebo* OR ((waitlist* OR (wait* and list*)) and (control* OR group)) OR “treatment as usual” OR tau OR (control* N3 (trial* OR study OR studies OR group*)) OR randomized OR randomised)) 1,953,780

S7 S5 AND S6 38

### Science Citation Index Expanded & Emerging Sources Citation Index (Web of Science)

#### Advanced search

1 TS = (hashimoto* OR “struma lymphomatosa” OR “lymphadenoid goiter*” OR “lymphadenoid goitre*” OR AITD OR ((Autoimmun* OR “auto immuni*”) NEAR/4 (thyroid* OR disease*)) OR “chronic lymphocytic thyroid*” OR “lymphomatous thyroid*”) 122261

2 TS = (Selenium* OR selenoprotein* OR “seleno protein*” OR “selenoamino acid*” OR Selenomethioni* OR selenolanthioni* OR “Sodium Selenite*” OR “Sodium selenate*” OR “sodium selenide*” OR “novamed selen*” OR “radioactive selenium*” OR “radio active selenium*” OR selenocysteine* OR selenocystine* OR Organoselenium* OR “Selenic Acid*” OR “Selenious Acid*” OR radioselenium* OR “radio selenium*” OR radioselenomethioni* OR “radio selenomethioni*” OR “Se supplement*” OR “Se met” OR “Methionine Sulfoxide Reductase*” OR 70248-65-6 OR 7782-49-2 OR h6241uj22b OR 13410-01-0 OR 10236-58-5 OR 1464-42-2) 90224

3 TS = (random* OR control* OR study OR trial OR compar* OR group OR groups OR therapy OR treatment OR intervention) 36952749

4 #3 AND #2 AND #1 419

### Google Scholar via *Publish or Perish* Software Program

Search Direct: Maximum number of results: 200 (most relevant)

hashimoto|“chronic lymphocytic thyroiditis” Selenium|“Sodium Selenite”|“Sodium selenate”|“sodium selenide” RCT|“randomized controlled trial”
