# Supplementary material for: Selenium Supplementation in Patients with Hashimoto Thyroiditis: A Systematic Review and Meta-Analysis of Randomized Clinical Trials
Source: Thyroid. 2024 Mar 13;34(3):295–313. doi: 10.1089/thy.2023.0556 (PMC10951571; doi:10.1089/thy.2023.0556)
Supplement: Supplemental data [file Suppl_DataS1.docx]

# Materials and Methods

## Search Strategy

We systematically searched the five electronic databases for publications:

- MEDLINE via Ovid
- Embase via Elsevier
- CINHAL via Ebsco
- Web of Science
- Google Scholar

CENTRAL Cochrane Library trial registry for ongoing and terminated trials.

A medical information specialist designed the search strategy and developed the search term using controlled vocabulary (e.g., MeSH, EMTREE) and free text terms for the following elements: HT, selenium and related terms, and the study design (randomized controlled trials [RCTs]). The specialist translated the search term for each information source accordingly. We incorporated and adapted study design search filters and search blocks from the Cochrane Handbook RCT search filter (sensitivity max. version 2008) for MEDLINE (Ovid) section 4.4.7^1^ and the RCT search blocks by Ket et al. (2020) available from https://blocks.bmi-online.nl/catalog/296.

Search results were exported into the citation management program EndNote (version 20) for deduplication, using EndNote’s automation features and methods described by Bramer et al.^2^ Subsequently, we imported the RIS file to Rayyan software^3^ for title/abstract and full-text screening.

## Study Eligibility and Data Extraction

We excluded observational studies, conference abstracts and proceedings, case reports, editorials, letters, and notes.

We retrieved full-text articles of relevant studies, and the two authors assessed them separately. Publications in foreign languages were translated by a colleague^4^ or using the DeepL software^5,6^.

We extracted the following data with a standardized, predesigned coding sheet:

- Report: title, authors, year, publication journal, Digital Object Identifier (DOI);
- Study: design, intention-to-treat or per-protocol analysis, trial registration, funding sources, country of execution;
- Participants: group size, BMI range, age, sex distribution, thyroid functional status, co-morbidities, HT diagnosis criteria, hypothyroidism treatment, blood levels of selenium;
- Intervention: intervention product, dose, intervention duration;
- Comparator: placebo, no intervention
- Outcome: outcomes results at baseline and end of the intervention, assessment assays.

Only cohorts receiving the same intervention throughout the entire study were considered for analysis.

Results from graphs that were inconsistent with the results presented in the original manuscript (e.g., significant reduction in our analysis despite lack of significance in the original publication) were excluded.

## Data Synthesis and Statistical Analysis

Selenium status was arbitrarily categorized based on levels of selenium in plasma or whole blood as severely selenium deficient (<80 µg/L; below optimal GPX acitivity^7^), mildly selenium deficient (80 to <120 µg/L; below maximal SELENP concentration^7,8^), or selenium sufficient (≥120 µg/L; selenium supplementation with serum or plasma selenium levels of below 122 µg/L associated with various health benefits with no extra risk^9^).

All units were standardized prior to analysis. We converted median and interquartile ranges to mean and standard deviation according to Wan et al.^10^ and estimated missing standard deviations of the outcome TPOAb based on the standard deviation of a study with similar data.

We statistically analyzed the results as standardized mean differences (SMD) using the random-effects model with the DerSimonian-Laird estimator estimator, accounting for systematic variations in effect sizes across studies.^11,12^ The studies were weighted using the inverse-variance method. We constructed forest plots and estimated heterogeneity between studies using the Q statistics according to Cochrane and the *I^2^* statistic.^13,14^ We considered *I^2^* <50% low*, I^2^* between 50% and <70% as moderate, and *I^2^* ≥75% as high.

The leave-one-out diagnostics identified outlier cohorts based on the externally standardized residual (rstudent), DFFITS value (dffits), Cook's distance (cook.d), covariance ratio (cov.r), leave-one-out amount of (residual) heterogeneity (tau2.del), leave-one-out test statistic of the test for (residual) heterogeneity (QE.del), and DFBETAS value (hat, weight).^15^

## Quality Assessment

The revised Cochrane risk of bias tool for randomized clinical trials (RoB 2)^16^ evaluates the risk of bias in relation to the five domains of randomization process, deviation from intended intervention, missing outcome data, outcome measurement, and selection of reported results and categorized them into low, some concerns, and high. Overall risk of bias was determined based on the highest rated domain.

## GRADEing of evidence

The Grading of Recommendations Assessment, Development and Evaluation (GRADE) method^17^ rates evidence as high, moderate, low, or very low based on two factors; effect size and the quality of the evidence, which considers study design, risk of bias, consistency, directness, precision, and other aspects.

## References

1. Higgins JP, Thomas J, Chandler J, et al. Cochrane handbook for systematic reviews of interventions. John Wiley & Sons: 2019.

2. Bramer WM, Giustini D, de Jonge GB, et al. De-duplication of database search results for systematic reviews in EndNote. Journal of the Medical Library Association: JMLA 2016;104(3):240

3. Ouzzani M, Hammady H, Fedorowicz Z, et al. Rayyan—a web and mobile app for systematic reviews. Systematic reviews 2016;5(210):1-10, doi:10.1186/s13643-016-0384-4

4. Zhu L, Bai X, Teng W, et al. Effects of selenium supplementation on antibodies of autoimmune thyroiditis. Zhonghua Yi Xue Za Zhi 2012;92(32):2256-2260

5. Shabalina EA, Fadeyev VV. Effects of selenium in patients with autoimmune thyroiditis. Clinical and experimental thyroidology 2019;15(2):44-54

6. Balázs C. The effect of selenium therapy on autoimmune thyroiditis. Orv Hetil 2008;149(26):1227-1232, doi:10.1556/oh.2008.28408

7. Kipp AP, Strohm D, Brigelius-Flohé R, et al. Revised reference values for selenium intake. Journal of Trace Elements in Medicine and Biology 2015;Oct(32):195-9, doi:10.1016/j.jtemb.2015.07.005

8. Hurst R, Armah CN, Dainty JR, et al. Establishing optimal selenium status: results of a randomized, double-blind, placebo-controlled trial. Am J Clin Nutr 2010;91(4):923-31, doi:10.3945/ajcn.2009.28169

9. Rayman MP. Selenium and human health. Lancet 2012;379(9822):1256-1268, doi:10.1016/s0140-6736(11)61452-9

10. Wan X, Wang W, Liu J, et al. Estimating the sample mean and standard deviation from the sample size, median, range and/or interquartile range. BMC Med Res Methodol 2014;19(14):1-13, doi:10.1186/1471-2288-14-135

11. Raudenbush SW. Analyzing effect sizes: Random-effects models. Russell Sage Foundation: 2009.

12. DerSimonian R, Laird N. Meta-analysis in clinical trials. Controlled Clinical Trials 1986;7(3):177-188, doi:10.1016/0197-2456(86)90046-2

13. Higgins JP, Thompson SG. Quantifying heterogeneity in a meta-analysis. Statistics in Medicine 2002;21(11):1539-58, doi:10.1002/sim.1186

14. Higgins JP, Thompson SG, Deeks JJ, et al. Measuring inconsistency in meta-analyses. Bmj 2003;327(7414):557-560, doi:10.1136/bmj.327.7414.557

15. Viechtbauer W, Cheung MW. Outlier and influence diagnostics for meta-analysis. Research Synthesis Methods 2010;1(2):112-25, doi:10.1002/jrsm.11

16. Sterne JA, Savović J, Page MJ, et al. RoB 2: a revised tool for assessing risk of bias in randomised trials. Bmj 2019;28(366):l4898, doi:10.1136/bmj.l4898

17. Schünemann H BJ, Guyatt G, Oxman A, editors. GRADE handbook for grading quality of evidence and strength of recommendations. Updated October 2013. The GRADE Working Group, 2013. 2013. Available from: guidelinedevelopment.org/handbook. [Last Accessed; 18.05.2022].
