# Supplementary material for: Selenium Supplementation in Patients with Hashimoto Thyroiditis: A Systematic Review and Meta-Analysis of Randomized Clinical Trials
Source: Thyroid. 2024 Mar 13;34(3):295–313. doi: 10.1089/thy.2023.0556 (PMC10951571; doi:10.1089/thy.2023.0556)
Supplement: Supplemental data [file Suppl_DataS2.docx]

**Table of Figures**

**Figure S1.** **Effect of selenium supplementation on thyroid-stimulating hormones [TSH, mIU/L] in Hashimoto thyroiditis with and without thyroid hormone replacement therapy (n = 2063).**. 14

**Figure S2.** **Effect of selenium supplementation on thyroid-stimulating hormones [TSH, mIU/L], stratified by thyroid status at study start, in patients with Hashimoto thyroiditis with thyroid hormone replacement therapy (n = 794).**. 15

**Figure S3. Effect of selenium supplementation on free thyroxine [fT4, pmol/L] in Hashimoto thyroiditis with and without thyroid hormone replacement therapy (n = 1664).** 16

**Figure S4. Effect of selenium supplementation on free thyroxine [fT4, pmol/L], stratified by thyroid status, in Hashimoto thyroiditis without thyroid hormone replacement therapy (n = 623).** 17

**Figure S5. Effect of selenium supplementation on free thyroxine [fT4, pmol/L], stratified by thyroid status, in Hashimoto thyroiditis with thyroid hormone replacement therapy (n = 842).** 18

**Figure S6. Effect of selenium supplementation on free triiodothyronine [fT3, pmol/L] in patients with Hashimoto thyroiditis with and without thyroid hormone replacement therapy (n = 658).** 19

**Figure S7.** **Effect of selenium supplementation on free triiodothyronine [fT3, pmol/L] in patients (all euthyroid) with Hashimoto thyroiditis without thyroid hormone replacement therapy (n = 239).** 20

**Figure S8. Effect of selenium supplementation on free triiodothyronine [fT3, pmol/L], stratified by thyroid status at study start, in patients with Hashimoto thyroiditis with thyroid hormone replacement therapy (n = 368).** 21

**Figure S9. Effect of selenium supplementation on total thyroxine [T4, pg/mL] in Hashimoto thyroiditis (n = 187).** 22

**Figure S10. Effect of selenium supplementation on total triiodothyronine [T3, nmol/L] in Hashimoto thyroiditis (n = 252).** 23

**Figure S11. Effect of selenium supplementation started during pregnancy on thyroid peroxidase antibodies [TPOAb, IU/mL] in pregnant TPOAb-positive women at different time points.** 24

**Figure S12. Effect of selenium supplementation on thyroglobulin antibodies [TGAb, IU/mL] in Hashimoto thyroiditis (n = 1283).** 25

**Figure S13.** **Effect of selenium supplementation on thyroid volume [mL] in Hashimoto thyroiditis (n = 182).** 26

**Figure S14.** **Effect of selenium supplementation on interleukin-2 [IL-2; pg/mL] in Hashimoto thyroiditis (n = 189).** 27

**Figure S15.** **Effect of selenium supplementation on interleukin-10 [IL-10; pg/mL] in Hashimoto thyroiditis (n = 189).** 28

**Figure S16. Effect of selenium supplementation on malondialdehyde [MDA; nmol/mL] in Hashimoto thyroiditis (n = 248).** 29

**Figure S17. Effect of selenium supplementation on adverse events (log odds ratio) in Hashimoto thyroiditis (n = 1339).** 30

**Figure S18. (1) Stratified forest plots and (2) scatterplots of the meta-regression analysis of SMD of thyroid-stimulating hormone (TSH) levels [mIU/L] of the moderators (A) dose, (B) duration, (C) selenium status, (D) sex distribution, (E) thyroid status, (F) age, (G) thyroid hormone replacement therapy, (H) selenium compound, and (I) blinding of the study.** 33

**Figure S19. (1) Stratified forest plots and (2) scatterplots of the meta-regression analysis of SMD of free thyroxine (fT4) levels [pmol/L] of the moderators (A) dose, (B) duration, (C) selenium status, (D) sex distribution, (E) thyroid status, (F) age, (G) thyroid hormone replacement therapy, (H) selenium compound, and (I) blinding of the study.** 36

**Figure S20. (1) Stratified forest plots and (2) scatterplots of the meta-regression analysis of SMD of free triiodothyronine (fT3) levels [pmol/L] of the moderators (A) dose, (B) duration, (C) selenium status, (D) sex distribution, (E) thyroid status, (F) age, (G) thyroid hormone replacement therapy, (H) selenium compound, and (I) blinding of the study.** 38

**Figure S21. (1) Stratified forest plots and (2) scatterplots of the meta-regression analysis of SMD of thyroid peroxidase antibodies (TPOAb) levels [IU/mL] of the moderators (A) dose, (B) duration, (C) selenium status, (D) sex distribution, (E) thyroid status, (F) age, (G) thyroid hormone replacement therapy, (H) selenium compound, and (I) blinding of the study.** 41

**Figure S22. (1) Stratified forest plots and (2) scatterplots of the meta-regression analysis of SMD of thyroglobulin antibodies (TGAb) levels [IU/mL] of the moderators (A) dose, (B) duration, (C) selenium status, (D) sex distribution, (E) thyroid status, (F) age, (G) thyroid hormone replacement therapy, (H) selenium compound, and (I) blinding of the study.** 44

**Figure S23. Outlier and influential case diagnostics using (1) visual inspection and (2) leave-one-out diagnostic for the outcomes (A) thyroid-stimulating hormone (TSH), (B) free thyroxine (fT4), (C) free triiodothyronine (fT3), (D) thyroid peroxidase antibodies (TPOAb), and (E) thyroglobulin antibodies (TGAb).** 47

**Figure S24. Funnel plots for detection of publication bias for the outcomes (A) thyroid-stimulating hormone (TSH), (B) free triiodothyronine (fT3), (C) free thyroxine (fT4), (D) thyroid peroxidase antibodies (TPOAb), (E) thyroglobulin antibodies (TGAb), and (F) adverse events.** 52

**Table S1.** Author, year, and title of the 26 excluded studies including reason for exclusion (duplicates, study population, study design, and publication type) with details.

| **Author, Year** | **Title** | **Details** |
| --- | --- | --- |
| **Duplicates (*n*= 13)** | | **Duplicate to  [Author, Year]** |
| Bonfig, 2010 ^1^ | Selenium supplementation does not decrease thyroid peroxidase antibody concentration in children and adolescents with autoimmune thyroiditis | Bonfig, 2010 ^2^ |
| Cenci, 2013 ^3^ | Selenomethionine supplementation in euthyroid patients with autoimmune thyroiditis: effects of two doses (80 or 160 mug) versus placebo | Pilli, 2015 ^4^ |
| Hu, 2021 ^5^ | Effect of selenium on thyroid autoimmunity and regulatory T cells in patients with Hashimoto's thyroiditis: A prospective randomized controlled trial | Hu, 2021 ^6^ |
| Krysiak, 2011 ^7^ | The effect of levothyroxine and selenomethionine on lymphocyte and monocyte cytokine release in women with Hashimoto's thyroiditis | Krysiak, 2012 ^8^ |
| Kyrgios, 2019 ^9^ | L-selenomethionine supplementation in children and adolescents with autoimmune thyroiditis: A randomized double-blind placebo-controlled clinical trial | Kyrgios, 2019 ^10^ |
| Kyrgios, 2016 ^11^ | L-selenomethionine supplementation in children and adolescents with autoimmune thyroiditis: Preliminary results of a randomized double-blinded placebo-controlled clinical trial | Kyrgios, 2019 ^10^ |
| Mahmoudi, 2021 ^12^ | Effect of Selenium-Enriched Yeast supplementation on serum thyroid-stimulating hormone and anti-thyroid peroxidase antibody levels in subclinical hypothyroidism: Randomized controlled trial | Mahmoudi, 2021 ^12^ |
| Negro, 2010 ^13^ | Postpartum thyroiditis | Negro, 2007 ^14^ |
| Pilli, 2015 ^15^ | IFNγ-Inducible chemokines decrease upon selenomethionine supplementation in women with euthyroid autoimmune thyroiditis: comparison between two doses of selenomethionine (80 or 160μg) versus placebo | Pilli, 2015 ^4^ |
| Pilli, 2014 ^16^ | IFN gamma-Inducible Chemokines Are Down-Modulated By Selenomethionine (Semet) Supplementation in Women with Euthyroid Chronic Autoimmune Thyroiditis (AIT): Comparison Between 2 Doses of Semet (80 mu g or 160 mu g) Versus Placebo | Pilli, 2015 ^4^ |
| Pilli, 2014 ^17^ | IFNγ-inducible chemokines are down-modulated by selenomethionine (SEMET) supplementation in women with euthyroid chronic autoimmune thyroiditis (AIT): Comparison between 2 doses of semet (80 μg or 160 μg) versus placebo | Pilli, 2015 ^4^ |
| Pilli, 2013 ^18^ | Effects of 2 doses (80 or 160 lg) of selenomethionine supplementation versus placebo in euthyroid female patients with autoimmune thyroiditis | Pilli, 2015 ^4^ |
| Wang, 2018 ^19^ | Decreased Thyroid Peroxidase Antibody Titer in Response to Selenium Supplementation in Autoimmune Thyroiditis and the Influence of a Selenoprotein P Gene Polymorphism: A Prospective, Multicenter Study in China | Wang, 2018 ^19^ |
| **Study population (*n* = 2)** | |  |
| Thomson, 2011 ^20^ | Minimal impact of excess iodate intake on thyroid hormones and selenium status in older New Zealanders | Healthy population |
| Kvicala, 2009 ^21^ | Effect of selenium supplementation on thyroid antibodies | Seniors without Hashimoto thyroiditis |
| **Study design (*n* = 10)** | |  |
| Berisha-Muharremi, 2023 ^22^ | Efficacy of Combined Photobiomodulation Therapy with Supplements versus Supplements alone in Restoring Thyroid Gland Homeostasis in Hashimoto Thyroiditis: A Clinical Feasibility Parallel Trial with 6-Months Follow-Up | No control group |
| Filipowicz, 2022 ^23^ | Selenium Status and Supplementation Effects in Pregnancy-A Study on Mother-Child Pairs from a Single-Center Cohort | No control group |
| Gartner, 2003 ^24^ | Selenium in the treatment of autoimmune thyroiditis | Follow-up study of Gärtner, 2002 ^25^ with changing intervention |
| Kryczyk-Koziol, 2022 ^26^ | Assessment of the Effect of Selenium Supplementation on Production of Selected Cytokines in Women with Hashimoto's Thyroiditis | No control group |
| Krysiak, 2023 ^27^ | Sexual Function and Depressive Symptoms in Young Women with Euthyroid Hashimoto's Thyroiditis Receiving Vitamin D, Selenomethionine and Myo-Inositol: A Pilot Study | Not randomized |
| Mazokopakis, 2007 ^28^ | Effects of 12 months treatment with L-selenomethionine on serum anti-TPO Levels in Patients with Hashimoto's thyroiditis | All patients received selenium in the first phase of the study |
| Neto, 2015 ^29^ | Effects of selenium supplementationonserum thyroid peroxidase antibody levels in patients with autoimmune thyroiditis | No control group |
| Nordio, 2013 ^30^ | Research Article Combined Treatment with Myo-Inositol and Selenium Ensures Euthyroidism in Subclinical Hypothyroidism Patients with Autoimmune Thyroiditis | No control group |
| Nordio, 2013 ^30^ | Combined treatment with Myo-inositol and selenium ensures euthyroidism in subclinical hypothyroidism patients with autoimmune thyroiditis | No control group |
| Sun, 2020 ^31^ | Effect of levothyroxine sodium combined with selenium supplement | Retrospective study, not randomized |
| **Publication type (*n* = 1)** | |  |
| Gartner, 2008 ^32^ | Effects of selenium supplementation on TPOAb and cytokines in acute autoimmune thyroiditis | Editorial letter, same study as Gärtner, 2002 ^25^ |

**Table S2. Author, year, and country of study and diagnostic criteria for Hashimoto thyroiditis.** The thresholds of the thyroid peroxidase antibodies (TPOAb) values used for diagnosis varied from having no reported threshold values^14,33-37^ to ≥5.6 IU/mL^38^, ≥16 IU/mL^39^, ≥34 IU/mL^6,12,40^, ≥35 IU/mL^41^, ≥40 IU/mL^2^, ≥60 IU/mL^10,42,43^, ≥100  IU/mL^4,8,44-48^, ≥150  IU/mL^49^, ≥250  IU/mL^50^, ≥300  IU/mL^19,51^, and ≥350 IU/mL^25,52^. Four studies (11%) did not define any diagnostic criteria^53-56^ or used uncommon units (i.e., %) for TPOAb^12,57^.

| **Author, Year** | **Country** | **Diagnostic criteria** |
| --- | --- | --- |
| Anastasilakis, 2012 ^33^ | Greece | 1. Increased levels of TPOAb and TGAb 2. Typical hypoechogenecity of the thyroid in high-resolution ultrasound scan |
| Balazs, 2009 ^56^ | Hungary | NA |
| Bhuyan, 2012 ^49^ | India | 1. TPOAb ≥150 IU/mL |
| Bonfig, 2010 ^2^ | Germany | 1. TPOAb and/or TGAb ≥40 IU/mL 2. Hypothyroidism with or without a goiter |
| Chakrabarti, 2016 ^58^ | India | 1. Low fT4 level with elevated TSH level (≥35 IU/mL) |
| De Farias, 2015 ^44^ | Brazil | 1. TPOAb >100 UI/mL 2. Thyroid hypoechogenicity on high-resolution ultrasound 3. Normal or elevated TSH and normal fT4 |
| Duntas, 2003 ^45^ | Greece | 1. TPOAb >100 U/L |
| Eskes, 2014 ^46^ | Netherlands | 1. TPOAb ≥100 kU/L 2. TSH 0.5-5.0 mU/L, fT4 10–23 pmol/L |
| Esposito, 2017 ^52^ | Italy | 1. TPOAb and TGAb >350 IU/mL 2. Thyroid parenchyma heterogeneity with reduced echogenicity 3. Normal TSH, fT3 and fT4 serum levels |
| Gärtner, 2002 ^25^ | Germany | 1. TPOAb and/or TGAb >350 IU/mL 2. Basal TSH levels 3. Typical hypoechogenicity of the thyroid in high resolution sonography |
| Hu, 2021 ^6^ | China | 1. TPOAb >34 IU/mL or/and TGAb >115 IU/mL 2. Diffuse thyroid disease and heterogeneous echogenicity on ultrasonography |
| Kachouei, 2018 ^55^ | Iran | NA |
| Karanikas, 2008 ^40^ | Austria | 1. TPOAb positive ≥34 IU/mL 2. Negativity for anti-TSH receptor antibodies 3. Thyroid ultrasound imaging suggestive for a chronic thyroiditis (typical hypoechogenicity) |
| Karimi, 2019 ^50^ | Iran | 1. TPOAb ≥250 IU/ml |
| Krysiak, 2012 ^8^ | Poland | 1) TPOAb >100 U/mlL  2) Reduced echogenicity of the thyroid parenchyma on thyroid ultrasonography |
| Kyrgios, 2019 ^10^ | Greece | 1. TPOAb and/or TGAb ≥60 IU/mL |
| Mahmoodianfard, 2015 ^54^ | Iran | NA |
| Mahmoudi, 2021 ^12^ | Iran | 1. TPOAb >34 mg/dL 2. Elevated basal TSH levels in two consecutive tests 3. Examination by expert endocrinologist; |
| Mantovani, 2019 ^38^ | Italy | 1. TGAb >4.11 IU/L and/or TPOAb >5.61 IU/L |
| Mao, 2016 ^34^ | UK | 1. TPOAb positive |
| Nacamulli, 2010 ^39^ | Italy | 1. TPOAb ≥16 IU/mL and TGAb ≥100 IU/mL   and/or  Characteristic thyroid ultrasound (US) pattern (scattered or widespread hypoechogenic areas with or without increased vascularization)   1. Normal or slightly elevated (<8 IU/L) serum TSH levels with normal fT4 |
| Negro, 2007 ^14^ | Italy | 1. TPOAb positive |
| Negro, 2016 ^35^ | Italy | 1. Presence of TPOAb 2. TSH 4.6-10.0 IU/L (MH) and TSH 10.1-20.0 IU/L (SH) |
| Pilli, 2015 ^4^ | Italy | 1. TPOAb and/or TGAb ≥100 U/ mL 2. Characteristic thyroid ultrasound pattern (scattered or widespread hypoechogenicity) |
| Pirola, 2016 ^42^ | Italy | 1. TPOAb ≥60 U/mL 2. Typical ultrasound features |
| Preda, 2017 ^41^ | Romania | 1. TPOAb >35 UI/mL 2. Normal thyroid function (TSH 0.4-4 μIU/mL) |
| Shabalina, 2019 ^47^ | Russia | 1. TPOAb >100 mU/L 2. TTH 0.4-10.0 mU/L |
| Sun, 2021 ^43^ | China | 1. TPOAb >60 IU/mL and TGAb >60 IU/mL 2. Color Doppler ultrasound showing diffuse thyroid lesions rather than thyroid nodules or other lesions 3. Thyroid fine needle aspiration and cytology consistent with the pathological manifestations of CLT.   The diagnosis was confirmed if 1)+2) or 2)+3) was met |
| Tian, 2020 ^36^ | China | 1. TPOAb and/or TGAb positive 2. Presence of parenchymal heterogeneity in high-resolution sonography |
| Turker, 2006 ^59^ | Turkey | 1. TPOAb >100 IU/mL and/or TGAb >188 IU/mL |
| Wang, 2018 ^19^ | China | 1. TPOAb >300 IU/mL 2. Thyroid hormone levels within the reference range 3. TSH levels could be above the upper normal level (subclinical hypothyroidism) |
| Wu, 2018 ^53^ | China | NA |
| Yu, 2017 ^57^ | China | 1. TPOAb and TGAb levels not less than 50% 2. Chinese thyroid disease diagnosis guidelines |
| Zhang, 2020 ^37^ | China | 1. TPOAb positive 2. Diagnosed Hashimoto’s thyroiditis by laboratory tests according to the diagnosis criteria of Hashimoto thyroiditis 3. Low fT3 & fT4 and high TSH |
| Zhu, 2012 ^51^ | China | 1. TPOAb >300 U/mL |

MH = Mild autoimmune hypothyroidism; SH = Severe autoimmune hypothyroidism; fT3 = Free triiodothyronine; fT4 = Free thyroxine; TGAb = Thyroglobulin antibodies; THRT = Thyroid hormone replacement therapy; TPOAb = Thyroid peroxidase antibodies; TSH = Thyroid-stimulating hormone; TTH = Total thyroid hormones.

**Table S3.** **Normal ranges for thyroid-stimulating hormone (TSH), free triiodothyronine (fT3), and free thyroxine (fT4) defined for each study and assays used for quantification.**

| **Author, Year** | **TSH [mIU/L]** | | | | **fT3 [pmol/L]** | | | | **fT4 [pmol/L]** | | | | |
| --- | --- | --- | --- | --- | --- | --- | --- | --- | --- | --- | --- | --- | --- |
|  | **Normal range** | | | **Assay** | **Normal range** | | | **Assay** | **Normal range** | | | **Assay** | |
| Anastasilakis, 2012 ^33^ | NA |  |  | CMIA, details missing (Abbott Diagnostics, Ireland) | NA |  |  | CMIA, details missing (Abbott Diagnostics, Ireland) | NA |  |  | CMIA, details missing (Abbott Diagnostics, Ireland) |  |
| Balazs, 2009 ^56^ | 0.27 | - | 4.2 | CL, Elecsys 2010 (Roche, Germany) | 1.45 | - | 3.48 | CL, Elecsys 2010 (Roche, Germany) | 0.71 | - | 1.85 | CL, Elecsys 2010 (Roche, Germany) |  |
| Bhuyan, 2012 ^49^ | NA |  |  | NA | NA |  |  | NA | NA |  |  | NA |  |
| Bonfig, 2010 ^2^ | 0.3 | - | 4.0 | EIA, ADVIA Centaur XP Immunoassay System (Siemens, Germany) | 4.0 | - | 7.7 | EIA, ADVIA Centaur XP Immunoassay System (Siemens, Germany) | 10.3 | - | 23.2 | EIA ADVIA Centaur XP Immunoassay System (Siemens, Germany |  |
| Chakrabarti, 2016 ^58^ | ≤4.0 |  |  | CL; details missing | NA |  |  | NA | ≥10.3 |  |  | CL |  |
| De Farias, 2015 ^44^ | NA |  |  | Fluorometry, AutoDELFIA  (Perkin Elmer, Finland) | NA |  |  | Fluorometry, AutoDELFIA  (Perkin Elmer, Finland) | NA |  |  | Fluorometry, AutoDELFIA  (Perkin Elmer, Finland) |  |
| Duntas, 2003 ^45^ | ≤4.0 |  |  | Details missing (Nichols, USA) | NA |  |  | NA | 9 | - | 25 | ICL, details missing (Nichols, USA) |  |
| Eskes, 2014 ^46^ | 0.5 | - | 5.0 | Fluorometry, AutoDELFIA  (Perkin Elmer, Finland) | NA |  |  | NA | 10 | - | 23 | Fluorometry, AutoDELFIA  (Perkin Elmer, Finland) |  |
| Esposito, 2017 ^52^ | NA |  |  | Fluorometry, AutoDELFIA  (Perkin Elmer, Finland) | NA |  |  | Fluorometry, AutoDELFIA  (Perkin Elmer, Finland) | NA |  |  | Fluorometry, AutoDELFIA  (Perkin Elmer, Finland) |  |
| Gärtner, 2002 ^25^ | 0.4 | - | 4.0 | NA | 3.5 | - | 6.6 | NA | 10.3 | - | 23.2 | NA |  |
| Hu, 2021 ^6^ | 0.27 | - | 4.2 | ICL, Cobas e601 reagent kits (Roche, Germany) | 3.1 | - | 6.9 | ICL, Cobas e601 reagent kits (Roche, Germany) | 12.0 | - | 22.0 | ICL, Cobas e601 reagent kits (Roche, Germany) |  |
| Kachouei, 2018 ^55^ | 0.3 | - | 2.0 | NA | NA |  |  | NA | NA |  |  | NA |  |
| Karanikas, 2008 ^40^ | 0.4 | - | 4.0 | ICL, Immulite 2000 (Siemens, UK) | NA |  |  | ICL, Immulite 2000 (Siemens, UK) | 10.3 |  | 24.5 | ICL, Immulite 2000 (Siemens, UK) |  |
| Karimi, 2019 ^50^ | NA |  |  | RI, details missing, (Beckman Coulter, Czech Republic) | NA |  |  | NA | NA |  |  | NA |  |
| Krysiak, 2012 ^8^ | 0.4 | - | 4.0 | CL, details missing (Roche Diagnostics, UK) | 2.4 | - | 6.0 | CL, details missing (Roche Diagnostics, UK) | 9.0 | - | 25.0 | CL, details missing (Roche Diagnostics, UK) |  |
| Kyrgios, 2019 ^10^ | 0.4 | - | 5.0 | ICL, Immulite 2000 (Siemens, UK) | NA |  |  | NA | 10.6 | - | 20.7 | ICL, Immulite 2000 (Siemens, UK) |  |
| Mahmoodianfard, 2015 ^54^ | ≤4.0 |  |  | NA | NA |  |  | RI, details missing (Beckman Coulter, Czech Republic) | NA |  |  | RI, details missing (Beckman Coulter, Czech Republic) |  |
| Mahmoudi, 2021 ^12^ | ≤4.0 |  |  | CL, Liaison (DiaSorin, Italy) | NA |  |  | NA | NA |  |  | NA |  |
| Mantovani, 2019 ^38^ | 0.35 | - | 4.94 | ICL, details missing (Rome, Italy) | NA |  |  | ICL, details missing (Abbott, Italy) | 9.0 | - | 19.1 | ICL, details missing (Abbott, Italy) |  |
| Mao, 2016 ^34^ | * |  |  | Modular Analytics E170  (Roche, Germany) | * |  |  | NA | * |  |  | Modular Analytics E170  (Roche, Germany) |  |
| Nacamulli, 2010 ^39^ | 0.28 | - | 4.2 | CL, ECLIA (Roche, Germany) | NA |  |  | NA | 12.0 | - | 22.0 | CL, ECLIA (Roche, Germany) |  |
| Negro, 2007 ^14^ | 0.27 | - | 4.0 | ICL, details missing (Roche, Switzerland) | NA |  |  | NA | 12.0 | - | 33.5 | ICL, details missing (Roche, Switzerland) |  |
| Negro, 2016 ^35^ | ≤4.0 |  |  | ICL, details missing (Roche, Switzerland) | NA |  |  | NA | NA |  |  | ICL, details missing (Roche, Switzerland) |  |
| Pilli, 2015 ^4^ | NA |  |  | ICL, Immulite 2000 (Siemens, USA) | NA |  |  | ICL, Immulite 2000 (Siemens, USA) | NA |  |  | ICL, Immulite 2000 (Siemens, USA) |  |
| Pirola, 2016 ^42^ | ≤4.0 |  |  |  | NA |  |  | NA | 10.3 | - | 24.5 |  |  |
| Preda, 2017 ^41^ | 0.4 | - | 4.0 |  | NA |  |  | NA | NA |  |  |  |  |
| Shabalina, 2019 ^47^ | 0.4 | - | 4.0 | ICL, Immulite 2000 (Siemens, USA) | NA |  |  | NA | NA |  |  | ICL, Immulite 2000 (Siemens, USA) |  |
| Sun, 2021 ^43^ | ≤4.8 |  |  | ICL, Immulite 2000 (Siemens, USA) | NA |  |  | NA | NA |  |  | ICL, Immulite 2000 (Siemens, USA) |  |
| Tian, 2020 ^36^ | 0.3 | - | 5.0 | CL, ADVIA Centaur (Siemens, USA) | 3.5 | - | 6.5 | CL, ADVIA Centaur (Siemens, USA) | 11.5 | - | 23.6 | CL, ADVIA Centaur (Siemens, USA) |  |
| Turker, 2006 ^59^ | 0.17 | - | 4.05 | RI, details missing (Immunotech, Czech Republic) | 2.5 | - | 5.8 | RI, details missing (Immunotech, Czech Republic) | 11.5 | - | 23.0 | RI, details missing (Immunotech, Czech Republic)) |  |
| Wang, 2018 ^19^ | 0.3 | - | 4.8 | ICL, details missing (Diagnostic Products Corporation, USA) | NA |  |  | NA | 10.3 | - | 24.5 | ICL, details missing (Diagnostic Products Corporation, USA) |  |
| Wu, 2018 ^53^ | NA |  |  | NA | NA |  |  | NA | NA |  |  | NA |  |
| Yu, 2017 ^57^ | 0.4 | - | 4.0 | NA | NA |  |  | NA | NA |  |  | NA |  |
| Zhang, 2020 ^37^ | NA |  |  | CP, details missing | NA |  |  | NA | NA |  |  | CP, details missing |  |
| Zhu, 2012 ^51^ | 0.3 | - | 4.8 | ICL, Immulite 2000 (Siemens, China) | NA |  |  | NA | 10.3 | - | 24.5 | ICL, Immulite 2000 (Siemens, China) |  |

CMIA = Chemiluminescent Microparticle Immunoassay; CL = Chemiluminescence; CP = Chemical photometry; EIA = enzyme immunometric assay; fT3 = Free triiodothyronine; fT4 = Free thyroxine; ICL = Immunochemiluminescence; RI = Radioimmunoassay; TGAb = Thyroglobulin antibodies; THRT = Thyroid hormone replacement therapy; TPOAb = Thyroid peroxidase antibodies; TSH = Thyroid-stimulating hormone; TTH = Total thyroid hormones; NA = Not applicable.

**Table S4.** Mean blood levels of selenium [μg/L] of intervention and control group, assay used for quantification and selenium status (sufficient or insufficient) of each included study. Severely selenium deficient = 0 to <80 μg/L(below optimal GPX acitivity^60,61^); Mildly selenium deficient = 80 to <120 μg/L(below maximal SELENP concentration); Selenium sufficient = ≥120 μg/L (selenium supplementation with serum or plasma selenium levels of below 122 µg/L associated with various health benefits with no extra risk^62^).

| **Author, Year** | **Mean Baseline Selenium [μg/L]** | **Assay** | **Selenium status** |
| --- | --- | --- | --- |
| Anastasilakis, 2012 ^33^ | Intervention: 83.3 Control: 83.2 Overall: 83.3 | ICP-MS, Agilent 7500 (Agilent, US) | Mildly deficient |
| De Farias, 2015 ^44^ | Intervention: 36.8 Control: 36.8 Overall: 36.8 | Atomic spectrometry, details missing | Severely deficient |
| Eskes, 2014 ^46^ | Intervention: 74.8 Control: 78.0 Overall: 76.4 | Reflection X-ray fluorescence analysis; details missing | Severely deficient |
| Gärtner, 2002 ^25^ | Intervention: 75.7 Control: 79.4 Overall: 77.6 | AS; details missing | Severely deficient |
| Hu, 2021 ^6^ | Intervention: 75.7 Control: 79.4 Overall: 77.6 | ICP-MS; details missing | Severely deficient |
| Kachouei, 2018 ^55^ | Intervention: 86.5 Control: 90.7 Overall: 88.6 | Details missing | Mildly deficient |
| Karanikas, 2008 ^40^ | Intervention: 75.0 Control: 76.0 Overall: 75.5 | AS, details missing | Severely deficient |
| Karimi, 2019 ^50^ | Intervention: 90.1 Control: 98.0 Overall: 93.3 | AS, AA500 (PG Instruments, UK) | Mildly deficient |
| Mahmoodianfard, 2015 ^54^ | Intervention: 73.6 Control: 81.9 Overall: 77.1 | AS, AA6701  (Shimadzu Corp, Japan) | Severely deficient (Intervention);  Mildly deficient (Control);  Severely deficient (Overall) |
| Mantovani, 2019 ^38^ | Intervention: 70.1 Control: 70.26 Overall: 70.2 | AS, 8220 Titan HG-AFS (FullTech Instruments, Italy) | Severely deficient |
| Mao, 2016 ^34^ | Intervention: 283.9 Control: 261.8 Overall: 271.8 | ICP-MS, SCIEX (Perkin-Elmer, UK) | Sufficient |
| Negro, 2007 ^14^ | Intervention: 80.9 Control: 78.2 Overall: 79.6 | AS, details missing (Australia) | Severely deficient |
| Pilli, 2015 ^4^ | Intervention: 81.6 Control: 82.1 Overall: 81.8 | AS, 8220 Titan HG-AFS (Beijing Titan Instruments Co, China) | Mildly deficient |
| Preda, 2017 ^41^ | Intervention: 257.7 Control: 236.5 Overall: 249.1 | AS, HR-CS-AAS contra 600  (Analytic Jena, Germany) | Sufficient |
| Tian, 2020 ^36^ | Intervention: 109.8 Control: 123.1 Overall: 115.6 | AS, details missing (Biosyn, Germany) | Mildly deficient (Intervention); Sufficient (Control); Mildly deficient (Overall) |
| Wang, 2018 ^19^ | Intervention: 100.8 (SH), 91.6 (OH) Control: 110.6 (SH), 95.7 (OH) Overall: 105.8 (SH), 93.0 (OH) | ICP-MS, details missing | Mildly deficient |
| Yu, 2017 ^57^ | Intervention: 28.2  Control: 27.9 Overall: 28.1 | AS, AFS-9130  (Jitian instrument Co, China) | Severely deficient |
| Zhu, 2012 ^51^ | Intervention: 89.0 (E/SH), 91.0 (OH) Control: 88.0 (E/SH), 94.0 (OH) Overall: 88.6 (SH), 92.1 (OH) | ICP-MS, details missing | Mildly deficient |

AS = Atomic spectrometry; E = Euthyroidism; ICP-MS = Inductively coupled plasma-mass spectrometry; OH = Overt hypothyroidism; Overall = Intervention and control group combined; SH = Subclinical hypothyroidism.

**Table S5.** **Subgroup and sensitivity analyses on the association of selenium supplementation with Hashimoto’s thyroiditis assessed by TSH, fT4, TPOAb, and TGAb.**

| **Study characteristics** | **N cohorts** | **N participants** | **Pooled result**  **(SMD 95% CI)** | **Heterogeneity I^2^ (p-value)** | **Moderator test**  **p-value** | |
| --- | --- | --- | --- | --- | --- | --- |
| ***TSH*** | | | | | |  |
| All participants | 26 | 2063 | -0.21 [-0.43, 0.01] | 59.3% (<0.01) |  | |
| Intervention dose |  |  |  |  | 0.80 | |
| >100 μg/d | 23 | 1534 | -0.21 [-0.45, 0.02] | 49.6% (<0.01) |  | |
| 80-100 μg/d | 7 | 609 | -0.16 [-0.48, 0.16] | 37.7% (0.16) |  | |
| Intervention duration |  |  |  |  | 0.25 | |
| 6-12 months | 19 | 1389 | -0.29 [-0.63, 0.04] | 72.6% (<0.01) |  | |
| <6 months | 20 | 1616 | **-0.18 [-0.33, -0.03]** | 0.0% (0.46) |  | |
| Thyroid status |  |  |  |  | 0.60 | |
| Overt hypothyroidism | 8 | 495 | -0.15 [-0.66, 0.37**]** | 61.0% (0.01) |  | |
| Euthyroidism or subclinical hypothyroidism | 15 | 1299 | -0.19 [-0.40, 0.02] | 28.9% (0.01) |  | |
| Mixed | 1 | 71 | 0.16 [-0.49, 0.81] | 0.0% (1.00) |  | |
| Not stated | 2 | 198 | -0.55 [-1.73, 0.62] | 86.8% (<0.01) |  | |
| Thyroid status (without thyroid hormone replacement therapy |  |  |  |  | 0.66 | |
| Overt hypothyroidism | 1 | 80 | -0.06 [-0.77, 0.65] | 0.0% (1.00) |  | |
| Euthyroidism or subclinical hypothyroidism | 6 | 789 | **-0.23 [-0.43, -0.02]** | 0.0% (0.39) |  | |
| Sex distribution |  |  |  |  | 0.68 | |
| 100% females | 12 | 909 | -0.16 [-0.36, 0.03] | 0.0% (0.05) |  | |
| 75 to <100% females | 8 | 736 | -0.19 [-0.61, 0.24] | 72.0% (<0.01) |  | |
| <70 % females | 5 | 338 | -0.15 [-1.23, 0.92] | 82.9% (<0.01) |  | |
| Not stated | 1 | 80 | -0.06 [-0.77, 0.65] | 0.0% (1.00) |  | |
| Age group |  |  |  |  | 0.08 | |
| Adults (≥18 years) | 20 | 1438 | **-0.35 [-0.59, -0.10]** | 52.3% (<0.01) |  | |
| Minors (<18 years) | 2 | 118 | 0.23 [-0.29, 0.75] | 0.0% (0.72) |  | |
| Mixed | 4 | 507 | 0.07 [-0.25, 0.39] | 21.8% (0.34) |  | |
| Thyroid hormone replacement therapy |  |  |  |  | 0.42 | |
| With | 12 | 794 | -0.25 [-0.63, 0.14] | 59.6% (<0.01) |  | |
| Mixed | 3 | 180 | 0.21 [-0.21, 0.62] | 0.0% (0.71) |  | |
| Without | 7 | 869 | **-0.21 [-0.41, -0.02]** | 0.0% (0.49) |  | |
| Not stated | 4 | 220 | -1.02 [-2.72, 0.69] | 93.1% (<0.01) |  | |
| Selenium status |  |  |  |  | 0.07 | |
| Severly deficient (<80 μg/L) | 7 | 358 | -0.14 [-0.51, 0.23] | 32.7% (0.18) |  | |
| Mildly deficient (80 to <120 μg/L) | 6 | 592 | -0.11 [-0.37, 0.14] | 0.0% (0.17) |  | |
| Sufficient (≥120 μg/L) | 1 | 100 | **-0.64 [-1.23, -0.05]** | 0.0% (1.00) |  | |
| Not stated | 12 | 1013 | -0.30 [-0.72, 0.11] | 75.5% (<0.01) |  | |
| Selenium compound |  |  |  |  | **0.03** | |
| Selenomethionine | 11 | 903 | -0.32 [-0.65, 0.01] | 59.6% (<0.01) |  | |
| Selenium yeast | 5 | 615 | -0.24 [-0.51, 0.03] | 12.3% (0.29) |  | |
| Sodium selenite | 7 | 427 | 0.22 [-0.08, 0.52] | 0.0% (0.38) |  | |
| Others or not stated | 3 | 118 | -0.83 [-1.87, 0.21] | 67.0% (0.05) |  | |
| Blinding |  |  |  |  | 0.29 | |
| Blinded | 18 | 1397 | -0.10 [-0.35, 0.16] | 54.7% (<0.01) |  | |
| Non-blinded | 4 | 277 | -0.89 [-2.56, 0.79] | 94.0% (<0.01) |  | |
| Not stated | 4 | 389 | **-0.46 [-0.76, -0.16]** | 0.0% (0.78) |  | |
| ***fT4*** | | | | | |  |
| All participants | 21 | 1664 | 0.05 [-0.15, 0.25] | 32.5% (0.08) |  | |
| Intervention dose |  |  |  |  | 0.87 | |
| >100 μg/d | 20 | 1395 | 0.07 [-0.15, 0.29] | 34.4% (0.06) |  | |
| 80-100 μg/d | 3 | 309 | 0.01 [-0.31, 0.32] | 0.0% (0.70) |  | |
| Intervention duration |  |  |  |  | 0.41 | |
| 6-12 months | 18 | 1243 | 0.08 [-0.16, 0.32] | 39.1% (0.04) |  | |
| <6 months | 18 | 1456 | -0.19 [-0.75, 0.38] | 91.0% (<0.01) |  | |
| Thyroid status |  |  |  |  | **0.03** | |
| Overt hypothyroidism | 7 | 675 | -0.22 [-0.64, 0.20] | 38.2% (0.14) |  | |
| Euthyroidism or subclinical hypothyroidism | 13 | 918 | 0.18 [-0.01, 0.37] | 0.0% (0.75) |  | |
| Mixed | 1 | 71 | -0.06 [-0.71, 0.59] | 0.0% (1.00) |  | |
| Thyroid status (without thyroid hormone replacement therapy |  |  |  |  | 0.52 | |
| Overt hypothyroidism | 1 | 80 | 0.00 [-0.71, 0.71] | 0.0% (1.00) |  | |
| Euthyroidism or subclinical hypothyroidism | 6 | 543 | 0.18 [-0.06, 0.42] | 0.0% (0.68) |  | |
| Sex distribution |  |  |  |  | 0.13 | |
| 100% females | 12 | 865 | 0.15 [-0.16, 0.46] | 49.9% (<0.01) |  | |
| 70 to <100% females | 5 | 495 | 0.06 [-0.20, 0.32] | 0.0% (0.82) |  | |
| <70 % females | 3 | 224 | -0.41 [-1.19, 0.37] | 7.6% (0.44) |  | |
| Not stated | 1 | 80 | 0.00 [-0.71, 0.71] | 0.0% (1.00) |  | |
| Age group |  |  |  |  | **<0.01** | |
| Adults (≥18 years) | 17 | 1152 | **0.19 [0.01, 0.38]** | 0.0% (0.79) |  | |
| Minors (<18 years) | 1 | 71 | -0.06 [-0.71, 0.59] | 0.0% (1.00) |  | |
| Mixed | 3 | 441 | **-0.44 [-0.80, -0.08]** | 25.3% (0.32) |  | |
| Thyroid hormone replacement therapy |  |  |  |  | 0.57 | |
| With | 10 | 842 | -0.08 [-0.43, 0.27] | 38.2% (0.09) |  | |
| Mixed | 1 | 71 | -0.06 [-0.71, 0.59] | 0.0% (1.00) |  | |
| Without | 7 | 623 | 0.16 [-0.06, 0.39] | 0.0% (0.76) |  | |
| Not stated | 3 | 128 | 0.23 [-0.63, 1.09] | 55.9% (0.10) |  | |
| Selenium status |  |  |  |  | 0.05 | |
| Severly deficient (<80 μg/L) | 6 | 315 | 0.31 [-0.00, 0.62] | 0.0% (0.93) |  | |
| Mildly deficient (80 to <120 μg/L) | 3 | 434 | **-0.44 [-0.84, -0.03]** | 26.3% (0.31) |  | |
| Not stated | 12 | 915 | 0.09 [-0.12, 0.30] | 0.0% (0.53) |  | |
| Selenium compound |  |  |  |  | 0.27 | |
| Selenomethionine | 9 | 724 | 0.16 [-0.07, 0.39] | 0.0% (0.66) |  | |
| Selenium yeast | 3 | 454 | -0.20 [-0.81, 0.41] | 72.6% (0.02) |  | |
| Sodium selenite | 6 | 408 | 0.06 [-0.27, 0.38] | 0.0% (0.51) |  | |
| Selenious acid | 3 | 118 | 0.26 [-0.43, 0.96] | 0.0% (0.40) |  | |
| Blinding |  |  |  |  | 0.63 | |
| Blinded | 15 | 1172 | 0.01 [-0.22, 0.25] | 35.1% (0.11) |  | |
| Non-blinded | 4 | 235 | 0.22 [-0.53, 0.96] | 50.3% (0.13) |  | |
| Not stated | 2 | 257 | 0.06 [-0.31, 0.43] | 0.0% (0.87) |  | |
| ***fT3*** |  |  |  |  |  | |
| All participants | 11 | 658 | 0.51 [-0.11, 1.13] | 83.5% (<0.01) |  | |
| Intervention dose |  |  |  |  | N.A. | |
| >100 μg/d | 11 | 658 | 0.51 [-0.11, 1.13] | 84.0% (<0.01) |  | |
| Intervention duration |  |  |  |  | 0.88 | |
| 6-12 months | 10 | 468 | 0.40 [-0.23, 1.03] | 78.9% (<0.01) |  | |
| <6 months | 11 | 658 | 0.46 [-0.17, 1.10] | 84.7% (<0.01) |  | |
| Thyroid status |  |  |  |  | 0.84 | |
| Overt hypothyroidism | 4 | 222 | 0.60 [-0.44, 1.64] | 76.4% (0.01) |  | |
| Euthyroidism or subclinical hypothyroidism | 7 | 436 | 0.46 [-0.37, 1.28] | 87.4% (<0.01) |  | |
| Thyroid status (without thyroid hormone replacement therapy |  |  |  |  | N.A. | |
| Euthyroidism or subclinical hypothyroidism | 3 | 239 | 1.01 [-0.60, 2.71] | 95.2% (<0.01) |  | |
| Sex distribution |  |  |  |  | 0.12 | |
| 100% females | 8 | 404 | 0.30 [-0.47, 1.08] | 84.4% (<0.01) |  | |
| 70 to <100% females | 1 | 90 | 0.39 [-0.20, 0.98] | 0.0% (1.00) |  | |
| <70 % females | 2 | 164 | **1.39 [0.22, 2.56]** | 63.1% (0.10) |  | |
| Age group |  |  |  |  | N.A. | |
| Adults (≥18 years) | 11 | 658 | 0.51 [-0.11, 1.13] | 83.5% (<0.01) |  | |
| Thyroid hormone replacement therapy |  |  |  |  | 0.62 | |
| With | 6 | 368 | 0.29 [-0.43, 1.01] | 73.6% (<0.01) |  | |
| Without | 3 | 239 | 1.01 [-0.60, 2.71] | 95.2% (<0.01) |  | |
| Not stated | 2 | 51 | 0.36 [-0.42, 1.14] | 0.0% (0.66) |  | |
| Selenium status |  |  |  |  | 0.16 | |
| Severly deficient (<80 μg/L) | 4 | 218 | 0.10 [-0.28, 0.48] | 0.0% (0.57) |  | |
| Mildly deficient (80 to <120 μg/L) | 1 | 70 | **0.88 [0.12, 1.64]** | 0.0% (1.00) |  | |
| Not stated | 6 | 370 | 0.80 [0.28, 1.89] | 88.3% (<0.01) |  | |
| Selenium compound |  |  |  |  | 0.76 | |
| Selenomethionine | 5 | 276 | 0.56 [-0.62, 1.75] | 89.4% (<0.01) |  | |
| Selenium yeast | 1 | 90 | 0.39 [-0.20, 0.98] | 0.0% (1.00) |  | |
| Sodium selenite | 3 | 234 | 0.90 [-0.24, 2.03] | 81.5% (<0.01) |  | |
| Selenious acid | 2 | 58 | -0.23 [-0.98, 0.52] | 0.0% (0.62) |  | |
| Blinding |  |  |  |  | 0.62 | |
| Blinded | 7 | 423 | 0.39 [-0.48, 1.25] | 88.0% (<0.01) |  | |
| Non-blinded | 4 | 235 | 0.72 [-0.06, 1.49] | 59.0% (0.08) |  | |
| ***TPOAb*** |  |  |  |  |  | |
| All participants | 29 | 2358 | **-0.96 [-1.36, -0.56]** | 89.6% (<0.01) |  | |
| Intervention dose |  |  |  |  | 0.28 | |
| >100 μg/d | 25 | 1828 | **-1.02 [-1.54, -0.50]** | 92.2% (<0.01) |  | |
| 80-100 μg/d | 6 | 568 | -0.52 [-1.10, 0.07] | 80.2% (<0.01) |  | |
| Intervention duration |  |  |  |  | 0.28 | |
| 6-12 months | 24 | 1657 | **-0.73 [-1.23, -0.23]** | 90.6% (<0.01) |  | |
| <6 months | 24 | 1970 | **-0.59 [-0.98, -0.20]** | 87.6% (<0.01) |  | |
| Thyroid status |  |  |  |  | **0.03** | |
| Overt hypothyroidism | 6 | 436 | **-1.39 [-2.39, -0.38]** | 90.5% (<0.01) |  | |
| Euthyroidism or subclinical hypothyroidism | 19 | 1591 | **-0.57 [-0.90, -0.24]** | 77.9% (<0.01) |  | |
| Mixed | 2 | 131 | **-1.50 [-2.03, -0.97]** | 4.1% (0.31) |  | |
| Not stated | 2 | 198 | -3.02 [-8.74, 2.60] | 98.9% (<0.01) |  | |
| Sex distribution |  |  |  |  | 0.38 | |
| 100% females | 15 | 1189 | **-0.60 [-1.00, -0.20]** | 79.5% (<0.01) |  | |
| 70 to <100% females | 9 | 795 | **-1.38 [-2.54, -0.23]** | 96.2% (<0.01) |  | |
| <70 % females | 5 | 372 | **-1.28 [-2.53, -0.04]** | 92.3% (<0.01) |  | |
| Age group |  |  |  |  | 0.30 | |
| Adults (≥18 years) | 20 | 1510 | **-1.21 [-1.83, -0.58]** | 93.0% (<0.01) |  | |
| Minors (<18 years) | 2 | 118 | -0.70 [-1.87, 0.46] | 78.8% (0.03) |  | |
| Mixed | 7 | 728 | -0.33 [-0.71, 0.06] | 64.8% (0.02) |  | |
| Thyroid hormone replacement therapy |  |  |  |  | 0.27 | |
| With | 12 | 918 | **-1.51 [-2.44, -0.57]** | 94.5% (<0.01) |  | |
| Mixed | 3 | 180 | -0.63 [-1.33, 0.08] | 63.8% (0.06) |  | |
| Without | 7 | 865 | **-0.74 [-1.47, -0.00\|** | 91.6% (<0.01) |  | |
| Not stated | 7 | 393 | -0.38 [-0.85, 0.09] | 59.2% (0.04) |  | |
| Selenium status |  |  |  |  | 0.67 | |
| Severly deficient (<80 μg/L) | 5 | 300 | -0.44 [-0.93, 0.06] | 54.8% (0.07) |  | |
| Mildly deficient (80 to <120 μg/L) | 8 | 726 | -0.68 [-1.47, 0.13] | 91.3% (<0.01) |  | |
| Sufficient (≥120 μg/L) | 1 | 100 | -0.19 [-0.74, 0.36] | 0.0% (1.00) |  | |
| Not stated | 15 | 1230 | **-1.36 [-2.10, -0.62]** | 93.6% (<0.01) |  | |
| Selenium compound |  |  |  |  | 0.39 | |
| Selenomethionine | 13 | 1027 | **-1.24 [-2.07, -0.40]** | 94.3% (<0.01) |  | |
| Selenium yeast | 7 | 749 | -0.38 [-0.78, 0.03] | 66.1% (0.01) |  | |
| Sodium selenite | 9 | 580 | **-1.01 [-1.81, -0.22]** | 89.8% (<0.01) |  | |
| Blinding |  |  |  |  | 0.78 | |
| Blinded | 19 | 1556 | **-1.09 [-1.75, -0.44]** | 94.1% (<0.01) |  | |
| Non-blinded | 6 | 411 | **-0.73 [-1.46, -0.01]** | 80.2% (<0.01) |  | |
| Not stated | 4 | 389 | -0.75 [-1.54, 0.03] | 83.5% (<0.01) |  | |
| ***TGAb*** |  |  |  |  |  | |
| All participants | 17 | 1283 | -0.27 [-0.59, 0.06] | 74.0% (<0.01) |  | |
| Intervention dose |  |  |  |  | 0.61 | |
| >100 μg/d | 15 | 1046 | -0.30 [-0.68, 0.09] | 77.7% (<0.01) |  | |
| 80-100 μg/d | 4 | 277 | -0.13 [-0.47, 0.22] | 0.0% (0.87) |  | |
| Intervention duration |  |  |  |  | 0.67 | |
| 6-12 months | 12 | 871 | -0.25 [-0.55, 0.04] | 56.3% (<0.01) |  | |
| <6 months | 12 | 896 | -0.19 [-0.58, 0.19] | 73.7% (<0.01) |  | |
| Thyroid status |  |  |  |  | **0.02** | |
| Overt hypothyroidism | 4 | 360 | -0.73 [-1.49, 0.04] | 77.9% (<0.01) |  | |
| Euthyroidism or subclinical hypothyroidism | 10 | 674 | -0.06 [-0.40, 0.29] | 59.1% (<0.01) |  | |
| Mixed | 1 | 71 | **-1.61 [-2.32, -0.90]** | 0.0% (1.00) |  | |
| Not stated | 2 | 198 | 0.23 [-0.18, 0.64] | 0.0% (0.42) |  | |
| Sex distribution |  |  |  |  | **0.04** | |
| 100% females | 5 | 367 | -0.01 [-0.69, 0.70] | 81.6% (<0.01) |  | |
| 70 to <100% females | 7 | 544 | -0.24 [-0.70, 0.23] | 71.6% (<0.01) |  | |
| <70 % females | 5 | 372 | -0.60 [-1.26, 0.06] | 73.1% (<0.01) |  | |
| Age group |  |  |  |  | 0.52 | |
| Adults (≥18 years) | 12 | 934 | -0.23 [-0.65, 0.18] | 77.5% (<0.01) |  | |
| Minors (<18 years) | 2 | 118 | -0.78 [-2.44, 0.88] | 89.1% (<0.01) |  | |
| Mixed | 3 | 231 | -0.07 [-0.44, 0.30] | 0.0% (0.86) |  | |
| Thyroid hormone replacement therapy |  |  |  |  | 0.83 | |
| With | 9 | 771 | -0.15 [-0.68, 0.38] | 83.2% (<0.01) |  | |
| Mixed | 3 | 180 | -0.56 [-1.61, 0.49] | 83.3% (<0.01) |  | |
| Without | 3 | 223 | **-0.45 [-0.88, -0.01]** | 24.0% (0.27) |  | |
| Not stated | 2 | 109 | -0.16 [-0.70, 0.38] | 0.0% (0.74) |  | |
| Selenium status |  |  |  |  | 0.64 | |
| Severly deficient (<80 μg/L) | 3 | 203 | 0.32 [-0.66, 1.31] | 82.7% (<0.01) |  | |
| Mildly deficient (80 to <120 μg/L) | 4 | 228 | -0.42 [-1.15, 0.31] | 70.0% (0.02) |  | |
| Not stated | 10 | 852 | -0.38 [-0.77, 0.01] | 72.9% (<0.01) |  | |
| Selenium compound |  |  |  |  | 0.97 | |
| Selenomethionine | 8 | 608 | -0.30 [-0.74, 0.14] | 71.5%(<0.01) |  | |
| Selenium yeast | 3 | 251 | -0.21 [-0.57, 0.15] | 0.0% (0.91) |  | |
| Sodium selenite | 6 | 424 | -0.27 [-1.10, 0.56] | 86.8% (<0.01) |  | |
| Blinding |  |  |  |  | 0.81 | |
| Blinded | 11 | 826 | -0.26 [-0.74, 0.22] | 82.2% (<0.01) |  | |
| Non-blinded | 4 | 360 | -0.41 [-0.94, 0.12] | 60.9% (0.07) |  | |
| Not stated | 2 | 97 | 0.01 [-0.54, 0.57] | 0.0% (0.93) |  | |

Intervention dose and Intervention duration

**Figure S1.** **Effect of selenium supplementation on thyroid-stimulating hormones [TSH, mIU/L] in Hashimoto thyroiditis with and without thyroid hormone replacement therapy (n = 2063).** Black rectangles represent SMD for each study; the size of the rectangle is proportional to the weight of the study for the pooled effect. Horizontal lines indicate 95% CI. The black diamond summarises the pooled SMD data. Control = Control group receiving placebo or nothing; SMD = Standardized mean difference; (1)/(2)/(3) indicate cohort 1, 2, and 3 of study.

**Figure S2.** **Effect of selenium supplementation on thyroid-stimulating hormones [TSH, mIU/L], stratified by thyroid status at study start, in patients with Hashimoto thyroiditis with thyroid hormone replacement therapy (n = 794).** Black rectangles represent SMD for each study; the size of the rectangle is proportional to the weight of the study for the pooled effect. Horizontal lines indicate 95% CI. The black diamond summarises the pooled SMD data. Control = Control group receiving placebo or nothing; SMD = Standardized mean difference; (1)/(2) indicate cohort 1 and 2 of study.

**Figure S3. Effect of selenium supplementation on free thyroxine [fT4, pmol/L] in Hashimoto thyroiditis with and without thyroid hormone replacement therapy (n = 1664).** Black rectangles represent SMD for each study; the size of the rectangle is proportional to the weight of the study for the pooled effect. Horizontal lines indicate 95% CI. The black diamond summarises the pooled SMD data. Control = Control group receiving placebo or nothing; SMD = Standardized mean difference; (1)/(2) indicate cohort 1 and 2 of study.

**Figure S4. Effect of selenium supplementation on free thyroxine [fT4, pmol/L], stratified by thyroid status, in Hashimoto thyroiditis without thyroid hormone replacement therapy (n = 623).** Black rectangles represent SMD for each study; the size of the rectangle is proportional to the weight of the study for the pooled effect. Horizontal lines indicate 95% CI. The black diamond summarises the pooled SMD data. Control = Control group receiving placebo or nothing; SMD = Standardized mean difference.

**Figure S5. Effect of selenium supplementation on free thyroxine [fT4, pmol/L], stratified by thyroid status, in Hashimoto thyroiditis with thyroid hormone replacement therapy (n = 842).** Black rectangles represent SMD for each study; the size of the rectangle is proportional to the weight of the study for the pooled effect. Horizontal lines indicate 95% CI. The black diamond summarises the pooled SMD data. Control = Control group receiving placebo or nothing; SMD = Standardized mean difference; (1)/(2) indicate cohort 1 and 2 of study.

**Figure S6. Effect of selenium supplementation on free triiodothyronine [fT3, pmol/L] in patients with Hashimoto thyroiditis with and without thyroid hormone replacement therapy (n = 658).** Black rectangles represent SMD for each study; the size of the rectangle is proportional to the weight of the study for the pooled effect. Horizontal lines indicate 95% CI. The black diamond summarises the pooled SMD data. Control = Control group receiving placebo or nothing; SMD = Standardized mean difference; (1)/(2) indicate cohort 1 and 2 of study.


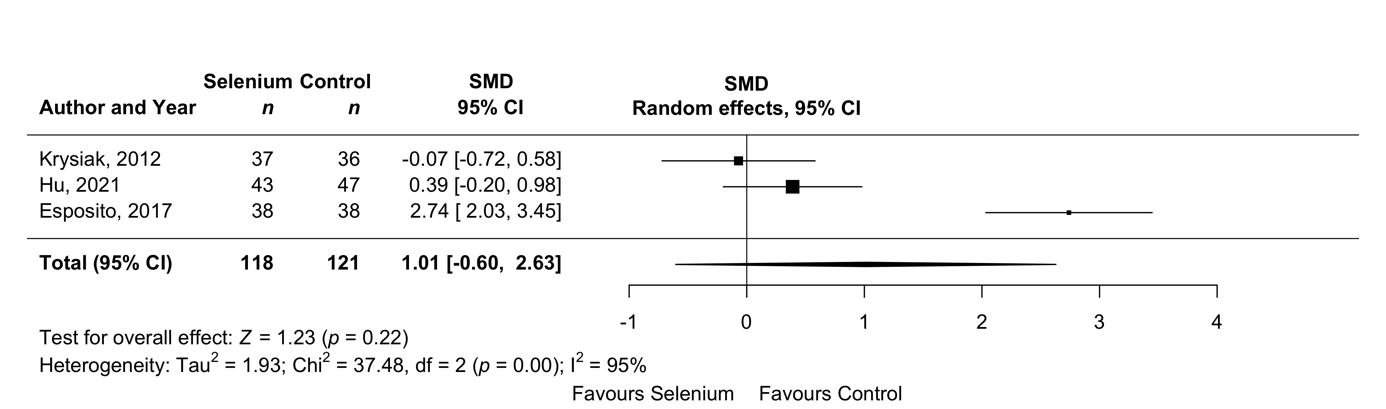


**Figure S7.** **Effect of selenium supplementation on free triiodothyronine [fT3, pmol/L] in patients (all euthyroid) with Hashimoto thyroiditis without thyroid hormone replacement therapy (n = 239).** Black rectangles represent SMD for each study; the size of the rectangle is proportional to the weight of the study for the pooled effect. Horizontal lines indicate 95% CI. The black diamond summarises the pooled SMD data. Control = Control group receiving placebo or nothing; SMD = Standardized mean difference; (1)/(2) indicate cohort 1 and 2 of study.

**Figure S8. Effect of selenium supplementation on free triiodothyronine [fT3, pmol/L], stratified by thyroid status at study start, in patients with Hashimoto thyroiditis with thyroid hormone replacement therapy (n = 368).** Black rectangles represent SMD for each study; the size of the rectangle is proportional to the weight of the study for the pooled effect. Horizontal lines indicate 95% CI. The black diamond summarises the pooled SMD data. Control = Control group receiving placebo or nothing; SMD = Standardized mean difference; (1)/(2) indicate cohort 1 and 2 of study.


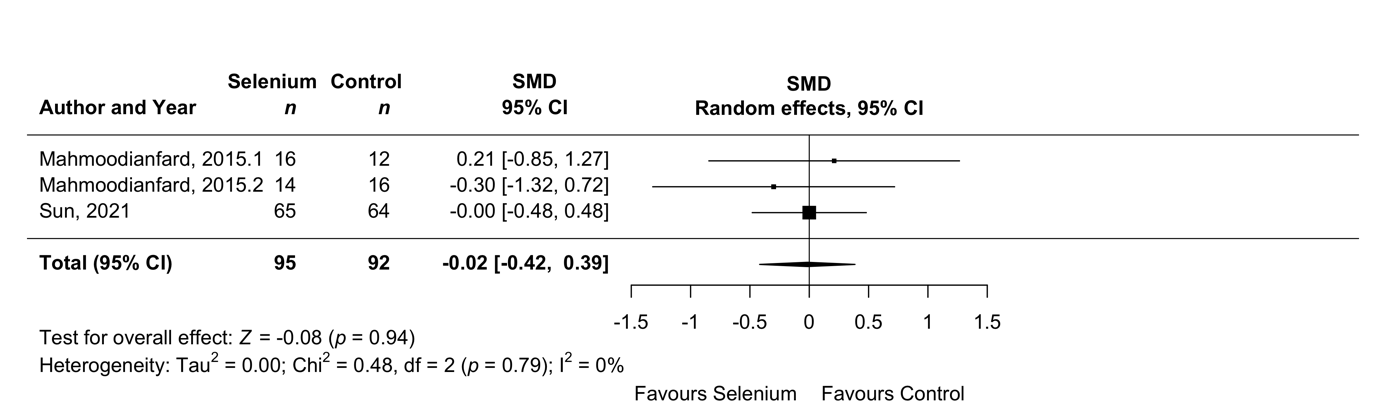


**Figure S9. Effect of selenium supplementation on total thyroxine [T4, pg/mL] in Hashimoto thyroiditis (n = 187).** Black rectangles represent SMD for each study; the size of the rectangle is proportional to the weight of the study for the pooled effect. Horizontal lines indicate 95% CI. The black diamond summarises the pooled SMD data. Control = Control group receiving placebo or nothing; SMD = Standardized mean difference; (1)/(2) indicate cohort 1 and 2 of study.


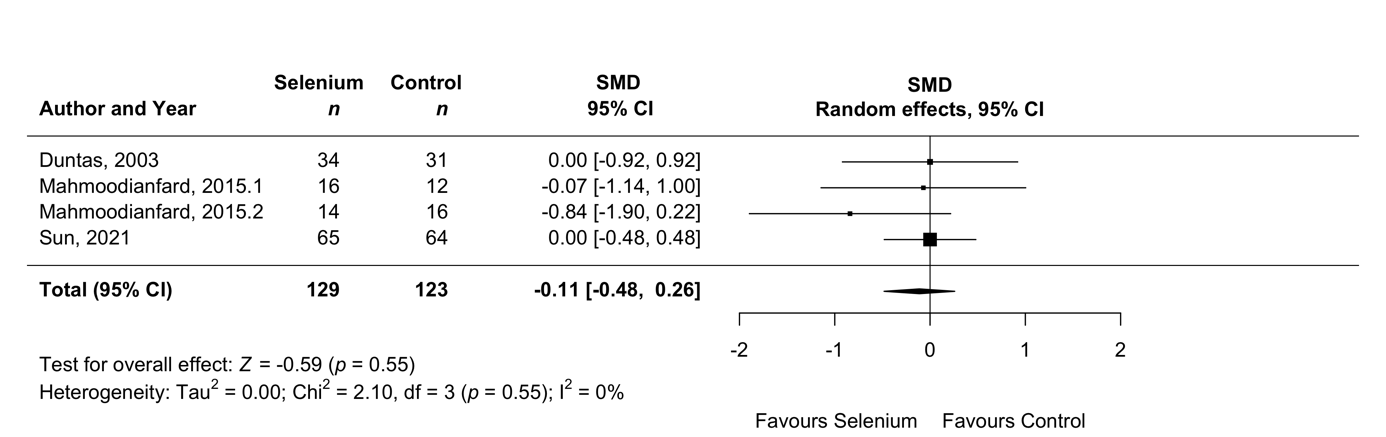


**Figure S10. Effect of selenium supplementation on total triiodothyronine [T3, nmol/L] in Hashimoto thyroiditis (n = 252).** Black rectangles represent SMD for each study; the size of the rectangle is proportional to the weight of the study for the pooled effect. Horizontal lines indicate 95% CI. The black diamond summarises the pooled SMD data. Control = Control group receiving placebo or nothing; SMD = Standardized mean difference; (1)/(2) indicate cohort 1 and 2 of study.


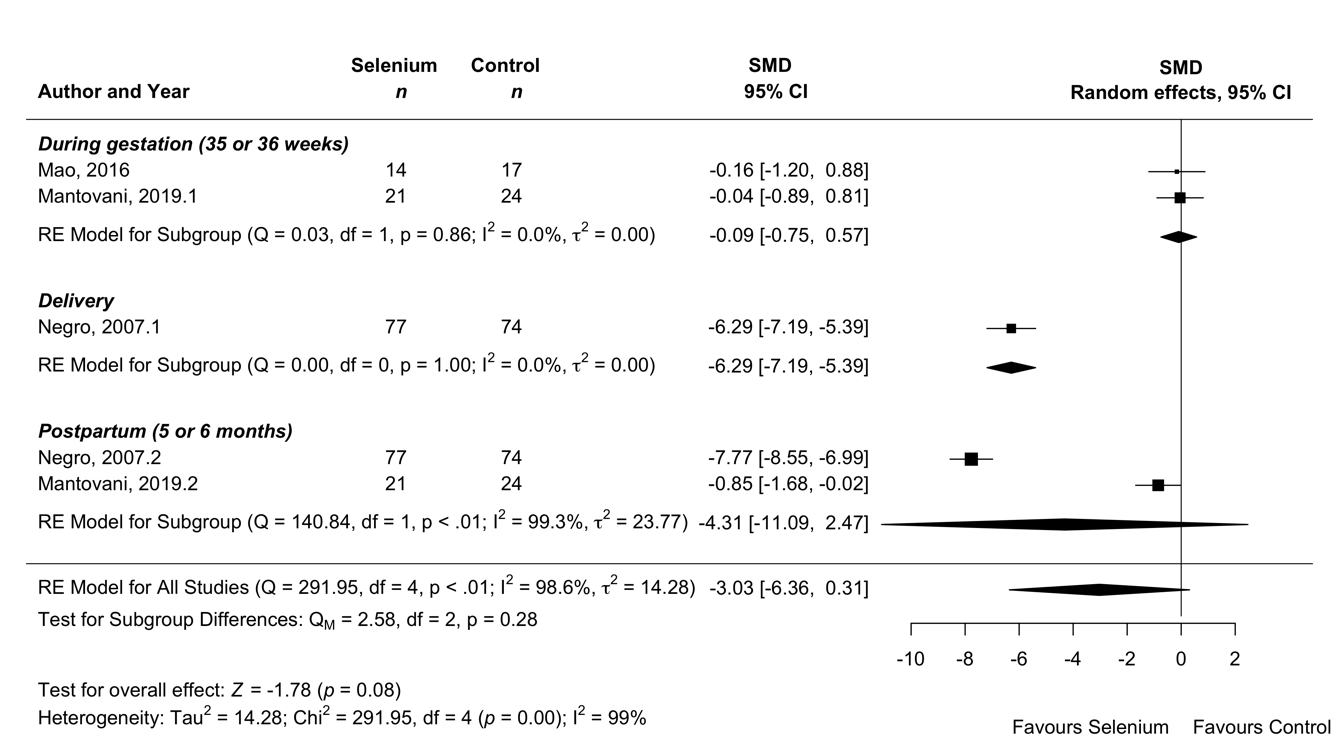


**Figure S11. Effect of selenium supplementation started during pregnancy on thyroid peroxidase antibodies [TPOAb, IU/mL] in pregnant TPOAb-positive women at different time points.** Black rectangles represent MD for each study; the size of the rectangle is proportional to the weight of the study for the pooled effect. Horizontal lines indicate 95% CI. The black diamond summarises the pooled SMD data. Control = Control group receiving placebo or nothing; SMD = Standardized mean difference; (1)/(2) indicate same cohort at different time points.


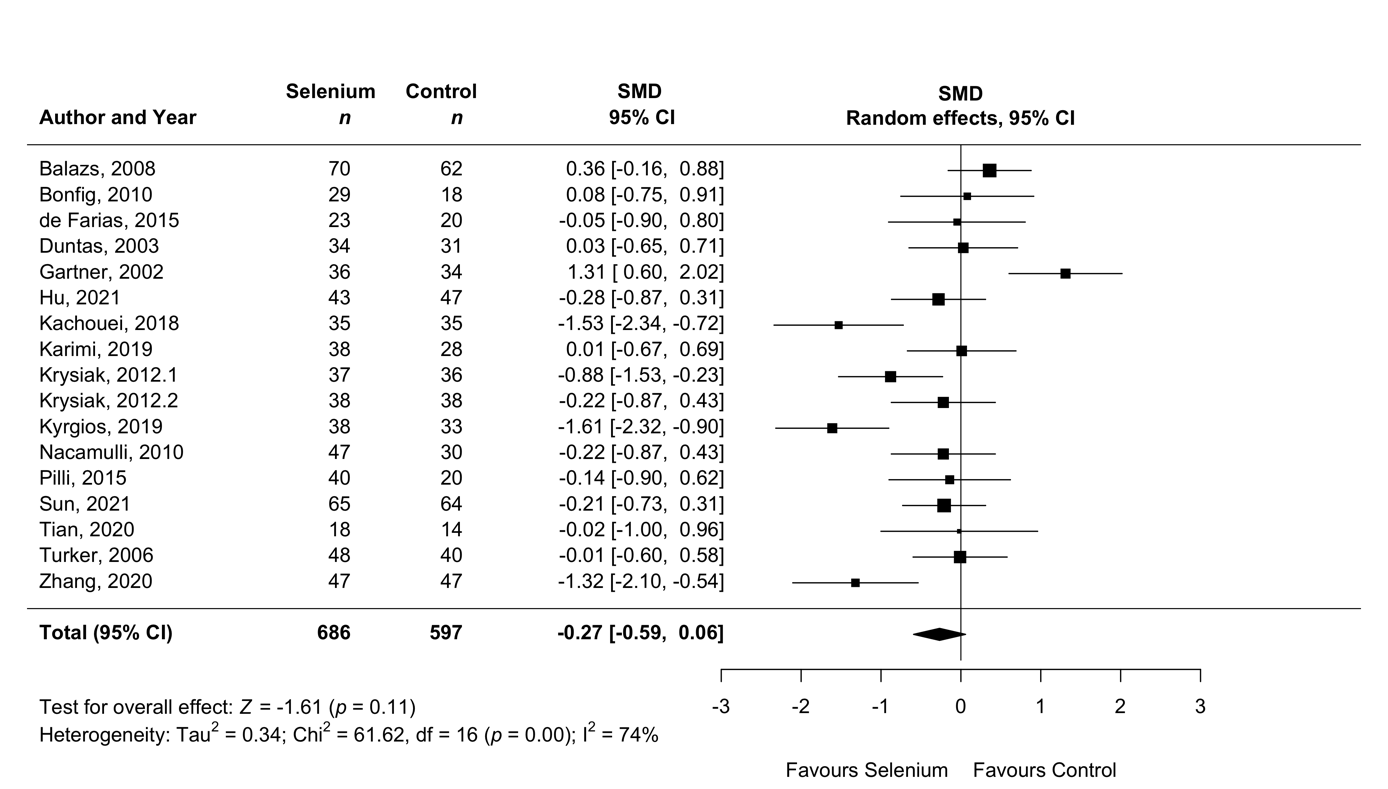


**Figure S12. Effect of selenium supplementation on thyroglobulin antibodies [TGAb, IU/mL] in Hashimoto thyroiditis (n = 1283).** Black rectangles represent SMD for each study; the size of the rectangle is proportional to the weight of the study for the pooled effect. Horizontal lines indicate 95% CI. The black diamond summarises the pooled SMD data. Control = Control group receiving placebo or nothing; SMD = Standardized mean difference; (1)/(2) indicate cohort 1 and 2 of study.

**
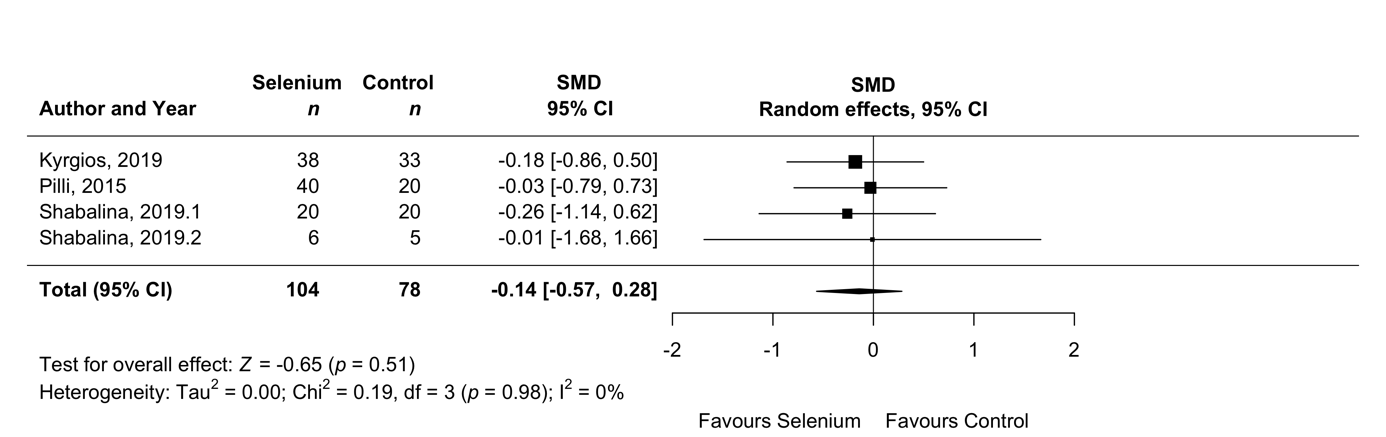
**

**Figure S13.** **Effect of selenium supplementation on thyroid volume [mL] in Hashimoto thyroiditis (n = 182).** Black rectangles represent SMD for each study; the size of the rectangle is proportional to the weight of the study for the pooled effect. Horizontal lines indicate 95% CI. The black diamond summarises the pooled SMD data of the 5 studies with adverse events. Only studies with adverse events could be pooled. Control = Control group receiving placebo or nothing; SMD = standardized mean difference; (1)/(2) indicate cohort 1 and 2 of study.

**
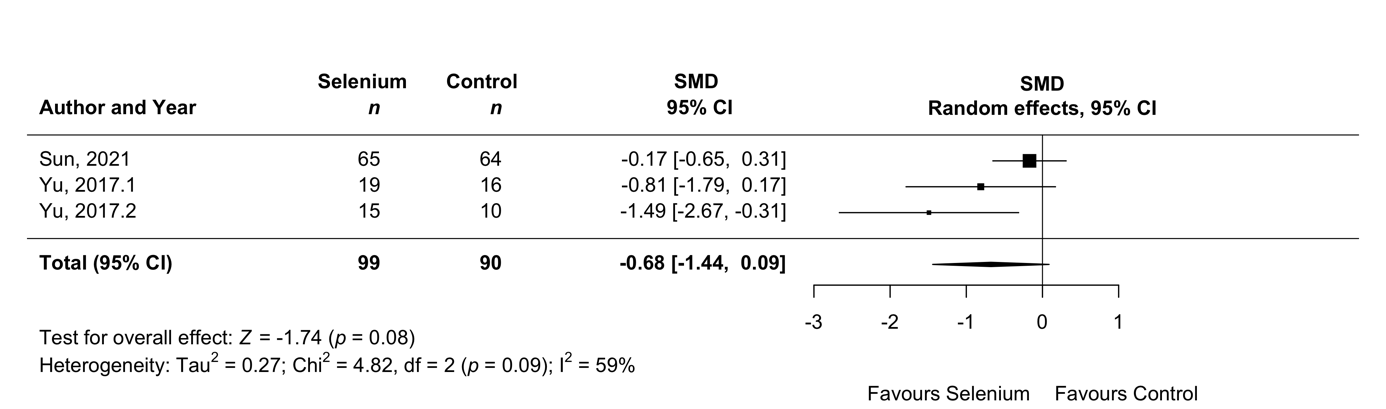
**

**Figure S14.** **Effect of selenium supplementation on interleukin-2 [IL-2; pg/mL] in Hashimoto thyroiditis (n = 189).** Black rectangles represent SMD for each study; the size of the rectangle is proportional to the weight of the study for the pooled effect. Horizontal lines indicate 95% CI. The black diamond summarises the pooled SMD data of the 5 studies with adverse events. Only studies with adverse events could be pooled. Control = Control group receiving placebo or nothing; SMD = standardized mean difference; (1)/(2) indicate cohort 1 and 2 of study.


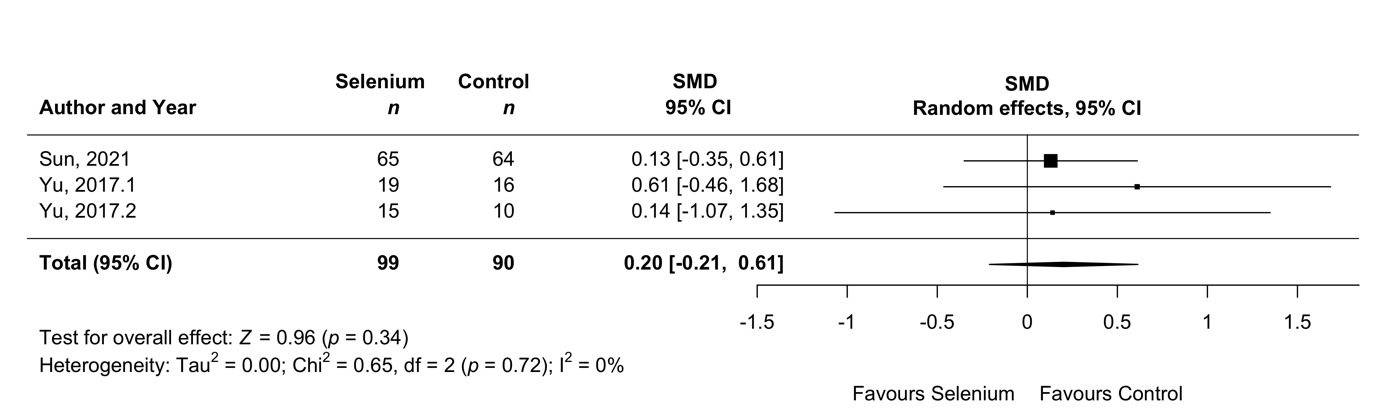


**Figure S15.** **Effect of selenium supplementation on interleukin-10 [IL-10; pg/mL] in Hashimoto thyroiditis (n = 189).** Black rectangles represent SMD for each study; the size of the rectangle is proportional to the weight of the study for the pooled effect. Horizontal lines indicate 95% CI. The black diamond summarises the pooled SMD data of the 5 studies with adverse events. Only studies with adverse events could be pooled. Control = Control group receiving placebo or nothing; SMD = standardized mean difference; (1)/(2) indicate cohort 1 and 2 of study.

**
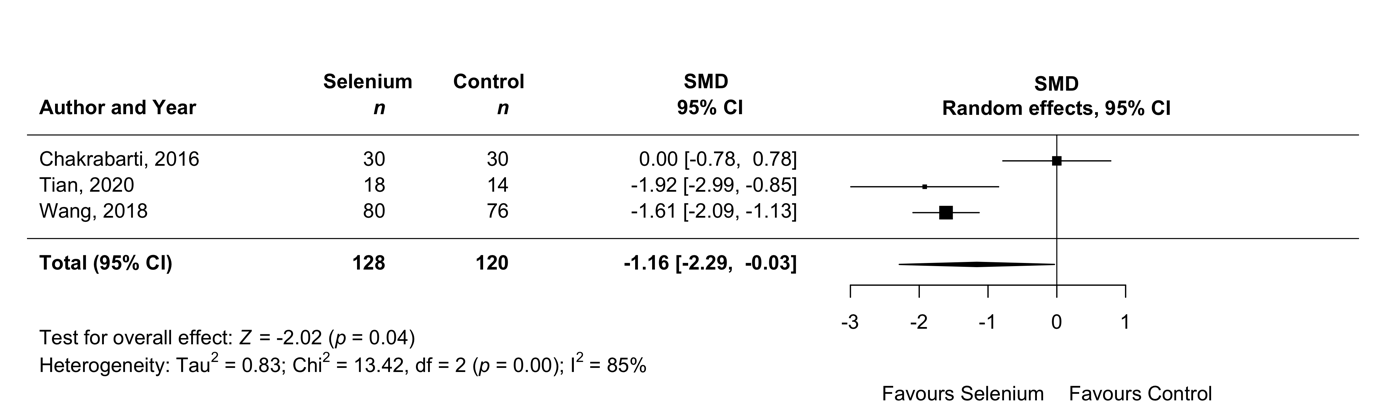
**

**Figure S16. Effect of selenium supplementation on malondialdehyde [MDA; nmol/mL] in Hashimoto thyroiditis (n = 248).** Black rectangles represent SMD for each study; the size of the rectangle is proportional to the weight of the study for the pooled effect. Horizontal lines indicate 95% CI. The black diamond summarises the pooled SMD data of the 5 studies with adverse events. Only studies with adverse events could be pooled. Control = Control group receiving placebo or nothing; SMD = standardized mean difference.


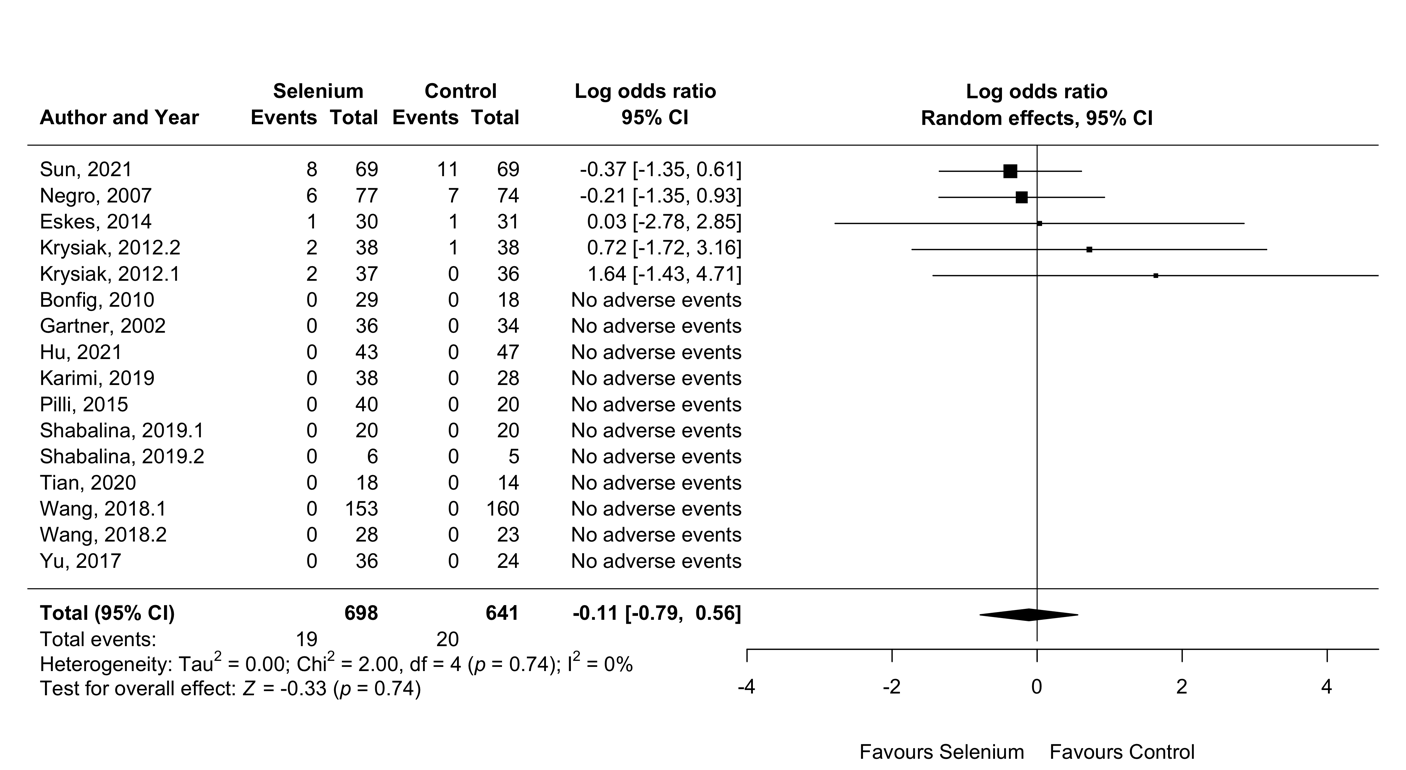


**Figure S17. Effect of selenium supplementation on adverse events (log odds ratio) in Hashimoto thyroiditis (n = 1339).** Black rectangles represent SMD for each study; the size of the rectangle is proportional to the weight of the study for the pooled effect. Horizontal lines indicate 95% CI. The black diamond summarises the pooled SMD data of the 5 studies with adverse events. Only studies with adverse events could be pooled. Control = Control group receiving placebo or nothing; SMD = standardized mean difference; (1)/(2) indicate cohort 1 and 2 of study.

| **TSH levels** |  |
| --- | --- |
| **A.1 Dose – Stratified analysis**  **** | **B.1 Duration – Stratified analysis ** |
| **A.2 Dose** **– Meta-regression**   | **B.2 Duration – Meta-regression**  |
| **C.1 Selenium status – Stratified analysis ** | **D.1 Sex distribution – Stratified analysis** **** |
| **C.2 Selenium status – Meta-regression**  | **D.2 Sex distribution – Meta-regression**   |
| **E.1 Thyroid status – Stratified analysis** **** | **F.1 Age – Stratified analysis**  |
| **G.1 Thyroid hormone replacement therapy – Stratified analysis** **** | **H.1 Selenium compound – Stratified analysis** **** |
| **I.1 Blinding – Stratified analysis ** |  |

**Figure S18. (1) Stratified forest plots and (2) scatterplots of the meta-regression analysis of SMD of thyroid-stimulating hormone (TSH) levels [mIU/L] of the moderators (A) dose, (B) duration, (C) selenium status, (D) sex distribution, (E) thyroid status, (F) age, (G) thyroid hormone replacement therapy, (H) selenium compound, and (I) blinding of the study.** In the forest plots, black rectangles represent SMD for each study; the size of the rectangle is proportional to the weight of the study for the pooled effect. Horizontal lines indicate 95% CI. The black diamond summarises the pooled SMD data. In the scatterplots, the circle represent individual studies and size of the circle is proportional to the weight of the study. The black line is the regression line and the grey area the confidence interval bounds. The *p*-value indicate result of moderator test. Control = Control group receiving placebo or nothing; TSH = thyroid-stimulating hormone.

| **fT4 levels** |  |
| --- | --- |
| **A.1 Dose – Stratified analysis ** | **B.1 Duration – Stratified analysis ** |
| **A.2 Dose – Meta-regression**  | **B.2 Duration – Meta-regression**  |
| **C.1 Selenium status – Stratified analysis ** | **D.1 Sex distribution – Stratified analysis** **** |
| **C.2 Selenium status – Meta-regression**  | **D.2 Sex distribution – Meta-regression**  |
| **E.1 Thyroid status – Stratified analysis** **** | **F.1 Age – Stratified analysis**  |
| **G.1 Thyroid hormone replacement therapy – Stratified analysis** **** | **H.1 Selenium compound – Stratified analysis** **** |
| **I.1 Blinding – Stratified analysis** **** |  |

**Figure S19. (1) Stratified forest plots and (2) scatterplots of the meta-regression analysis of SMD of free thyroxine (fT4) levels [pmol/L] of the moderators (A) dose, (B) duration, (C) selenium status, (D) sex distribution, (E) thyroid status, (F) age, (G) thyroid hormone replacement therapy, (H) selenium compound, and (I) blinding of the study.** In the forest plots, black rectangles represent SMD for each study; the size of the rectangle is proportional to the weight of the study for the pooled effect. Horizontal lines indicate 95% CI. The black diamond summarises the pooled SMD data. In the scatterplots, the circle represent individual studies and size of the circle is proportional to the weight of the study. The black line is the regression line and the grey area the confidence interval bounds. The *p*-value indicate result of moderator test. Control = Control group receiving placebo or nothing; FT4 = Free thyroxine.

| **fT3 levels** |  |
| --- | --- |
| **A.1 Dose – Stratified analysis**  **** | **B.1 Duration – Stratified analysis ** |
| **A.2 Dose – Meta-regression**  | **B.2 Duration – Meta-regression**  |
| **C.1 Selenium status – Stratified analysis ** | **D.1 Sex distribution – Stratified analysis** **** |
| **C.2 Selenium status – Meta-regression**  | **D.2 Sex distribution – Meta-regression**  |
| **E.1 Thyroid status – Stratified analysis** **** | **F.1 Age – Stratified analysis** **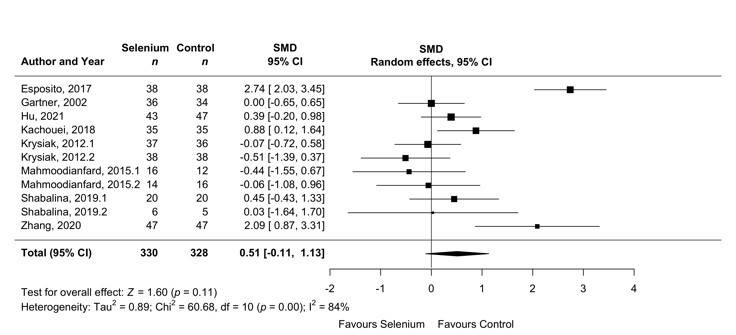**  All adults (≥18 years) |
| **G.1 Thyroid hormone replacement therapy – Stratified analysis** **** | **H.1 Selenium compound – Stratified analysis** **** |
| **I.1 Blinding – Stratified analysis** **** |  |

**Figure S20. (1) Stratified forest plots and (2) scatterplots of the meta-regression analysis of SMD of free triiodothyronine (fT3) levels [pmol/L] of the moderators (A) dose, (B) duration, (C) selenium status, (D) sex distribution, (E) thyroid status, (F) age, (G) thyroid hormone replacement therapy, (H) selenium compound, and (I) blinding of the study.** In the forest plots, black rectangles represent SMD for each study; the size of the rectangle is proportional to the weight of the study for the pooled effect. Horizontal lines indicate 95% CI. The black diamond summarises the pooled SMD data. In the scatterplots, the circle represent individual studies and size of the circle is proportional to the weight of the study. The black line is the regression line and the grey area the confidence interval bounds. The *p*-value indicate result of moderator test. Control = Control group receiving placebo or nothing; FT3 = Free triiodothyronine.

| **TPOAb levels** |  |
| --- | --- |
| **A.1 Dose – Stratified analysis ** | **B.1 Duration – Stratified analysis ** |
| **A.2 Dose – Meta-regression** | **B.2 Duration – Meta-regression**   |
| **C.1 Selenium status – Stratified analysis ** | **D.1 Sex distribution – Stratified analysis** **** |
| **C.2 Selenium status – Meta-regression**  | **D.2 Sex distribution – Meta-regression**  |
| **E.1 Thyroid status – Stratified analysis** **** | **F.1 Age – Stratified analysis**  |
| **G.1 Thyroid hormone replacement therapy – Stratified analysis** **** | **H.1 Selenium compound – Stratified analysis** **** |
| **I.1 Blinding – Stratified analysis ** |  |

**Figure S21. (1) Stratified forest plots and (2) scatterplots of the meta-regression analysis of SMD of thyroid peroxidase antibodies (TPOAb) levels [IU/mL] of the moderators (A) dose, (B) duration, (C) selenium status, (D) sex distribution, (E) thyroid status, (F) age, (G) thyroid hormone replacement therapy, (H) selenium compound, and (I) blinding of the study.** In the forest plots, black rectangles represent SMD for each study; the size of the rectangle is proportional to the weight of the study for the pooled effect. Horizontal lines indicate 95% CI. The black diamond summarises the pooled SMD data. In the scatterplots, the circle represent individual studies and size of the circle is proportional to the weight of the study. The black line is the regression line and the grey area the confidence interval bounds. The *p*-value indicate result of moderator test. Control = Control group receiving placebo or nothing; TPOAb = Thyroid peroxidase antibodies.

| **TGAb levels** |  |
| --- | --- |
| **A.1 Dose – Stratified analysis ** | **B.1 Duration – Stratified analysis**  **** |
| **A.2 Dose – Meta-regression**  | **B.2 Duration – Meta-regression**  |
| **C.1 Selenium status – Stratified analysis ** | **D.1 Sex distribution – Stratified analysis** **** |
| **C.2 Selenium status – Meta-regression**  | **D.2 Sex distribution – Meta-regression**  |
| **E.1 Thyroid status – Stratified analysis** **** | **F.1 Age – Stratified analysis**   |
| **G.1 Thyroid hormone replacement therapy – Stratified analysis** **** | **H.1 Selenium compound – Stratified analysis**  **** |
| **I.1 Blinding – Stratified analysis** **** |  |

**Figure S22. (1) Stratified forest plots and (2) scatterplots of the meta-regression analysis of SMD of thyroglobulin antibodies (TGAb) levels [IU/mL] of the moderators (A) dose, (B) duration, (C) selenium status, (D) sex distribution, (E) thyroid status, (F) age, (G) thyroid hormone replacement therapy, (H) selenium compound, and (I) blinding of the study.** In the forest plots, black rectangles represent SMD for each study; the size of the rectangle is proportional to the weight of the study for the pooled effect. Horizontal lines indicate 95% CI. The black diamond summarises the pooled SMD data. In the scatterplots, the circle represent individual studies and size of the circle is proportional to the weight of the study. The black line is the regression line and the grey area the confidence interval bounds. The *p*-value indicate result of moderator test. Control = Control group receiving placebo or nothing; TGAb = Thyroglobulin antibodies.

| **A.1 – TSH**  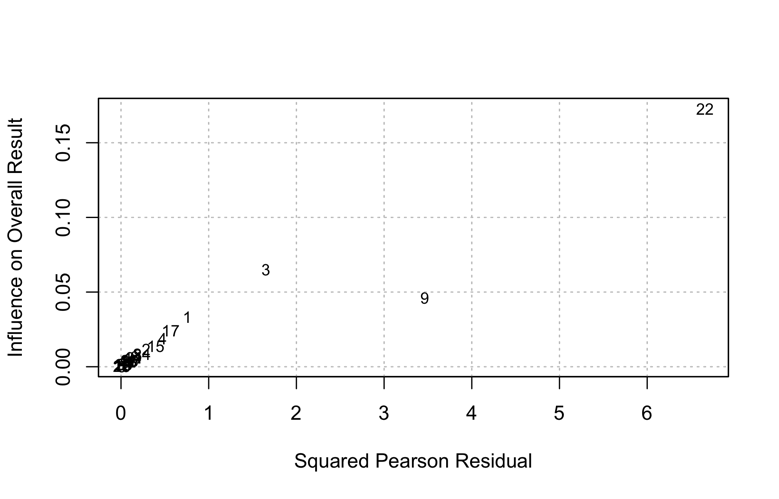 | | **A.2 – TSH**  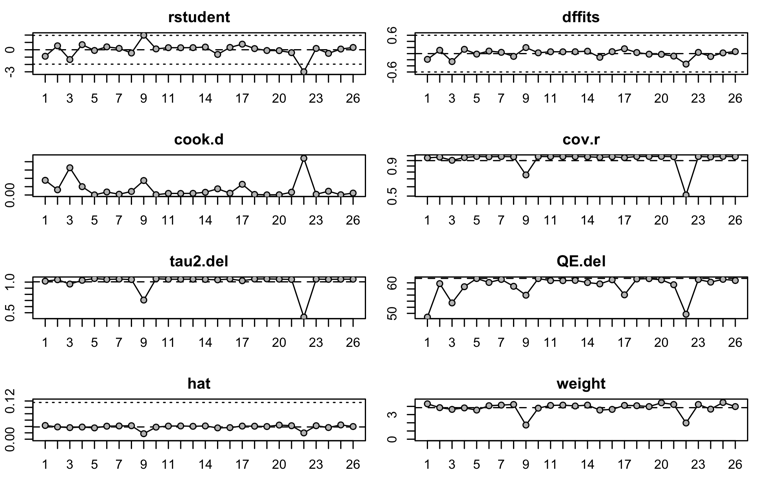 |
| --- | --- | --- |
| 1. Balazs, 2009^56^ 2. Bonfig, 2010^2^ 3. Chakrabarti, 2016^58^ 4. De Farias, 2015^44^ 5. Duntas, 2003^45^ 6. Eskes, 2014^46^ 7. Gärtner, 2002^25^ 8. Hu, 2021^6^ 9. Kachouei, 2018^55^ 10. Karanikas, 2008^40^ 11. Karimi, 2019^50^ 12. Krysiak, 2012.1^8^ 13. Krysiak, 2012.2^8^ 14. Kyrgios, 2019^10^ | 1. Mahmoodianfard, 2015.1^54^ 2. Mahmoodianfard, 2015.2^54^ 3. Nacamulli, 2010^39^ 4. Negro, 2016^35^ 5. Pilli, 2015^4^ 6. Pirola, 2016^42^ 7. Preda, 2017^41^ 8. Shabalina, 2019.2^47^ 9. Sun, 2021^43^ 10. Tian, 2020^36^ 11. Wang, 2018.1^19^ 12. Wang, 2018.2^19^ | No outlier cohort identified |
| **B.1 – fT4**  **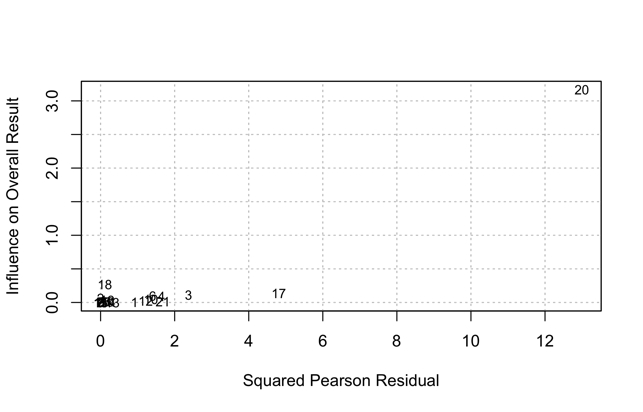** | | **B.2 – fT4**  **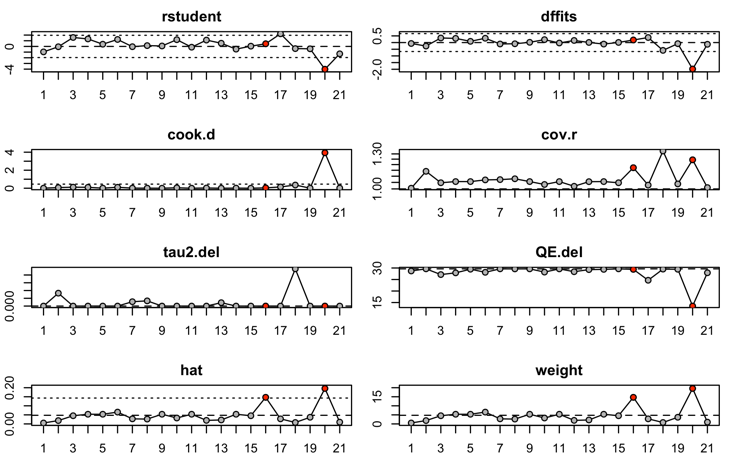** |
| 1. Chakrabarti, 2016^58^ 2. Duntas, 2003^45^ 3. Eskes, 2014^46^ 4. Esposito, 2017^52^ 5. Gärtner, 2002^25^ 6. Hu, 2021^6^ 7. Kachouei, 2018^55^ 8. Karanikas, 2008^40^ 9. Krysiak, 2012.1^8^ 10. Krysiak, 2012.2^8^ 11. Kyrgios, 2019^10^ | 1. Mahmoodianfard, 2015.1^54^ 2. Mahmoodianfard, 2015.2^54^ 3. Nacamulli, 2010^39^ 4. Negro, 2016^35^ 5. Pirola, 2016^42^ 6. Shabalina, 2019.1^47^ 7. Shabalina, 2019.2^47^ 8. Wang, 2018.1^19^ 9. Wang, 2018.2^19^ 10. Zhang, 2020^37^ | Outlier cohort (Pirola, 2016^42^, Wang, 2018.2^19^) removed   |

| **C.1 – fT3**  **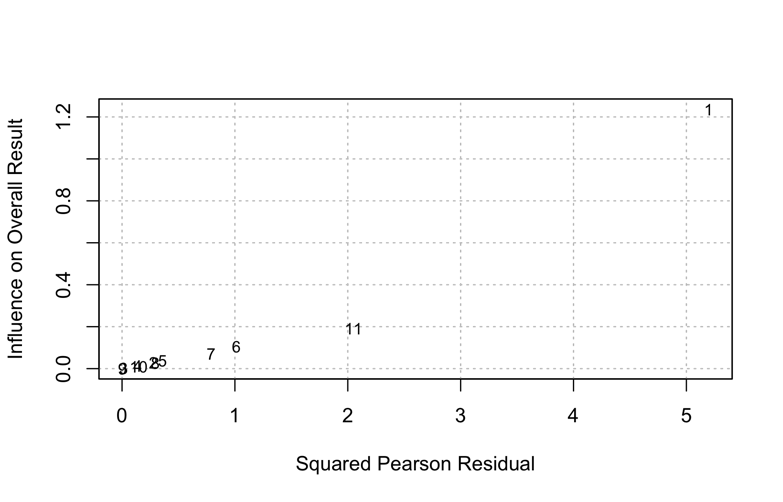** | | | **C.2 – fT3**  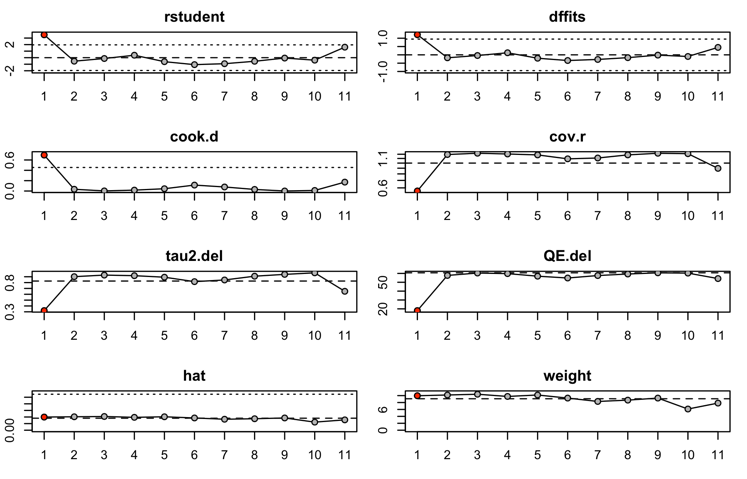 |
| --- | --- | --- | --- |
| 1. Esposito, 2017^52^ 2. Gärtner, 2002^25^ 3. Hu, 2021^6^ 4. Kachouei, 2018^55^ 5. Krysiak, 2012.1^8^ 6. Krysiak, 2012.2^8^ | | 1. Mahmoodianfard, 2015.1^54^ 2. Mahmoodianfard, 2015.2^54^ 3. Shabalina, 2019.1^47^ 4. Shabalina, 2019.2^47^ 5. Zhang, 2020^37^ | Outlier cohort (Esposito, 2017^52^) removed   |
| **D.1 – TPOAb**  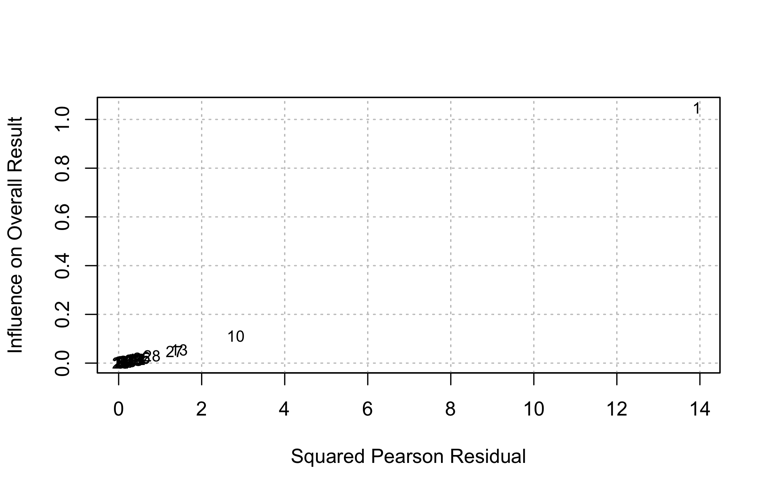 | | | **D.2 – TPOAb**  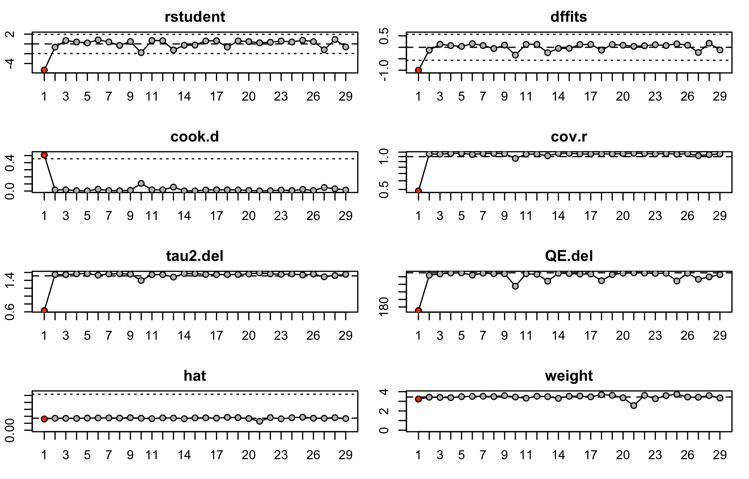 |
| 1. Balazs, 2009^56^ 2. Bhuyan, 2012^49^ 3. Bonfig, 2010^2^ 4. De Farias, 2015^44^ 5. Duntas, 2003^45^ 6. Eskes, 2014^46^ 7. Esposito, 2017^52^ 8. Gärtner, 2002^25^ 9. Hu, 2021^6^ 10. Kachouei, 2018^55^ 11. Karanikas, 2008^40^ 12. Karimi, 2019^50^ 13. Krysiak, 2012.1^8^ 14. Krysiak, 2012.2^8^ 15. Kyrgios, 2019^10^ | 1. Nacamulli, 2010^39^ 2. Pilli, 2015^4^ 3. Pirola, 2016^42^ 4. Preda, 2017^41^ 5. Shabalina, 2019.1^47^ 6. Shabalina, 2019.2^47^ 7. Sun, 2021^43^ 8. Tian, 2020^36^ 9. Turker, 2006^59^ 10. Wang, 2018.1^19^ 11. Wang, 2018.2^19^ 12. Zhang, 2020^37^ 13. Zhu, 2012.1^51^ 14. Zhu, 2012.2^51^ | | Outlier cohort (Balazs, 2009^56^) removed |
| **E.1 – TGAb**  **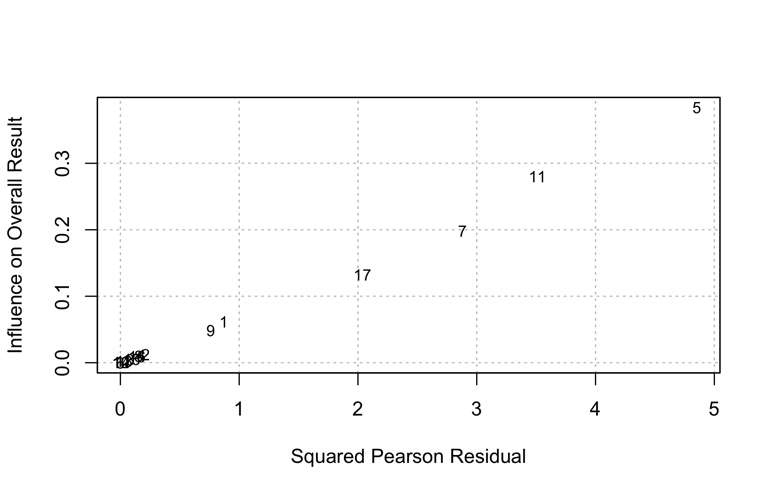** | | | **E.2 – TGAb**  **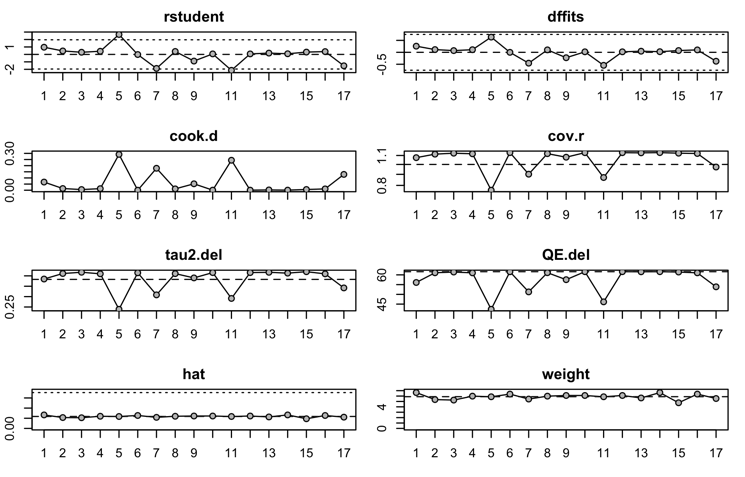** |
| 1. Balazs, 2009^56^ 2. Bonfig, 2010^2^ 3. De Farias, 2015^44^ 4. Duntas, 2003^45^ 5. Gärtner, 2002^25^ 6. Hu, 2021^6^ 7. Kachouei, 2018^55^ 8. Karimi, 2019^50^ 9. Krysiak, 2012.1^8^ 10. Krysiak, 2012.2^8^ | 1. Kyrgios, 2019^10^ 2. Nacamulli, 2010^39^ 3. Pilli, 2015^4^ 4. Sun, 2021^43^ 5. Tian, 2020^36^ 6. Turker, 2006^59^ 7. Zhang, 2020^37^ 8. Zhu, 2012.1^51^ 9. Zhu, 2012.2^51^ | | No outlier cohort identified |

**Figure S23. Outlier and influential case diagnostics using (1) visual inspection and (2) leave-one-out diagnostic for the outcomes (A) thyroid-stimulating hormone (TSH), (B) free thyroxine (fT4), (C) free triiodothyronine (fT3), (D) thyroid peroxidase antibodies (TPOAb), and (E) thyroglobulin antibodies (TGAb).** The leave-one-out diagnostics contained the externally standardized residual (rstudent), DFFITS value (dffits), Cook's distance (cook.d), covariance ratio (cov.r), leave-one-out amount of (residual) heterogeneity (tau2.del), leave-one-out test statistic of the test for (residual) heterogeneity (QE.del), and DFBETAS value (hat, weight). Red dots represent outlier cohorts with large residuals having a strong impact on the results according to Viechtbauer and Cheung (2010).^63^

**Table S6**. **Quality of evidence according to Grading of Recommendations Assessment, Development and Evaluation (GRADE)^64^ for all outcomes of the meta-analyses.**

| **Certainty assessment** | | | | | | | **№ of patients** | | **Effect** | | **Certainty** | **Importance** |
| --- | --- | --- | --- | --- | --- | --- | --- | --- | --- | --- | --- | --- |
| **N cohorts** | **Study design** | **Risk of bias** | **Inconsistency** | **Indirectness** | **Imprecision** | **Other considerations** | **Selenium** | **no treatment** | **Relative (95% CI)** | **Absolute (95% CI)** |  |  |
| **Thyroid-stimulating hormone (TSH)** | | | | | | | | | | | | |
| 26 | randomised trials | serious ^a^ | not serious ^c, e^ | not serious ^f^ | not serious ^g^ | none | 1092 | 971 | - | SMD **0.21 SD lower** (0.43 lower to 0.01 higher) | ⨁⨁⨁◯ Moderate | CRITICAL ^h^ |
| **Free thyroxine (fT4)** | | | | | | | | | | | | |
| 21 | randomised trials | serious ^a^ | not serious ^b^ | not serious ^f^ | not serious ^g^ | none | 864 | 800 | - | SMD **0.05 SD higher** (0.15 lower to 0.25 higher) | ⨁⨁⨁◯ Moderate | NOT IMPORTANT ^j^ |
| **Free trijodthyronine (fT3)** | | | | | | | | | | | | |
| 11 | randomised trials | serious ^a^ | very serious ^d^ | not serious ^f^ | not serious ^g^ | none | 330 | 328 | - | SMD **0.51 SD higher** (0.11 lower to 1.13 higher) | ⨁◯◯◯ Very low | NOT IMPORTANT ^j^ |
| **Thyroid peroxidase antibodies (TPOAb)** | | | | | | | | | | | | |
| 29 | randomised trials | serious ^a^ | serious ^d, e^ | not serious ^f^ | not serious ^g^ | none | 1233 | 1125 | - | SMD **0.96 SD lower** (1.36 lower to 0.56 lower) | ⨁⨁◯◯ Low | IMPORTANT ^h^ |
| **Thyroglobulin antibodies (TGAb)** | | | | | | | | | | | | |
| 17 | randomised trials | serious ^a^ | serious ^c^ | not serious ^f^ | not serious ^g^ | none | 686 | 597 | - | SMD **0.27 SD lower** (0.59 lower to 0.06 higher) | ⨁⨁◯◯ Low | NOT IMPORTANT ^j^ |
| **Adverse events** | | | | | | | | | | | | |
| 16 | randomised trials | serious ^a^ | not serious ^b^ | not serious ^f^ | not serious ^g^ | none | 698 | 641 | - | OR **0.89 higher** (0.46 higher to 1.75 higher) | ⨁⨁⨁◯ Moderate | CRITICAL ^h^ |

**CI:** confidence interval; **SMD:** standardized mean difference

#### Explanations a. Majority of the included studies was rated with a high risk of bias according to the Risk of Bias assessment; b. Heterogeneity (I^2^) was below 50%; c. Heterogeneity (I^2^) was between 50% and 74% and Inconsistency was rated serious; d. Heterogeneity (I^2^) was above 74% and Inconsistency was rated very serious; e. Inconsistency could be explained by subgroup analysis and inconsistency was rated less serious; f. Outcome answers directly clinical care question and Indirectness was rated not serious; g. Result was precise with small confidence intervals together with large information size and Imprecision was rated not serious; h. Outcome reflects directly Hashimoto thyroiditis status and safety of treatment and Importance was rated critical; i. Outcome reflects crucial disease progression parameter of Hashimoto thyroiditis and Importance was rated important; j. Quantification of the outcome is not well harmonized across different laboratories and correlation with Hashimoto thyroiditis disease status is less established. Importance was rated not important

**Table 7.** **Risk of Bias assessment of the studies investigating thyroid-stimulating hormone levels according to Cochrane risk-of-bias for randomized trials (ROB 2) tool.**

| **Author, Year** | **D1** | **D2** | **D3** | **D4** | **D5** | **Overall Bias** |
| --- | --- | --- | --- | --- | --- | --- |
| Anastasilakis, 2012 ^33^ | ! | + | - | + | ! | **-** |
| Balazs, 2009 ^56^ | + | + | + | + | ! | **!** |
| Bonfig, 2010 ^2^ | ! | + | + | + | ! | **!** |
| Chakrabarti, 2016 ^58^ | - | - | + | + | ! | **-** |
| De Farias, 2015 ^44^ | ! | + | - | + | ! | **-** |
| Duntas, 2003 ^45^ | ! | - | + | + | ! | **-** |
| Eskes, 2014 ^46^ | + | + | + | + | ! | **!** |
| Esposito, 2017 ^52^ | + | - | - | + | ! | **-** |
| Gärtner, 2002 ^25^ | + | + | + | + | ! | **!** |
| Hu, 2021 ^6^ | ! | - | - | + | + | **-** |
| Kachouei, 2018 ^55^ | ! | + | + | + | ! | **!** |
| Karanikas, 2008 ^40^ | ! | + | - | + | ! | **-** |
| Karimi, 2019 ^50^ | ! | + | + | + | ! | **!** |
| Krysiak, 2012 ^8^ | + | + | + | + | ! | **!** |
| Kyrgios, 2019 ^10^ | ! | + | + | + | + | **!** |
| Mahmoodianfard, 2015 ^54^ | ! | + | + | + | ! | **!** |
| Mahmoudi, 2021 ^12^ | ! | + | + | + | - | **-** |
| Mantovani, 2019 ^38^ | ! | + | - | + | + | **-** |
| Mao, 2016 ^34^ | ! | + | + | + | ! | **!** |
| Nacamulli, 2010 ^39^ | ! | - | + | + | ! | **-** |
| Negro, 2016 ^35^ | ! | + | + | + | ! | **!** |
| Pilli, 2015 ^4^ | ! | + | - | + | ! | **-** |
| Pirola, 2016 ^42^ | ! | - | + | + | ! | **-** |
| Preda, 2017 ^41^ | - | + | + | + | ! | **-** |
| Shabalina, 2019 ^47^ | ! | - | + | + | ! | **-** |
| Sun, 2021 ^43^ | ! | - | - | + | ! | **-** |
| Tian, 2020 ^36^ | ! | - | + | + | ! | **-** |
| Turker, 2006 ^59^ | ! | - | - | + | ! | **-** |
| Wang, 2018 ^19^ | ! | + | + | + | ! | **!** |
| Wu, 2018 ^53^ | ! | - | + | ! | - | **-** |
| Zhu, 2012 ^51^ | ! | + | - | + | ! | **-** |

D1= Domain 1 Randomization process; D2 = Domain 2 Deviation from intended intervention; D3 = Domain 3 Missing outcome data; D4 = Domain 4 Measurement of the outcome; D5 = Selection of reported results; TPOAb = Thyroid peroxidase antibodies; TSH = Thyroid-stimulating hormone; - = High risk of bias; ! = Some concerns; + = Low risk of bias.

| **A.1 TSH – All studies**  **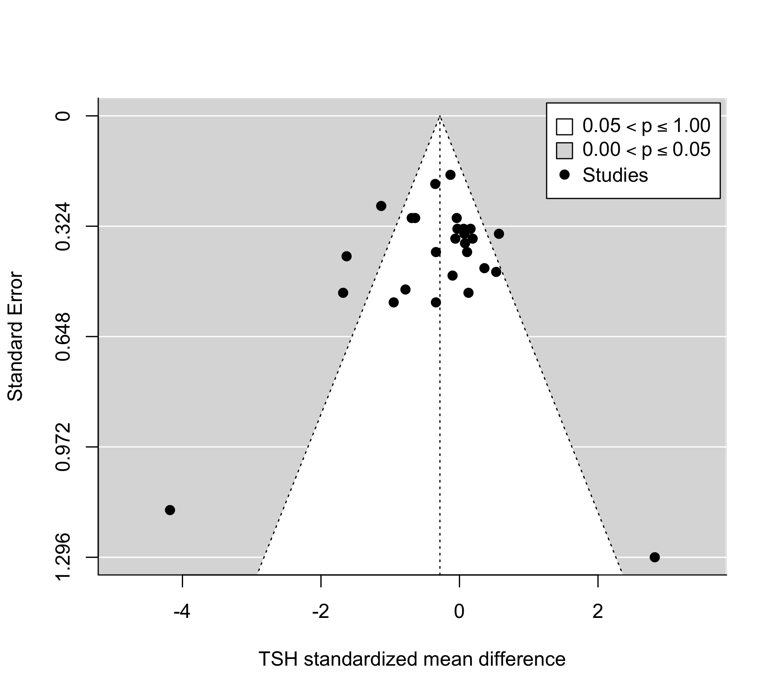** | **A.2 TSH – Studies without thyroid hormone replacement therapy** **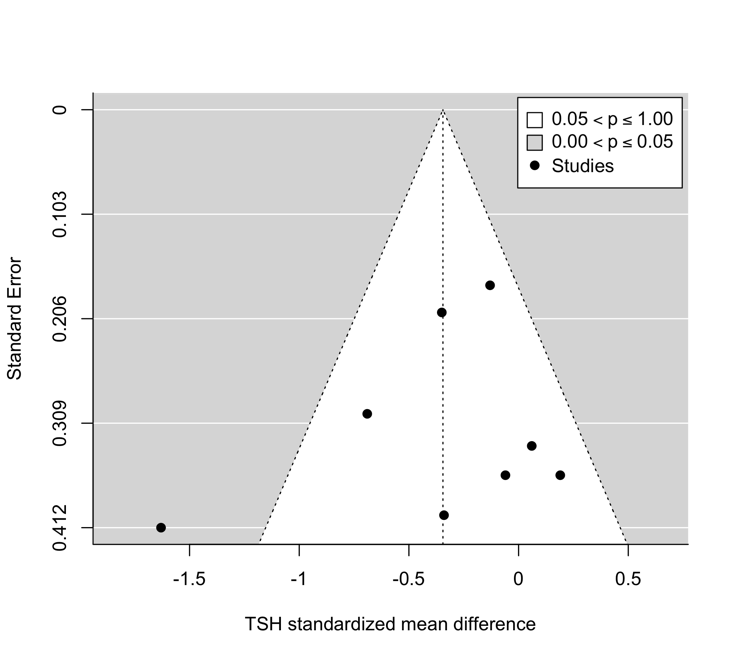** |
| --- | --- |
|  | **** |
| **B.1 FT4 – All studies**  **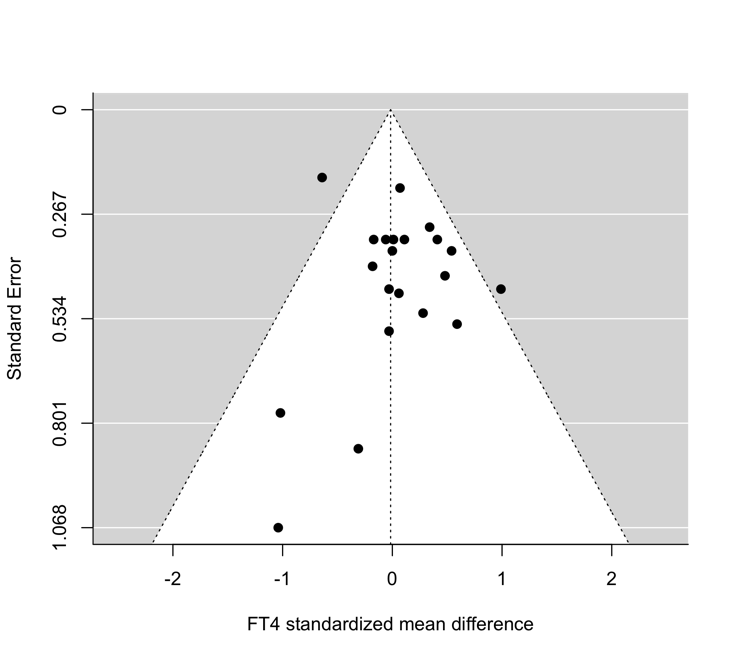** | **B.2 FT4 - Studies without thyroid hormone replacement therapy** **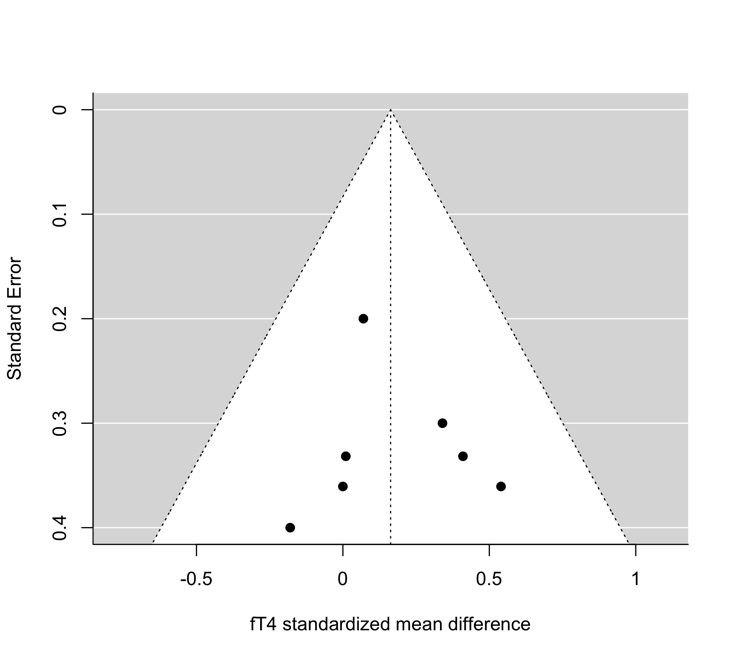** |
| **** | All studies within the white area (p >0.05) |
| **C.1 FT3 – All studies**  **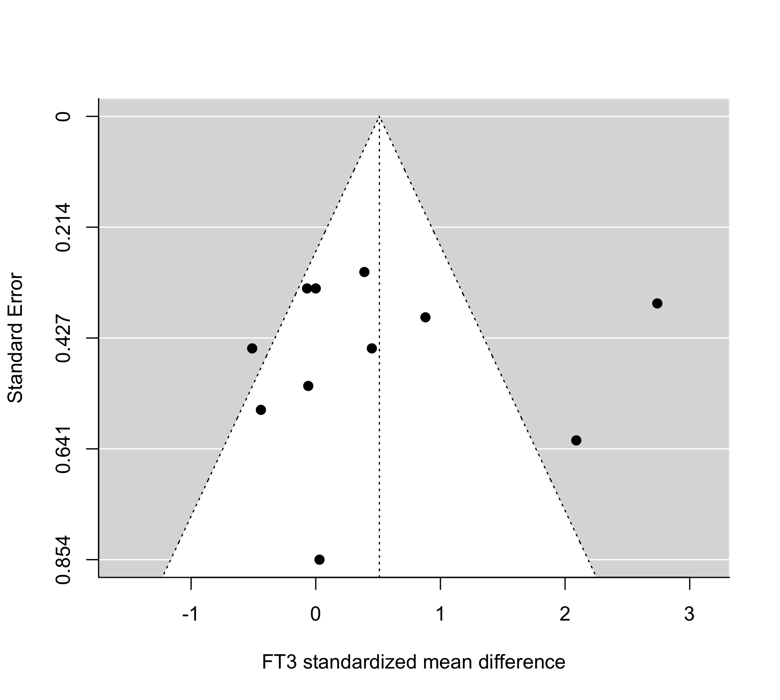** | **C.2 FT3 - Studies without thyroid hormone replacement therapy 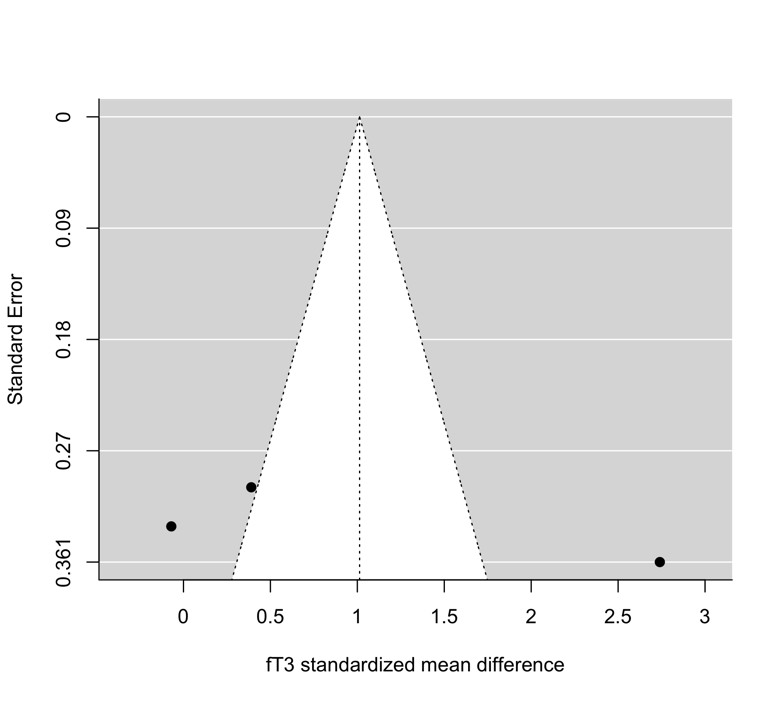** |
| **** | All studies within the grea area (p ≤0.05) |
| **D TPOAb – All studies**  **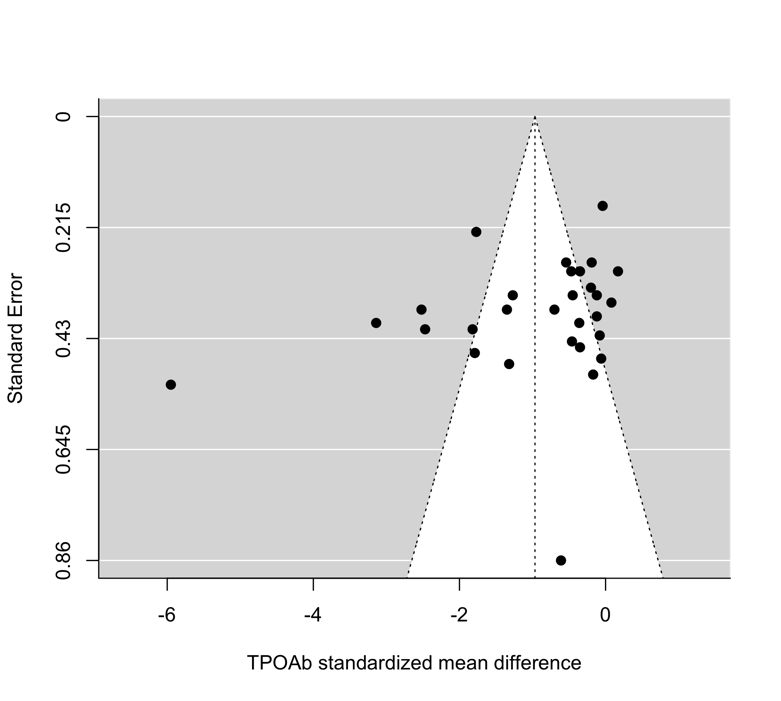** | **E TGAb – All studies 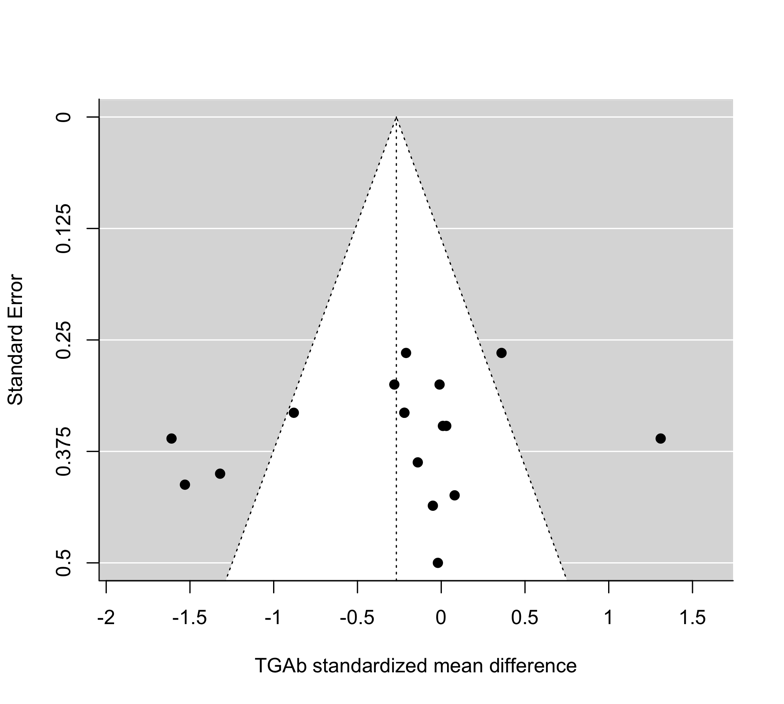** |
| **** | **** |
| **F Adverse events – All studies 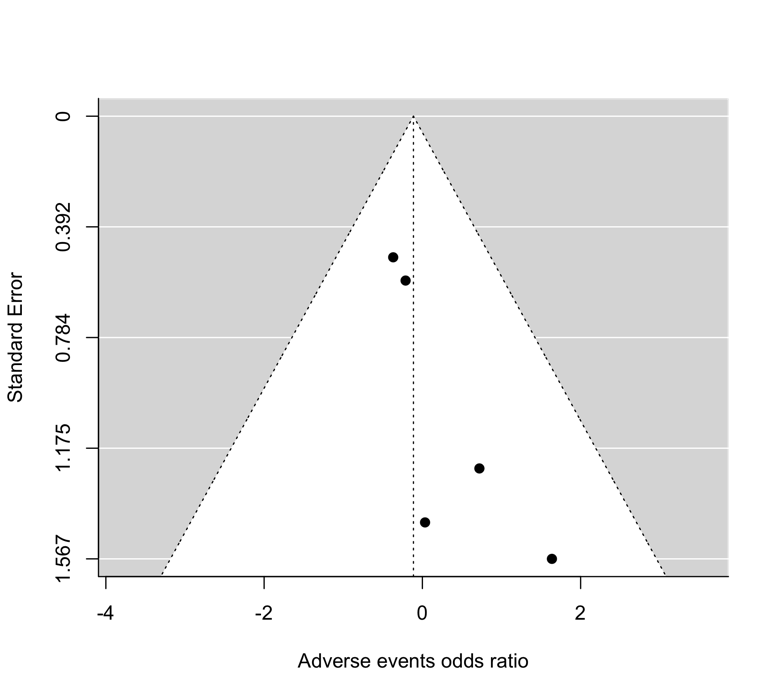** |  |
| All studies within the white area (p >0.05) |  |

**Figure S24. Funnel plots for detection of publication bias for the outcomes (A) thyroid-stimulating hormone (TSH), (B) free triiodothyronine (fT3), (C) free thyroxine (fT4), (D) thyroid peroxidase antibodies (TPOAb), (E) thyroglobulin antibodies (TGAb), and (F) adverse events.** A-E. The standardized mean differences are plotted against the standard error of the standardized mean differences. F. The log odds ratios are plotted against the standard error of the log odds ratios. The dashed lines depict the logarithm of the summary standardized mean difference or odds ratio with its 95% confidence interval (1) Funnel plots of the abovementioned outcomes, for all studies. (2) Funnel plots of the abovementioned outcomes, only for the studies performed in patients without thyroid hormone replacement.

# References

1. Bonfig W, Schmidt H. Selenium supplementation does not decrease thyroid peroxidase antibodies concentration in children and adolescents with autoimmune thyroiditis. Hormone Research in Paediatrics 2010;74(87, doi:10.1159/000321348

2. Bonfig W, Gärtner R, Schmidt H. Selenium supplementation does not decrease thyroid peroxidase antibody concentration in children and adolescents with autoimmune thyroiditis. ScientificWorldJournal 2010;1(10):990-996, doi:10.1100/tsw.2010.91

3. Cenci V, Pilli T, Cardinale S, et al. Selenomethionine supplementation in euthyroid patients with autoimmune thyroiditis: Effects of two doses (80 or 160 μg) versus placebo. European Thyroid Journal 2013;2(134), doi:10.1159/000352096

4. Pilli T, Cantara S, Schomburg L, et al. IFNγ-Inducible Chemokines Decrease upon Selenomethionine Supplementation in Women with Euthyroid Autoimmune Thyroiditis: Comparison between Two Doses of Selenomethionine (80 or 160 μg) versus Placebo. European Thyroid Journal 2015;4(4):226-233, doi:10.1159/000439589

5. Hu Y, Feng W, Chen H, et al. Effect of selenium on thyroid autoimmunity and regulatory T cells in patients with Hashimoto's thyroiditis: A prospective randomized‐controlled trial. Clinical and … 2021, doi:10.1111/cts.12993

6. Hu Y, Feng W, Chen H, et al. Effect of selenium on thyroid autoimmunity and regulatory T cells in patients with Hashimoto’s thyroiditis: A prospective randomized‐controlled trial. Clinical and Translational Science 2021;14(4):1390-1402

7. Krysiak R, Okopień B. The effect of levothyroxine and selenomethionine on lymphocyte and monocyte cytokine release in women with Hashimoto's thyroiditis. The Journal of clinical endocrinology and metabolism 2011;96(7):2206-2215, doi:10.1210/jc.2010-2986

8. Krysiak R, Okopień B. Haemostatic effects of levothyroxine and selenomethionine in euthyroid patients with Hashimoto's thyroiditis. Thrombosis and haemostasis 2012;108(5):973-980, doi:10.1160/TH12-04-0275

9. Kyrgios I, Giza S, Kotanidou EP, et al. L-selenomethionine supplementation in children and adolescents with autoimmune thyroiditis: A randomized double-blind placebo-controlled clinical trial. Journal of Clinical Pharmacy and Therapeutics 2019;44(1):102-108, doi:10.1111/jcpt.12765

10. Kyrgios I, Giza S, Kotanidou EP, et al. l‐selenomethionine supplementation in children and adolescents with autoimmune thyroiditis: A randomized double‐blind placebo‐controlled clinical trial. Journal of clinical pharmacy and therapeutics 2018;44(1):102-108, doi:10.1111/jcpt.12765

11. Kyrgios I, Dimopoulou A, Kotanidou E, et al. L-selenomethionine supplementation in children and adolescents with autoimmune thyroiditis: Preliminary results of a randomized double-blinded placebo-controlled clinical trial. Hormone Research in Paediatrics 2016;86(498, doi:10.1159/000449142

12. Mahmoudi L, Mobasseri M, Ostadrahimi A, et al. Effect of selenium-enriched yeast supplementation on serum thyroid-stimulating hormone and anti-thyroid peroxidase antibody levels in subclinical hypothyroidism: Randomized controlled trial. Adv 2021;27(10):33, doi:10.4103/abr.abr_252_20

13. Negro R. Postpartum thyroiditis. Endocrine Abstracts 2010;20(S26.3

14. Negro R, Greco G, Mangieri T, et al. The influence of selenium supplementation on postpartum thyroid status in pregnant women with thyroid peroxidase autoantibodies. The Journal of clinical endocrinology and metabolism 2007;92(4):1263-1268, doi:10.1210/jc.2006-1821

15. Pilli T, Cantara S, Schomburg L, et al. IFNγ-Inducible chemokines decrease upon selenomethionine supplementation in women with euthyroid autoimmune thyroiditis: comparison between two doses of selenomethionine (80 or 160 μg) versus placebo. European Thyroid Journal 2015;4(4):226‐233, doi:10.1159/000439589

16. Pilli T, Cantara S, Cenci V, et al. IFN gamma-Inducible Chemokines Are Down-Modulated By Selenomethionine (Semet) Supplementation in Women with Euthyroid Chronic Autoimmune Thyroiditis (AIT): Comparison Between 2 Doses of Semet (80 mu g or 160 mu g) Versus Placebo. Endocrine Reviews 2014;35(3):

17. Pilli T, Cantara S, Cenci V, et al. Ifnγ-inducible chemokines are down-modulated by selenomethionine (SEMET) supplementation in women with euthyroid chronic autoimmune thyroiditis (AIT): Comparison between 2 doses of semet (80 μg or 160 μg) versus placebo. Endocrine Reviews 2014;35(

18. Pilli T, Sandro C, Cenci V, et al. Effects of 2 doses (80 or 160 lg) of selenomethionine supplementation versus placebo in euthyroid female patients with autoimmune thyroiditis. Thyroid 2013;23(A50-A51, doi:10.1089/thy.2013.2310.abs

19. Wang W, Mao J, Zhao J, et al. Decreased Thyroid Peroxidase Antibody Titer in Response to Selenium Supplementation in Autoimmune Thyroiditis and the Influence of a Selenoprotein P Gene Polymorphism: A Prospective, Multicenter Study in China. Thyroid 2018;28(12):1674-1681, doi:https://dx.doi.org/10.1089/thy.2017.0230

20. Thomson CD, Campbell JM, Miller J, et al. Minimal impact of excess iodate intake on thyroid hormones and selenium status in older New Zealanders. European Journal of Endocrinology 2011;165(5):745-752, doi:10.1530/eje-11-0575

21. Kvicala J, Hrdá P, Zamrazil V, et al. Effect of selenium supplementation on thyroid antibodies. Journal of Radioanalytical and Nuclear Chemistry 2009;280(2):275-279, doi:10.1007/s10967-009-0510-z

22. Berisha-Muharremi V, Tahirbegolli B, Phypers R, et al. Efficacy of Combined Photobiomodulation Therapy with Supplements versus Supplements alone in Restoring Thyroid Gland Homeostasis in Hashimoto Thyroiditis: A Clinical Feasibility Parallel Trial with 6-Months Follow-Up. Journal of Personalized Medicine 2023;13(8):1274, doi:10.3390/jpm13081274

23. Filipowicz D, Szczepanek-Parulska E, Kłobus M, et al. Selenium Status and Supplementation Effects in Pregnancy-A Study on Mother-Child Pairs from a Single-Center Cohort. Nutrients 2022;14(15), doi:10.3390/nu14153082

24. Gärtner R, Gasnier BC. Selenium in the treatment of autoimmune thyroiditis. Biofactors 2003;19(3-4):165-70, doi:10.1002/biof.5520190309

25. Gärtner R, Gasnier BCH, Dietrich JW, et al. Selenium Supplementation in Patients with Autoimmune Thyroiditis Decreases Thyroid Peroxidase Antibodies Concentrations. The Journal of Clinical Endocrinology and Metabolism 2002;87(4):1687-1691, doi:10.1210/jcem.87.4.8421

26. Kryczyk-Kozioł J, Prochownik E, Błażewska-Gruszczyk A, et al. Assessment of the Effect of Selenium Supplementation on Production of Selected Cytokines in Women with Hashimoto's Thyroiditis. Nutrients 2022;14(14), doi:10.3390/nu14142869

27. Krysiak R, Kowalcze K, Szkrobka W, et al. Sexual Function and Depressive Symptoms in Young Women with Euthyroid Hashimoto's Thyroiditis Receiving Vitamin D, Selenomethionine and Myo-Inositol: A Pilot Study. Nutrients 2023;15(12), doi:10.3390/nu15122815

28. Mazokopakis EE, Papadakis JA, Papadomanolaki MG, et al. Effects of 12 Months Treatment with l-Selenomethionine on Serum Anti-TPO Levels in Patients with Hashimoto's Thyroiditis. Thyroid 2007;17(7):609-612, doi:10.1089/thy.2007.0040

29. Neto JR, Nogueira De Pontes AA. Effects of selenium supplementationonserum thyroid peroxidase antibody levels in patients with autoimmune thyroiditis. Thyroid 2015;25(A56), doi:10.1089/thy.2015.29004.abstracts

30. Nordio M, Pajalich R. Research Article Combined Treatment with Myo-Inositol and Selenium Ensures Euthyroidism in Subclinical Hypothyroidism Patients with Autoimmune Thyroiditis. Journal of Thyroid Research 2013;2013(2013):424163, doi:10.1155/2013/424163

31. Sun CP, Zhu M, Li L, et al. Effect of levothyroxine sodium combined with selenium supplement. Int J Clin Exp Med 2020;13(7):4695-4703

32. Gartner R, Duntas LH. Effects of selenium supplementation on TPOAb and cytokines in acute autoimmune thyroiditis. Thyroid 2008;18(6):669-70; author reply 673-4, doi:10.1089/thy.2008.0001

33. Anastasilakis AD, Toulis KA, Nisianakis P, et al. Selenomethionine treatment in patients with autoimmune thyroiditis: a prospective, quasi-randomised trial. Int J Clin Pract 2012;66(4):378-383, doi:10.1111/j.1742-1241.2011.02879.x

34. Mao J, Pop VJ, Bath SC, et al. Effect of low-dose selenium on thyroid autoimmunity and thyroid function in UK pregnant women with mild-to-moderate iodine deficiency. Eur J Nutr 2016;55(1):55-61, doi:10.1007/s00394-014-0822-9

35. Negro R, Schwartz A, Stagnaro-Green A. Impact of Levothyroxine in Miscarriage and Preterm Delivery Rates in First Trimester Thyroid Antibody-Positive Women With TSH Less Than 2.5 mIU/L. The Journal of Clinical Endocrinology and Metabolism 2016;101(10):3685-3690, doi:10.1210/jc.2016-1803

36. Tian X, Li N, Su R, et al. Selenium supplementation may decrease thyroid peroxidase antibody titer via reducing oxidative stress in euthyroid patients with autoimmune thyroiditis. International Journal of Endocrinology 2020;30(2020):9210572, doi:10.1155/2020/9210572

37. Zhang L, Sun X, Yan S, et al. Effects Of Levothyroxine Sodium Tablets Combined With Sodium Selenite On Autoimmune Antibodies, Thyroxine And T Lymphocyte Subsets In Patients With Hashimoto's Thyroiditis. Acta Medica Mediterranea 2020;36(3):1527-1531, doi:10.19193/0393-6384_2020_3_238

38. Mantovani G, Isidori AM, Moretti C, et al. Selenium supplementation in the management of thyroid autoimmunity during pregnancy: results of the "SERENA study", a randomized, double-blind, placebo-controlled trial. Endocrine 2019;66(3):542-550, doi:10.1007/s12020-019-01958-1

39. Nacamulli D, Mian C, Petricca D, et al. Influence of physiological dietary selenium supplementation on the natural course of autoimmune thyroiditis. Clin Endocrinol (Oxf) 2010;73(4):535-539, doi:10.1111/j.1365-2265.2009.03758.x

40. Karanikas G, Schuetz M, Kontur S, et al. No immunological benefit of selenium in consecutive patients with autoimmune thyroiditis. Thyroid 2008;18(1):7-12, doi:10.1089/thy.2007.0127

41. Preda C, Vasiliu I, Mihalache L, et al. Selenium-Essential Antioxidant Element The example of autoimune thyroiditis. Revista de Chimie 2017;68(7):1617-1621

42. Pirola I, Gandossi E, Agosti B, et al. Selenium supplementation could restore euthyroidism in subclinical hypothyroid patients with autoimmune thyroiditis. Endokrynologia Polska 2016;67(6):567-571, doi:10.5603/ep.2016.0064

43. Sun C, Zhu M, Li L, et al. Clinical Observation of Levothyroxine Sodium Combined with Selenium in the Treatment of Patients with Chronic Lymphocytic Thyroiditis and Hypothyroidism and the Effects on Thyroid Function, Mood, and Inflammatory Factors. Evidence-Based Complementary and Alternative Medicine 2021;2021(5471281), doi:10.1155/2021/5471281

44. De Farias C, Cardoso B, De Oliveira G, et al. A randomized-controlled, double-blind study of the impact of selenium supplementation on thyroid autoimmunity and inflammation with focus on the GPx1 genotypes. Journal of Endocrinological Investigation 2015;38(10):1065-1074, doi:10.1007/s40618-015-0285-8

45. Duntas LH, Mantzou E, Koutras DA. Effects of a six month treatment with selenomethionine in patients with autoimmune thyroiditis. European Journal of Endocrinology 2003;148(4):389-393, doi:10.1530/eje.0.1480389

46. Eskes SA, Endert E, Fliers E, et al. Selenite supplementation in euthyroid subjects with thyroid peroxidase antibodies. Clin Endocrinol (Oxf) 2013;80(3):444-451, doi:10.1111/cen.12284

47. Shabalina EA, Fadeyev VV. Effects of selenium in patients with autoimmune thyroiditis. Clinical and experimental thyroidology 2019;15(2):44-54

48. Aaseth J, Ellefsen S, Alehagen U, et al. Diets and drugs for weight loss and health in obesity - An update. Biomed Pharmacother 2021;140(111789, doi:10.1016/j.biopha.2021.111789

49. Bhuyan AK, Sarma D, Saikia UK. Selenium and the thyroid: A close-knit connection. Indian Journal of Endocrinology and Metabolism 2012;16(Suppl 2):S354-5, doi:10.4103/2230-8210.104090

50. Karimi F, Omrani GR. Effects of selenium and vitamin C on the serum level of antithyroid peroxidase antibody in patients with autoimmune thyroiditis. Journal of Endocrinological Investigation 2018;42(4):481-487, doi:10.1007/s40618-018-0944-7

51. Zhu L, Bai X, Teng W, et al. Effects of selenium supplementation on antibodies of autoimmune thyroiditis. Zhonghua Yi Xue Za Zhi 2012;92(32):2256-2260

52. Esposito D, Rotondi M, Accardo G, et al. Influence of short-term selenium supplementation on the natural course of Hashimoto’s thyroiditis: clinical results of a blinded placebo-controlled randomized prospective trial. Journal of Endocrinological Investigation 2016;40(1):83-89, doi:10.1007/s40618-016-0535-4

53. Wu D, Jin L, Xu H. Clinical effects of selenium yeast and levothyroxine combined therapy on patients with lymphocytic thyroiditis. Biomedical Research 2018;29(1):181-184

54. Mahmoodianfard S, Vafa M, Golgiri F, et al. Effects of Zinc and Selenium Supplementation on Thyroid Function in Overweight and Obese Hypothyroid Female Patients: A Randomized Double-Blind Controlled Trial. J Am Coll Nutr 2015;34(5):391-399, doi:10.1080/07315724.2014.926161

55. Kachouei A, Rezvanian H, Amini M, et al. The Effect of Levothyroxine and Selenium versus Levothyroxine Alone on Reducing the Level of Anti-thyroid Peroxidase Antibody in Autoimmune Hypothyroid Patients. Adv 2018;27(7):1-1, doi:10.4103/2277-9175.223735

56. Balázs C. The effect of selenium therapy on autoimmune thyroiditis. Orv Hetil 2008;149(26):1227-1232, doi:10.1556/oh.2008.28408

57. Yu L, Zhou L, Xu E, et al. Levothyroxine monotherapy versus levothyroxine and selenium combination therapy in chronic lymphocytic thyroiditis. Journal of Endocrinological Investigation 2017;40(11):1243-1250, doi:10.1007/s40618-017-0693-z

58. Chakrabarti SK, Ghosh S, Banerjee S, et al. Oxidative stress in hypothyroid patients and the role of antioxidant supplementation. Indian journal of endocrinology and metabolism 2016;20(5):674, doi:10.4103/2230-8210.190555

59. Turker O, Kumanlioglu K, Karapolat I, et al. Selenium treatment in autoimmune thyroiditis: 9-month follow-up with variable doses. The Journal of Endocrinology 2006;190(1):151-156, doi:10.1677/joe.1.06661

60. Nève J. Human selenium supplementation as assessed by changes in blood selenium concentration and glutathione peroxidase activity. J Trace Elem Med Biol 1995;9(2):65-73, doi:10.1016/s0946-672x(11)80013-1

61. Thomson CD. Assessment of requirements for selenium and adequacy of selenium status: a review. Eur J Clin Nutr 2004;58(3):391-402, doi:10.1038/sj.ejcn.1601800

62. Rayman MP. Selenium and human health. Lancet 2012;379(9822):1256-1268, doi:10.1016/s0140-6736(11)61452-9

63. Viechtbauer W, Cheung MW. Outlier and influence diagnostics for meta-analysis. Research Synthesis Methods 2010;1(2):112-25, doi:10.1002/jrsm.11

64. Schünemann H BJ, Guyatt G, Oxman A, editors. GRADE handbook for grading quality of evidence and strength of recommendations. Updated October 2013. The GRADE Working Group, 2013. 2013. Available from: guidelinedevelopment.org/handbook. [Last Accessed; 18.05.2022].
